# A viral ADP-ribosyltransferase attaches RNA chains to host proteins

**DOI:** 10.1038/s41586-023-06429-2

**Published:** 2023-08-16

**Authors:** Maik Wolfram-Schauerte, Nadiia Pozhydaieva, Julia Grawenhoff, Luisa M. Welp, Ivan Silbern, Alexander Wulf, Franziska A. Billau, Timo Glatter, Henning Urlaub, Andres Jäschke, Katharina Höfer

**Affiliations:** 1 Max Planck Institute for Terrestrial Microbiology, Marburg, Germany; 2 Institute of Pharmacy and Molecular Biotechnology, Heidelberg University, Heidelberg, Germany; 3 Bioanalytical Mass Spectrometry, Max Planck Institute for Multidisciplinary Sciences, Göttingen, Germany; 4 Department of Clinical Chemistry, University Medical Center, Göttingen, Germany; 5 Cluster of Excellence “Multiscale Bioimaging: from Molecular Machines to Networks of Excitable Cells” (MBExC), Georg-August-University, Göttingen, Germany; 6 Center for Synthetic Microbiology (SYNMIKRO), Philipps-Universität Marburg, Marburg, Germany

**Keywords:** RNA, Phage biology, Proteomics, Post-translational modifications

## Abstract

The mechanisms by which viruses hijack the genetic machinery of the cells they infect are of current interest. When bacteriophage T4 infects *Escherichia* *coli*, it uses three different adenosine diphosphate (ADP)-ribosyltransferases (ARTs) to reprogram the transcriptional and translational apparatus of the host by ADP-ribosylation using nicotinamide adenine dinucleotide (NAD) as a substrate^1,2^. NAD has previously been identified as a 5′ modification of cellular RNAs^3–5^. Here we report that the T4 ART ModB accepts not only NAD but also NAD-capped RNA (NAD–RNA) as a substrate and attaches entire RNA chains to acceptor proteins in an ‘RNAylation’ reaction. ModB specifically RNAylates the ribosomal proteins rS1 and rL2 at defined Arg residues, and selected *E.* *coli* and T4 phage RNAs are linked to rS1 in vivo. T4 phages that express an inactive mutant of ModB have a decreased burst size and slowed lysis of *E.* *coli*. Our findings reveal a distinct biological role for NAD–RNA, namely the activation of the RNA for enzymatic transfer to proteins. The attachment of specific RNAs to ribosomal proteins might provide a strategy for the phage to modulate the host’s translation machinery. This work reveals a direct connection between RNA modification and post-translational protein modification. ARTs have important roles far beyond viral infections^6^, so RNAylation may have far-reaching implications.

## Abstract (web summary)

Bacteriophage T4 uses an enzyme known as ADP-ribosyltransferase ModB to modify the translational apparatus of bacteria it infects, not only by ADP-ribosylating proteins, but also by attaching entire RNA chains in a process known as RNAylation.

## Main

**Fig. 1:**
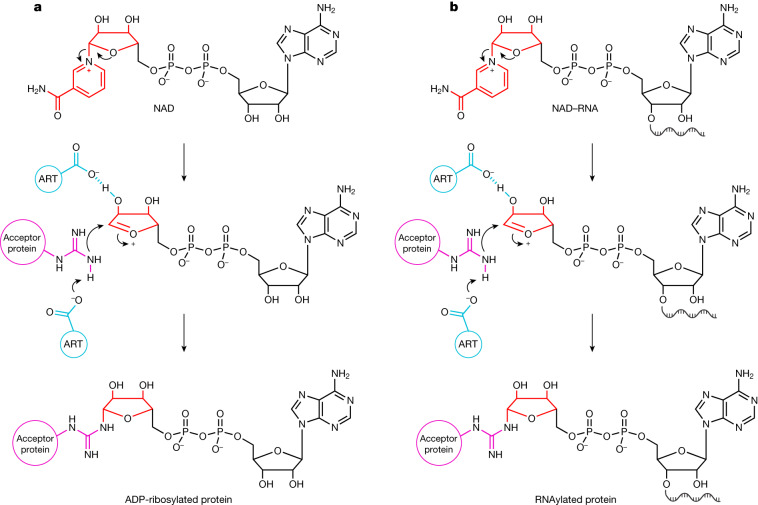
Mechanisms of ADP-ribosylation and proposed RNAylation. **a**, The mechanism of ADP-ribosylation for Arg. Initially, the N-glycosidic bond between the ribose and nicotinamide is destabilized by a Glu residue of an ART. This leads to the formation of an oxocarbenium ion of ADPr, with nicotinamide as the leaving group. This electrophilic ion is attacked by a nucleophilic Arg residue of the acceptor protein after Glu-mediated proton abstraction, leading to the formation of an N-glycosidic bond^50^. **b**, Our proposed RNAylation-reaction mechanism. In a similar way to ADP-ribosylation in the presence of NAD, we propose that ARTs might use NAD–RNA to catalyse an RNAylation reaction, thereby covalently attaching an RNA to an acceptor protein. Red, nicotinamide riboside of NAD and NAD-RNA; blue, catalytic residues of the ART; purple, nucleophilic Arg residue of the acceptor protein.

ARTs catalyse the transfer of one or multiple ADP–ribose (ADPr) units from NAD to target proteins^7^. Bacterial and archaeal ARTs act as toxins and are involved in host defence or drug-resistance mechanisms^8^, whereas eukaryotic ARTs have roles in distinct processes ranging from DNA damage repair to macrophage activation and stress response^9^. Viruses use ARTs as weapons to reprogram the host’s gene-expression system^6^. Mechanistically, a nucleophilic residue of the target protein (usually Arg, Glu, Asp, Ser or Cys) attacks the glycosidic carbon atom in the nicotinamide riboside moiety of NAD, forming a covalent bond as N-, O- or S-glycoside^7^ (Fig. 1a). As the adenosine moiety of NAD is not involved in this reaction, we speculated that elongation of the adenosine to long RNA chains (by means of regular 5′–3′ phosphodiester bonds) might be tolerated by ARTs, potentially leading to the formation of covalent RNA–protein conjugates (Fig. 1b). RNAs that have a 5′-NAD cap have previously been found in bacteria (including *E.* *coli*^3,10,11^), archaea^12,13^ and eukaryotes^5,14–19^, with NAD–RNA concentrations ranging from 1.9 to 7.4 fmol µg^−1^ RNA^16^. This modification was observed in different types of RNA, including mRNA and small regulatory RNA (sRNA)^20^. However, little is known about the biological functions of this RNA cap^21^.

The infection cycle of bacteriophage T4 relies on the sequential expression of early, middle and late phage genes that are transcribed by *E.* *coli* RNA polymerase (RNAP)^22^. For the specific temporal reprogramming of the *E.* *coli* transcriptional and translational apparatus, the T4 phage uses 3 ARTs that modify more than 30 host proteins. Upon infection, one of these ARTs, Alt, is injected into the bacterium with the phage DNA and immediately ADP-ribosylates *E.* *coli* RNAP at different residues, which is thought to result in the preferential transcription of phage genes from early promoters^23,24^. Two early phage genes encode the ARTs ModA^25^ and ModB^1,26^. ModA completes the ADP-ribosylation of RNAP, whereas ModB is thought to modify the host protein rS1 (refs. ^1,26^). However, it is still not known how ADP-ribosylation changes the properties of the target proteins, or whether other proteins are also modified during T4 infection.

## ModB catalyses RNAylation in vitro

**Fig. 2:**
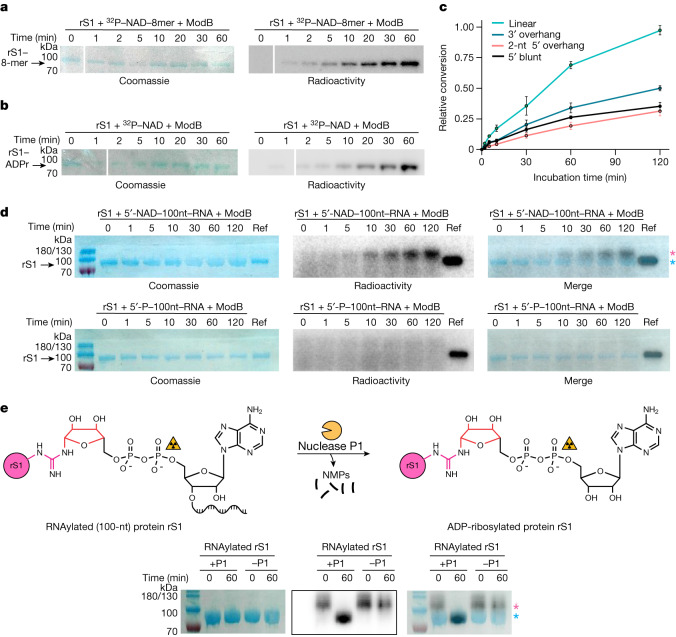
Post-translational protein modification of rS1 by ModB in vitro. **a**, Time course of the RNAylation of rS1 by ModB (*n* = 3). SDS–polyacrylamide gel electrophoresis (SDS–PAGE) gels are shown for rS1 + ^32^P–NAD–8-mer + ModB. Complete gels and a reaction schematic are shown in Extended Data Fig. 1b. **b**, Time course of the ADP-ribosylation of rS1 by ModB (*n* = 3), showing rS1 + ^32^P–NAD + ModB. Complete gels and a reaction schematic are shown in Extended Data Fig. 1c. rS1 RNAylation (**a**) and ADP-ribosylation (**b**) are indicated by the acquisition of a radioactive signal overlapping with the Coomassie stain. **c**, The role of RNA secondary structure on RNAylation reaction. Four different 3′ Cy5-labelled NAD-capped RNAs were tested, including a linear 10-mer NAD-capped RNA and three structured NAD-capped RNAs with a 3′ overhang, a dinucleotide 5′ overhang or a blunt end. SDS–PAGE analysis is shown in Extended Data Fig. 3a. Relative conversion refers to the intensity of the RNAylated rS1 band relative to the maximal RNAylation intensity observed among all four tests. Data points represent mean ± s.d. values based on quantification of fluorescence Cy5 signals (*n* = 3 biologically independent replicates). **d**, In vitro kinetics of the RNAylation of rS1 by ModB using 5′-NAD–100-nucleotide (100-nt) RNA as the substrate (top), analysed by SDS–PAGE. The pink asterisk indicates shifted RNAylated rS1; the blue asterisk indicates ADP-ribosylated rS1. ADP-ribosylated rS1 serves as a reference (Ref). The mass of 100 nucleotides is around 30 kDa; RNAylated rS1 has a mass of around 100 kDa (70 kDa from rS1, 30 kDa from RNA). 5′-P–100nt RNA was used as a negative control (bottom, *n* = 2). The two bands above the 100 kDa band are denoted 180/130. **e**, The nuclease P1 breaks down RNAylated protein rS1. The covalently attached 100-nucleotide-long RNA results in a shift of the RNAylated protein rS1 (which has a mass of around 100 kDa) in SDS–PAGE. Nuclease P1 cleaves the phosphodiester bond, resulting in degradation of the attached RNA into mononucleotides. Nuclease P1 converts RNAylated rS1 into ADP-ribosylated rS1 (mass of around 70 kDa), which can be seen by the presence of a downshifted protein band in the SDS–PAGE gel (*n* = 1). Red, ribose moiety of RNAylated/ADP-ribosylated protein; NMPs, nucleoside monophosphates; radioactivity symbol indicates site of ^32^P-label; pacman symbolizes nuclease P1. The pink and blue asterisks are the same as in **d**. Source data

**Extended Data Fig. 1:**
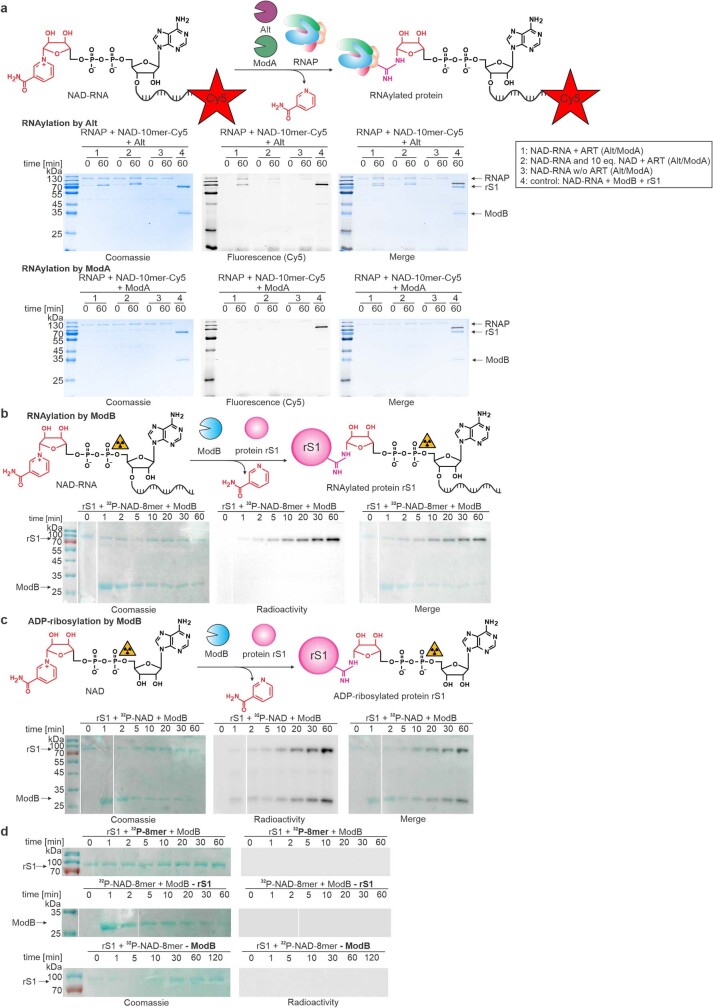
ADP-ribosylation and RNAylation by T4 ARTs. **a**, Characterisation of RNAylation of the RNA polymerase (RNAP) by the ARTs Alt or ModA in the presence of NAD-10mer-Cy5 (1), additional 10 equivalents of NAD (2) or in the absence of the respective ART (3) (n = 3). rS1 RNAylated with NAD-10mer-Cy5 by ModB serves as a reference (4). The RNAP is a well-established target protein of Alt and ModA and was thus chosen to assess RNAylation by Alt and ModA. Alt slightly RNAylates the RNAP *in vitro* which is abolished in the presence of 10 equivalents of NAD relative to NAD-10mer-Cy5. Protein load is visualised by Coomassie staining and RNAylated protein is visualised in the fluorescent Cy5 channel. **b**,**c**, Time course analysis of the ModB-mediated RNAylation (b) and ADP-ribosylation (c) of rS1 analysed by SDS-PAGE (n = 3 each). RNAylated or ADP-ribosylated protein is visualised by radioactivity scan and protein load confirmed by Coomassie staining. **d**, Negative controls for RNAylation of rS1 with ModB analysed by SDS-PAGE. RNAylation assay was performed in the presence of ^32^P-RNA lacking the NAD-cap (upper panel) and in the absence of either rS1 (- rS1) (middle panel) or ModB (-ModB) (lower panel) (n = 3). In these experiments, no RNAylation was detected in the radioactive scan of the SDS-PAGE gel.

**Extended Data Fig. 2:**
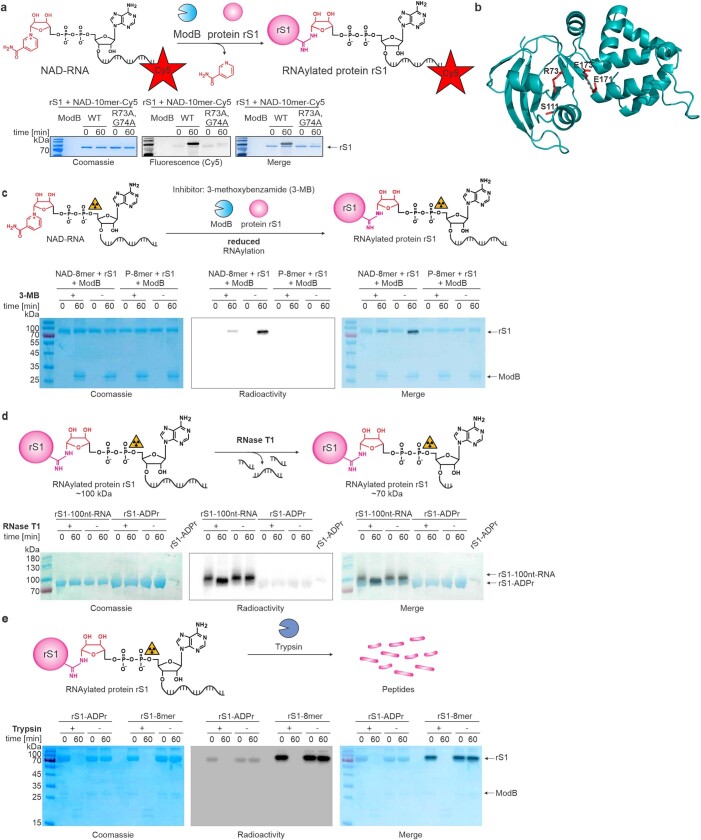
Characterisation of the RNAylation of protein rS1 by ModB. **a**, RNAylation of rS1 in the presence of catalytically active ModB and catalytically inactive ModB R73A, G74A with NAD-10mer-Cy5 (n = 3). In addition to the catalytically important residue R73, we mutated G74 as well. Mutation of G74 results in an altered PAM region, which is important for CRISPR-Cas9 gene editing of the T4 phage genome. **b**, AlphaFold prediction^63^ of the structure of ModB. Active site residues of the R-S-EXE motif^1^ are highlighted in red. Corresponding confidence metrics are shown in Supplementary Fig. 2. **c**, Inhibition of *in vitro* RNAylation of protein rS1 by ModB via ART inhibitor 3-MB. Reactions were performed with ^32^P-NAD-RNA 8mer (^32^P-NAD-8mer) as well as ^32^P-RNA 8mer (negative control) (n = 3). 3-MB reduces the yield of RNAylated rS1. **d**, *in vitro* digest of RNAylated and ADP-ribosylated protein rS1 by RNase T1. Reactions performed in the absence of RNase T1 (-) serve as negative controls. Protein rS1 ADP-ribosylated in the presence of ^32^P-NAD was applied as a reference (S1-ADPr) (n = 2). Upon T1 digest, the 100nt-RNA at rS1 is shortened, and the molecular weight of RNAylated rS1 is reduced. This leads to similar electrophoretic mobility as for ADP-ribosylated rS1. **e**, Tryptic digest of ADP-ribosylated and RNAylated protein rS1 (n = 2). The protein is degraded in the presence of trypsin, and RNAylation and ADP-ribosylation signals are lost. All samples were analysed by 12 % SDS-PAGE, protein was visualised by Coomassie staining and RNAylation was assessed via a radioactivity scan.

To test our idea that ARTs may accept NAD–RNAs as substrates, we purified Alt, ModA and ModB. We incubated them with either a synthetic, site-specific ^32^P-labelled 5′-NAD–RNA 8-base oligonucleotide (8-mer) or a 3′-fluorophore-labelled 5′-NAD–RNA 10-mer to test for either self-modification or the modification of target proteins. Whereas both Alt and ModA showed only a small amount of target RNAylation (Extended Data Fig. 1a), ModB rapidly RNAylated its known ADP-ribosylation target protein, rS1, without detectable self-RNAylation (Fig. 2a and Extended Data Fig. 1b). By contrast, ModB-mediated ADP-ribosylation in the presence of ^32^P-NAD resulted in the modification of both proteins (ModB and rS1) with similar intensity (Fig. 2b and Extended Data Fig. 1c). No signal was evident when either ModB or rS1 was missing, or when a 5′-^32^P-monophosphate–RNA (5′-^32^P–RNA) of the same sequence was used as a substrate for ModB (Extended Data Fig. 1d). Moreover, a mutated active site (R73A, G74A) of ModB also prevented the RNAylation of rS1 (ref. ^1^) (Extended Data Fig. 2a,b). This mutation similarly affected both the ADP-ribosylation and the RNAylation activity of ModB.

## RNAylation follows an ADP-ribosylation-like mechanism

ModB-catalysed RNAylation of rS1 was strongly inhibited by the ART inhibitor 3-methoxybenzamide (3-MB)^27^, which is thought to mimic the nicotinamide moiety (Extended Data Fig. 2c), confirming an ADP-ribosylation-like mechanism. Moreover, RNAylated rS1 proteins that carry a ^32^P-labelled ADPr moiety were treated with the ribonuclease (RNase) T1 to determine whether the RNA and the protein are covalently linked (Extended Data Fig. 2d). This treatment would remove the ^32^P label if the RNA were non-covalently bound to rS1 or covalently linked at any position other than the 5′-terminal positions. The ^32^P-rS1 signal did not disappear after treatment with T1, but it disappeared entirely after treatment with trypsin, which breaks down rS1 (Extended Data Fig. 2e). Collectively, these data indicate that the RNA is covalently linked to rS1 at its 5′ end, as shown in Fig. 1b.

**Extended Data Fig. 3:**
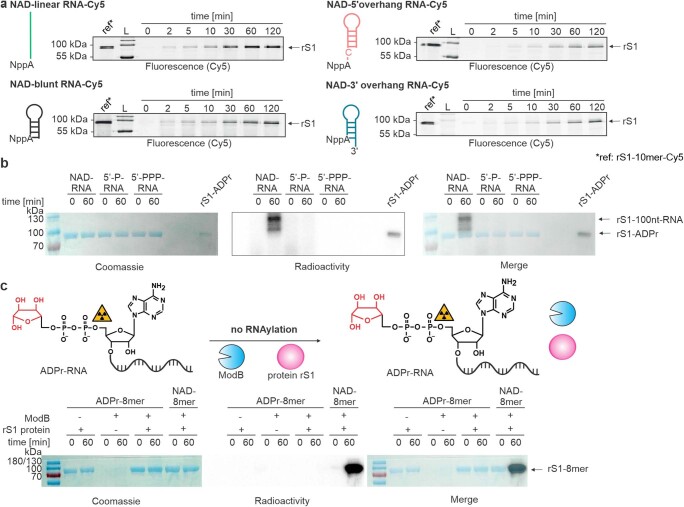
Characterisation of ModB mediated RNAylation *in vitro*. **a**, Analysis of the role of RNA secondary structure on RNAylation reaction. Four different 3′-Cy5-labelled NAD (NppA)-capped RNAs were tested including a linear (green) NAD-capped RNA (10mer) and three structured RNAs with either a 3′-overhang (blue), a 5′-overhang (red) or a blunt end (black) (n = 3). SDS-PAGE analyses of the time course of RNAylation are shown. Quantification of the signal intensities (Cy5 scan) relative to the reference is shown in Fig. 2c. ModB prefers linear 5′-ends of NAD-capped RNAs. L = ladder. **b**, Analysis of the RNAylation dependency on the presence of a 5′-NAD-cap of the RNA. 10 % SDS-PAGE analysis of *in vitro* RNAylation of the protein rS1 by ModB in the presence of either 5′-NAD-capped (NAD-RNA), 5′-monophosphate- (5′-P-RNA) or 5′-triphosphate-100nt-RNA (5′-PPP-RNA) (n = 2). RNAylation with radiolabelled RNA is detected by radioactivity scan and protein load visualised by Coomassie staining. *In vitro* RNAylation of rS1 is only observed in the presence of NAD-RNA. RNAylated rS1 cannot be detected by Coomassie staining due to low sensitivity. **c**, Characterisation of ADPr-RNA (which is lacking the nicotinamide moiety compared to NAD-RNA) as a substrate for ModB (n = 2). As a positive control, NAD-8mer was applied. All reactions were analysed by 12 % SDS-PAGE. RNAylation with radiolabelled RNA is detected by radioactivity scan and protein load visualised by Coomassie staining. ADPr-RNA is not accepted as a substrate for ModB-catalysed RNAylation *in vitro*.

RNAylation assays using short linear or hairpin-forming NAD–RNAs (Fig. 2c and Extended Data Fig. 3a) revealed that ModB has a preference for unstructured NAD–RNAs as a substrate, although it also accepted longer, biologically relevant NAD-capped RNAs as substrates, such as a NAD-capped Qβ RNA fragment of around 100 nucleotides^28^ (Fig. 2d and Extended Data Fig. 3b). RNAylation with NAD-capped 100-nucleotide RNA caused the modified rS1 protein to migrate with an apparent mass of 100 kDa (Fig. 2e). Treatment of the RNAylated protein with nuclease P1, which hydrolyses 3′–5′ phosphodiester bonds but does not attack the pyrophosphate bond of the 5′-ADPr, reversed this shift, and the ^32^P-labelled product migrated in a similar way to unmodified rS1 or ADPr–rS1 (Fig. 2e), confirming the proposed nature of the covalent linkage.

To exclude the possibility that ModB removes only the nicotinamide moiety from the NAD–RNA by hydrolysis, thereby generating a highly reactive ribosyl moiety that could (through its masked aldehyde group) spontaneously react with nucleophiles in its vicinity^29^, we prepared ADPr-modified RNA and tested it as a substrate for ModB. No modification could be detected (Extended Data Fig. 3c), providing no support for spontaneous RNAylation.

To exclude the degradation of RNA during RNAylation, we supplied ModB with an NAD–RNA 10-mer that carried a fluorescent dye (Cy5) at the 3′ terminus (Extended Data Figs. 2a and 3a). The time-course analysis of the RNAylation indicates that intact oligonucleotide chains were attached to rS1 for a variety of NAD-capped RNAs (Extended Data Fig. 3a).

## ModB modifies Arg residues in rS1

To identify the amino acid residues in protein rS1 to which RNA chains are covalently linked during RNAylation, we used tools developed to analyse protein ADP-ribosylation.

**Fig. 3:**
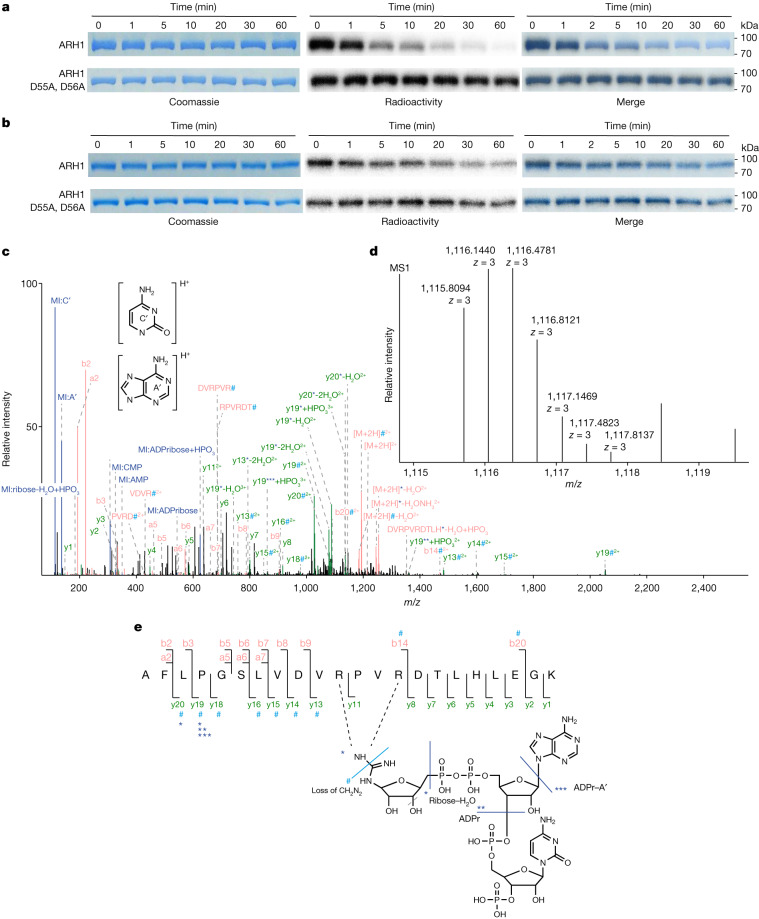
Identification of RNAylation sites of rS1. **a**,**b**, Specific removal of ADP-ribosylation and RNAylation by ARH1 (*n* = 3). Schematics of the reaction are shown in Extended Data Fig. 4c,d. Enzyme kinetics of ARH1 in the presence of ADP-ribosylated (**a**) or RNAylated (**b**) protein rS1 were analysed by SDS–PAGE. Mutation of the catalytically important residues D55 and D56 abolished the removal of ADP-ribosylation and RNAylation. **c**-**e**, Tandem MS-based identification of RNAylated rS1 peptide. **c**, The MS/MS fragment ion spectrum (spectrum ID: 23723) of RNAylated rS1 peptide AFLPGSLVDVRPVRDTLHLEGK carrying ADPr plus cytidine monophosphate and a 3′ phosphate group. The spectrum shows marker ions (MI) of adenine (A′) and cytosine (C′), adenosine monophosphate (AMP), cytidine monophosphate (CMP), ribose–H_2_O and ADPr. The precursor ion ([M + 2H]^2+^) and fragment ions y13–y16, y18–y20, b14 and b20 show a specific loss of mass of 42.021798 Da (#), which can be explained by the loss of CH_2_N_2_ at the modified Arg^31^. Precursor ions, y13, y19 and y20 are shifted by the mass of ribose–H_2_O (*). The spectrum also shows precursor ions and y19 being shifted by ADPr with (**) and without (***) the loss of adenine. Blue, MI; red, precursor ions, internal fragment ions, b-type fragment; green, y-type fragment ions. **d**, Isotopic peak pattern of the precursor ion as detected in the MS precursor ion scan for the MS/MS spectrum shown in **c**. **e**, Sequence and RNA adduct representation of the RNAylated peptide shown in **c** and **d**, including annotations of unshifted fragment ions and fragment ions showing arginine loss (#), as well as ribose–H_2_O (*), ADPr (**) and ADPr–adenine (***). The fragmentation products of the ADPr + CMP + 3′-phosphate adduct observed in the MS/MS spectrum shown in **c** are indicated in the structure by light blue (mass loss) and dark blue (mass adducts) lines.

**Extended Data Fig. 4:**
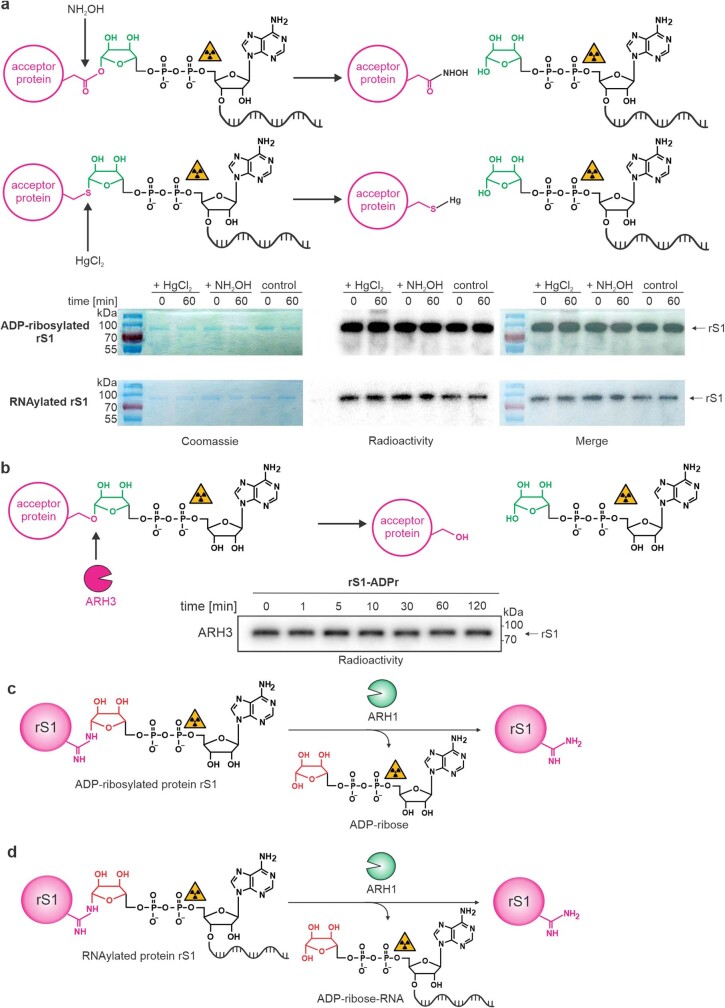
Specific removal of RNAylation using chemical and enzymatic treatments. **a**, Different ADP-ribose-protein linkages have been shown to be either stable or unstable in the presence of HgCl_2_ and neutral hydroxylamine (NH_2_OH), which represents a relatively straightforward and fast approach to identify ADP-ribosylation sites. Treatment with NH_2_OH hydrolyses linkages between glutamate/aspartate and ADP-ribose. HgCl_2_ specifically cleaves thiol-glycosidic bonds. ADP-ribosylated and RNAylated protein rS1 were treated with NH_2_OH or HgCl_2_. The removal of ADPr or RNA by these chemicals would result in a decrease of the radioactive signal of protein rS1. All samples were analysed by 12 % SDS-PAGE, stained in Coomassie (protein loading control) and RNAylation assessed as radioactivity. A decrease of the radioactive signal in comparison to the control (untreated) was not determined (n = 1). **b**, *in vitro* time course of the stability of rS1 ADP-ribosylation in the presence of ARH3 analysed by 12 % SDS-PAGE (n = 1). The autoradiography scan is presented. ARH3 did not remove the ADP-ribosylation. **c**-**d**, Reaction schematics for the removal of the ADP-ribosylation (**c**) and RNAylation (**d**) of rS1 by ARH1.

The radioactive signal of ^32^P-RNAylated protein rS1 and ^32^P–ADP-ribosylated rS1 did not change after treatment with HgCl_2_ (which cleaves S-glycosides at Cys residues), NH_2_OH (which hydrolyses O-glycosides at Asp and Glu) (Extended Data Fig. 4a) or recombinant enzyme ARH3 (which hydrolyses O-ADPr glycosides specifically at Ser residues) (Extended Data Fig. 4b), although it was efficiently removed by treatment with human ARH1 (Fig. 3a,b and Extended Data Fig. 4c,d). These findings indicate that the main products of ModB-catalysed RNAylation are linked as N-glycosides by Arg residues (Extended Data Fig. 4c,d).

To establish that ModB-mediated ADP-ribosylation or RNAylation also occurs at Arg residues in vivo, we isolated genomically His-tagged rS1 from non-infected or T4-infected *E.* *coli*. Analysis using liquid chromatography with tandem mass spectrometry (LC–MS/MS) confirmed that there was specific modification of Arg residues in rS1 with ADPr. These ADPr modifications were present only in the T4-infected sample (Extended Data Table 1 and Supplementary Table 1). R139 was identified as a modified residue, as confirmed by site-directed mutagenesis to Lys or Ala; rS1(R139K) and rS1(R139A) mutants were expressed in T4-infected *E.* *coli*, purified and analysed, revealing that these mutations prevent modification at those positions (Extended Data Table 2 and Supplementary Table 2).

## LC–MS/MS analysis verifies RNAylation

**Extended Data Fig. 5:**
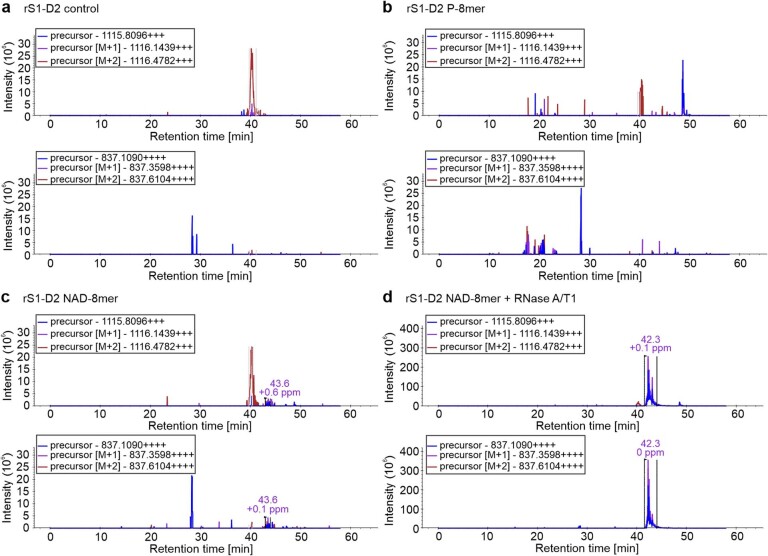
Ion chromatograms of unmodified rS1 and *in vitro* RNAylated rS1 extracted from LC-MS/MS data. Extracted ion chromatograms (XICs) for triply and quadruply charged precursor ions (monoisotopic masses 1115.8096 and 837.1090, respectively). XICs were extracted using Skyline^59^, an open source document editor for creating and analysing targeted proteomics experiments. The masses correspond to an rS1 peptide AFLPGSLVDVRPVRDTLHLEGK with an attached ADPr-cytidine. Recombinant S1 domain 2 was *in vitro* incubated with ModB and one of the following components: **a**, no other supplements, **b**, uncapped RNA-8mer, **c**, NAD-RNA-8mer, **d**, NAD-RNA-8mer treated with RNase A and T1 (results in ADPr-cytidine adducts). An elution peak at 42.3 min is observable in d and corresponds to the peptide modified with ADPr-cytosine. Spurious intensities can be observed in c and might represent a degradation product. **a** and **b** show only background/contaminant peaks. A contaminant peak at 40 min can be also observed in d (consider the difference in the intensity scale between **d** and **a**-**c**).

The LC–MS/MS analysis above did not show unambiguously that the modification of rS1 was derived from RNAylated or ADP-ribosylated rS1. We therefore optimized LC–MS/MS to detect the covalent attachment of RNA to rS1. For this analysis, in vitro RNAylated, truncated rS1 protein was subjected to an RNase A/T1 and tryptic digest. The obtained mixture was directly subjected to LC–MS/MS analysis, and MS data were evaluated using the RNPXL software tool^30^, on the assumption that the RNAylated rS1 peptide still has a trinucleotide (ADPr–cytidine) attached. The LC–MS/MS analysis this time showed the covalent attachment of a trinucleotide (ADPr–cytidine) to an rS1 peptide encompassing amino acid positions 129–150. Strikingly, the precursor mass ([M + 3H]^3+^ with a mass-to-charge ratio (*m*/*z*) = 1,115.81, expected molecular mass = 3,344.41 Da) plus the gas-phase b- and y-type fragmentation pattern, which shows the characteristic neutral loss of CH_2_N_2_ (derived from a modified Arg^31^) or ribose, ADPr or ADPr-A′ adducts, revealed that the RNA is attached by an N-glycosidic bond to R139 and/or R142 (Fig. 3c–e, Extended Data Fig. 5 and Supplementary Table 3). We could not unambiguously assign the modified Arg because of the low intensity of the respective fragment ions and the occurrence of mixed spectra containing ion fragments of the same peptide species modified at different sites (Fig. 3c–e).

## rS1 is RNAylated and ADP-ribosylated in vivo

**Fig. 4:**
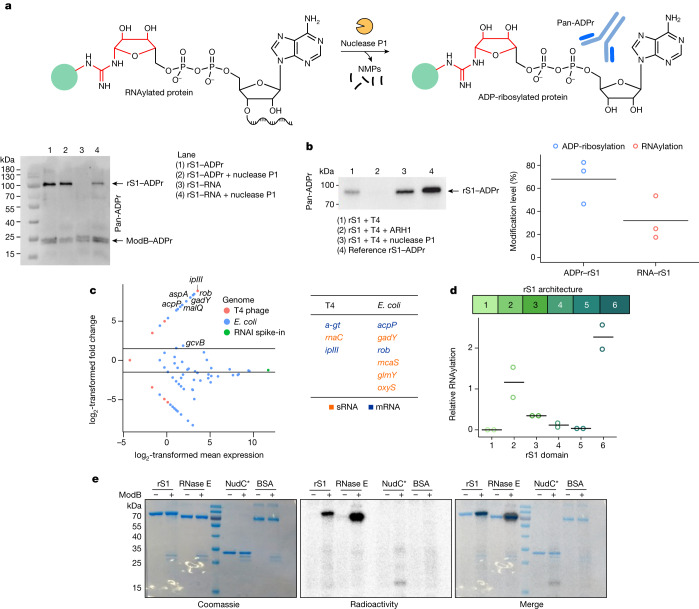
In vivo characterization of ADP-ribosylation and RNAylation. **a**, Quantification of the RNAylation of rS1 using a nuclease P1 digest and western blot analysis. Green circle represents the protein. **b**, Quantification of rS1 RNAylation in vivo based on biological triplicates (*n* = 3). Data are shown as mean (grey bar) and individual data points. Complete blots and intensity normalization are shown in Extended Data Fig. 6b. **c**, Identification of RNA substrates of ModB using RNAylomeSeq. The MA plot shows data for one of three biological replicates (*n* = 3). Further details are given in Extended Data Fig. 6c,d. **d**, Quantification of the RNAylation of rS1. Modification of rS1 domains 1–6 (*n* = 2 biologically independent replicates; black lines show the mean). **e**, SDS–PAGE analysis of the RNAylation of protein rS1, RNase E, inactive NudC mutant (NudC*: V157A, E174A, E177A, E178A) and bovine serum albumin (BSA) by ModB (*n* = 2 biologically independent replicates). Source data

**Extended Data Fig. 6:**
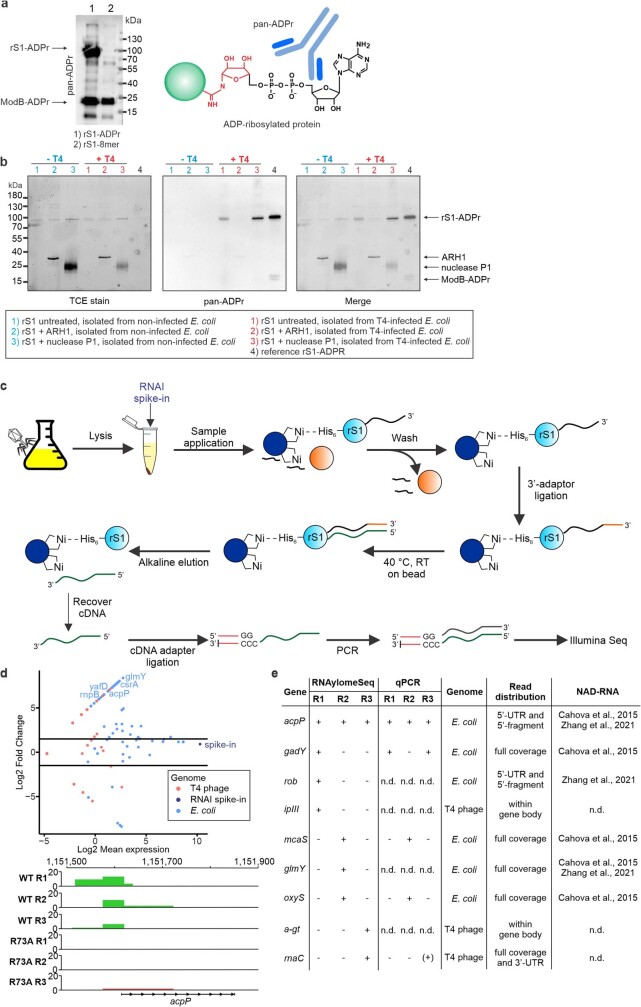
*In vivo* characterisation of the RNAylation by Western blot and RNAylomeSeq. **a**, Analysis of the substrate specificity of the pan-ADPr antibody. *In vitro* prepared ADP-ribosylated or RNAylated protein rS1 was applied to evaluate the specificity of the antibody (n = 3). The pan-ADPr antibody detected ADP-ribosylated proteins rS1 and ModB (lane 1). In contrast, RNAylated rS1 is not detected by pan-ADPr (lane 2). However, a signal for ADP-ribosylated ModB was observed due to self-ADP-ribosylation in its expression host *E. coli* (lane 2). **b**, Quantification of RNAylation using the combination of nuclease P1 digest and detection of protein-linked ADP-ribose by Western blot. Visualisation of protein load by TCE stain. Removal of the ADP-ribose signal by ARH1 treatment. pan-ADPr signals for ADP-ribosylated rS1 were normalised to corresponding band intensities in the TCE stain. Normalised intensities for untreated rS1 were then divided by the intensity for P1-treated rS1 to yield the fractions of ADP-ribosylated and RNAylated rS1 among the two modifications. The corresponding dot plot is shown in Fig. 4b (n = 3 biologically independent replicates). **c**, Schematic illustration of the RNAylomeSeq protocol: Identification of RNAylated RNAs which are covalently attached to rS1 *in vivo*. Briefly, endogenously His-tagged rS1 is isolated from T4 phage infected *E. coli* with Ni-NTA beads. A spike-in - rS1 domain 2 RNAylated with NAD-RNAI - (RNAI spike-in) is added to the lysate which is meant to be enriched via the RNAylomeSeq workflow. rS1 captured on Ni-NTA beads is intensively washed with 8 M urea in order to remove RNA non-covalently bound to rS1. Similar to NAD captureSeq^32^, an RNA 3′-adapter is ligated to covalently linked RNAs and RNA is reverse transcribed “on-bead”. cDNA is then eluted by alkaline digest of RNA and an additional adapter is ligated to the 3′-terminus of the cDNA. cDNA is amplified by PCR and sequenced by next-generation single-end sequencing (Illumina). Importantly, the RNAI spike-in is not meant to be enriched in any sample but rather to be found in each sample in similar amounts. Thereby, read counts can be normalised to the RNAI counts in each sample allowing for their comparison. **d**, MA plot showing RNAs enriched in the T4 phage WT infected sample compared to T4 phage ModB R73A, G74A control identified by RNAylomeSeq for replicate 2 (total of n = 3 biological replicates). Read counts per sample have been normalised to RNAI spike-in read counts which serves as an enrichment control for each sample. Thus, RNAI is not found enriched comparing T4 WT and T4 ModB R73A, G74A. Mean expression values (T4 WT and T4 ModB R73A, G74A condition) have been normalised by Log2 (x-axis) for each replicate separately. T4 WT and T4 ModB R73A, G74A read counts were compared via log2 fold change (y-axis). Read coverage on identified RNAylated RNAs as analysed in IGV is exemplarily shown for *acpP* in the lower panel depicting reads in T4 WT samples (green) vs. T4 ModB R73A, G74A samples (red). RNAylomeSeq merely identifies 5’-termini of mRNAs or, if 200nt or smaller, entire sRNA sequences. This is due to the application of single-end Illumina-Seq which automatically only captures the 5’-end of the respective read/transcript. **e**, Selected hits of RNAs identified by RNAylomeSeq comparing T4 phage WT and T4 ModB R73A, G74A. *acpP* was identified in all three replicates. However, some transcripts were only detected in one or two replicates. Enrichments have been further validated on cDNA level by qPCR. +: enriched; −: not enriched; (+): enriched, but Log2 fold change <= 1; n.d.: not defined.

To distinguish quantitatively between ADP-ribosylation and RNAylation in vivo, we used immunoblotting with an antibody-like ADPr-binding reagent (pan-ADPr) that specifically recognizes ADP-ribosylated proteins but detects RNAylated proteins only after treatment with nuclease P1 (Fig. 4a and Extended Data Fig. 6a). rS1 was expressed in non-infected or T4-infected *E.* *coli*, affinity-purified and its ADP-ribosylation was analysed with pan-ADPr. We found extensive ADP-ribosylation of rS1 only in the T4-infected sample. After treatment with nuclease P1, the pan-ADPr signal intensity of the rS1 band increased (Fig. 4b and Extended Data Fig. 6b), indicating RNAylation of rS1. Thus rS1 was found to be both ADP-ribosylated and RNAylated in vivo, with RNAylation accounting for around 30% of the modifications. It remained unclear, however, whether the two modifications are mutually exclusive or can occur simultaneously in the same molecule at different sites. Moreover, the signal for ADPr disappeared after ARH1 treatment, further confirming the nature of the RNA–protein linkage (Fig. 4b and Extended Data Fig. 6b). We found that the ADP-ribosylation and RNAylation of rS1 occur in parallel in vivo.

## ModB RNAylates proteins with selected RNAs

To identify the RNAs linked to rS1 by ModB during infection by the T4 phage, we developed an RNAylomeSeq approach (Extended Data Fig. 6c) in which genomically His-tagged rS1 was isolated from T4-infected *E.* *coli* and captured on Ni-NTA beads. In a similar way to NAD captureSeq^32^, RNA was reverse-transcribed ‘on-bead’ and the resulting cDNA was amplified by PCR and analysed using next-generation sequencing.

We applied this workflow to *E.* *coli* treated with wild-type (WT) T4 phage. As a negative control, we used CRISPR–Cas9 technology to generate a T4 phage that expressed the catalytically inactive mutant ModB(R73A, G74A) (ref. ^33^). We compared the abundance of reads mapped to individual RNA species and identified specific *E.* *coli* and T4 phage RNAs enriched in WT T4 phage samples (Fig. 4c, Extended Data Fig. 6d,e, Supplementary Table 4 and Supplementary Fig. 3). Several of the *E.* *coli* transcripts (mRNAs and sRNAs) have been reported to be 5′-NAD-capped in *E.* *coli*^3,34^, including RNAs of the genes *acpP*, *glmY*, *mcaS*, *oxyS*, *aspA* and *rob*, which makes them suitable substrates for ModB. We also identified phage transcripts, such as *ipIII* (internal head protein III), that were enriched in our datasets (Fig. 4c, Extended Data Fig. 6d,e and Supplementary Table 4). The enriched RNAs do not share any common features apart from adenosine (+1A) at the transcription start site, which is crucial for the biosynthesis of NAD-capped RNAs in vivo^35^.

## ModB RNAylates OB-fold proteins

**Extended Data Fig. 7:**
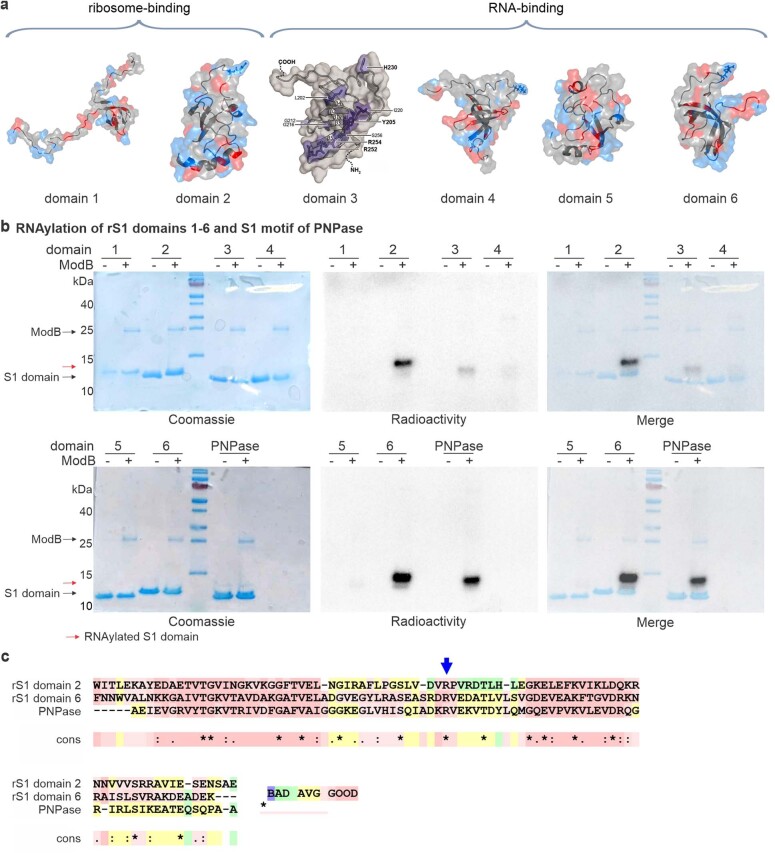
RNAylation of rS1 domains D1 – D6 and S1 motif of PNPase by ModB *in vitro*. **a**, Illustration of the rS1-motifs of rS1 based on crystal structures (PDB) of domains 1 (2MFI), 2 (2MFL), 4 (2KHI), 5 (5XQ5) and 6 (2KHJ) as well as an NMR structure of domain 3^64^. **b**, *in vitro* RNAylation of S1 domains and PNPase by ModB using a ^32^P-NAD-8mer. ModB and S1 domains (D1-6) are marked with black arrows. RNAylated rS1 domains, characterised by a shift compared to the non-modified proteins, are highlighted with red arrows. n = 2 of biologically independent replicates. Reactions were analysed by 16 % Tricine-SDS-PAGE, stained in Coomassie and RNAylation recorded by autoradiography imaging (Radioactivity). **c**, Local alignment of rS1 D2 and D6 as well as the S1 domain of PNPase using T-coffee expresso^65^. R139 of D2 (highlighted with an arrow) is conserved in PNPase and D6.

**Extended Data Fig. 8:**
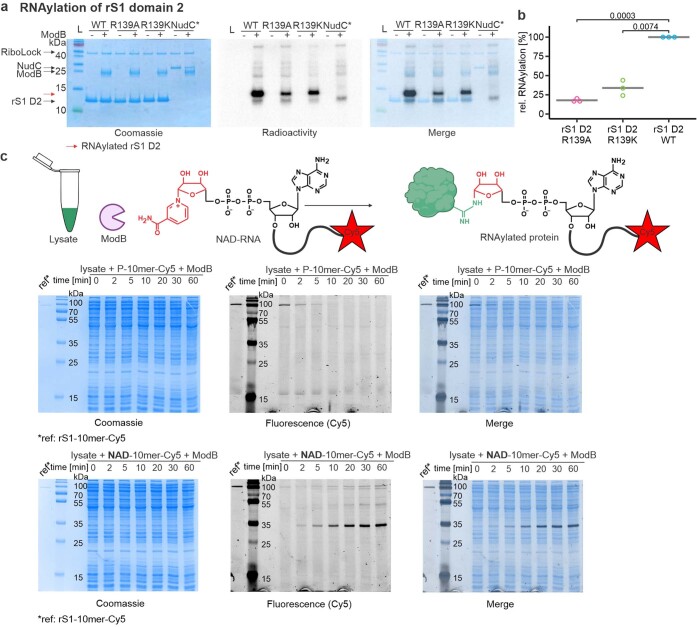
Characterisation and identification of RNAylation target proteins of ModB. **a**, Analysis of the *in vitro* RNAylation of rS1 domain 2 and its mutants R139A and R139K by 16 % Tricine-SDS-PAGE. An inactive NudC mutant (NudC*; V157A, E174A, E177A, E178A) was used as a negative control (n = 3). Radioactivity indicates RNAylation, Coomassie scan visualises protein load. **b**, Quantification of relative intensities of RNAylation of rS1 domain 2 and its mutants R139A and R139K based on radioactivity in 16 % Tricine-SDS-PAGE analysis. Per replicate, intensities were normalised to the rS1 D2 WT band intensity. A two-sided t-test was performed at p_signif_. < 0.05 indicating significantly decreased RNAylation of R139 mutants of rS1 domain 2 (p-value = 0.0003 (WT vs. R139A) and 0.0074 (WT vs. R139K)). n = 3 of biologically independent replicates. **c**, RNAylation of *E. coli* cell lysate by ModB using 3′-Cy5-labelled NAD-RNA (schematically shown in upper panel). A time course of *E. coli* cell lysate RNAylation by ModB in the presence of either a 5′-monophosphorylated RNA 10mer (P-10mer-Cy5, middle panel) or 5′-NAD-capped RNA 10mer (NAD-10mer-Cy5, lower panel), each with a 3′-fluorescent (Cy5) label. rS1 RNAylated with an NAD-10mer-Cy5 is applied as a reference (ref). The time course of lysate RNAylation was analysed by 12 % SDS-PAGE, protein visualised by Coomassie staining and RNAylation recorded via fluorescence (Cy5). n = 3 of biologically independent replicates. NAD concentration in the lysates exceeds the utilised NAD-10mer concentration by 48-fold. NAD concentration in the lysates of 22.5 µM (n = 1 biologically independent replicates, n = 3 technical replicates) was determined using the NAD/NADH-Glo assay (Promega). The schematic protein and tube in c were created using BioRender (https://biorender.com). Source data

To understand how ModB identifies its target proteins, we analysed the structural features of known target proteins. rS1 contains oligonucleotide-binding (OB)-fold domains^28^. One structural variant of OB folds is the S1 domain, which is present in rS1 in six copies that vary in sequence (Extended Data Fig. 7a). RNAylated R139 and R142 are located in domain 2 of rS1. We speculated that the S1 domain might be important for substrate recognition by ModB. To characterize the specificity of ModB for different S1 domains, we cloned, expressed and purified each S1 domain of rS1 (D1–D6) and tested them in an RNAylation assay (Fig. 4d and Extended Data Fig. 7b). In agreement with the mass spectrometry (MS) data (Extended Data Table 1 and Supplementary Table 1), we detected strong RNAylation signals for rS1 D2 and D6, whereas rS1 D1, D3, D4 and D5 were modified to a lesser extent. Multiple sequence alignment of rS1 D2 and D6, and the S1 domain of *E.* *coli* PNPase, revealed that these S1 domains share an Arg residue as part of the loop that connects strands 3 and 4 of the β-barrel^36^ (Extended Data Fig. 7c). This loop is packed on the top of the β-barrel and might therefore be accessible to ModB. For rS1 D2, the residues R139 and R142 are the sites of RNAylation identified by MS (Fig. 3e–g and Supplementary Tables 1–3). Mutation analysis confirmed that the RNAylation level of D2 is significantly reduced if R139 is replaced by Ala or Lys (Extended Data Fig. 8a,b). *E.* *coli* RNase E also has an S1 domain in its active site with an Arg in the loop between strands 3 and 4. In the RNAylation in vitro assays, RNase E was modified by ModB, whereas control proteins without the S1 domain (such as BSA and the NudC inactive mutant) were not. These data suggest that OB folds such as S1 domains with an embedded Arg are RNAylation target motifs (Fig. 4e).

## rL2 is a target for RNAylation by ModB

**Fig. 5:**
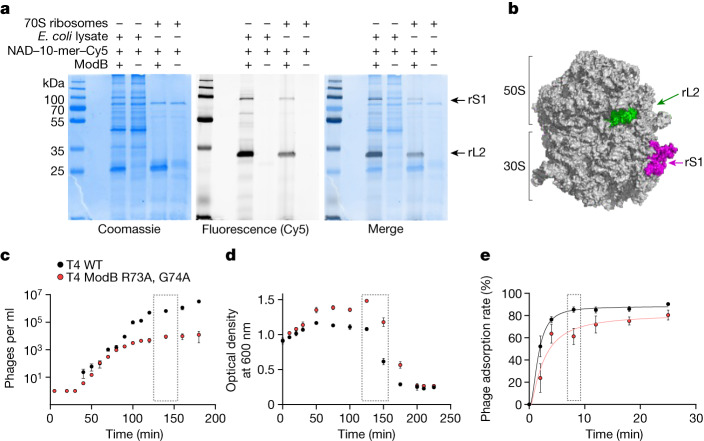
RNAylation of the ribosome and phenotype of a ModB mutant T4 phage. **a**, Characterization of ModB substrate specificity. RNAylation of two ribosomal proteins (rS1 and rL2) in cell lysates and 70S ribosome assemblies (*n* = 3). **b**, Illustration of the RNAylated proteins rS1 and rL2 in the context of the 70S ribosome, based on the cryo-electron microscopy structure of the hibernating 70S *E.* *coli* ribosome (PDB: 6H4N)^51^. **c**–**e**, Characterization of the T4 ModB R73A, G74A mutant phenotype, showing the burst size (**c**), *E.* *coli* lysis (**d**) and phage adsorption (**e**) of WT T4 phages and T4 ModB(R73A, G74A) (*n* = 3 biologically independent replicates for each). Data points with error bars represent mean ± s.d. Grey dotted boxes indicate time points used for assessing statistical significance in the case of burst size (**c**, 140 min after infection; two-sided Student’s *t*-test, *P* = 0.0015 at *P*_signif_ < 0.05) and phage adsorption (**e**, 8 min after infection; *t*-test, two-sided, *P* = 0.029 at *P*_signif_ < 0.05) but indicate the delayed lysis without a statistical test in **d**. Statistical tests are shown in Supplementary Fig. 4. Source data

To discover additional RNAylation target proteins of ModB, a cell lysate, prepared from exponentially growing *E.* *coli*, was incubated with purified ModB and an NAD–10-mer RNA with a fluorescent 3′ Cy5 label (Fig. 5a and Extended Data Fig. 8c). We approximated the cellular conditions with respect to the presence of proteins, nucleic acids and various small molecules, including NAD^37^.

**Extended Data Fig. 9:**
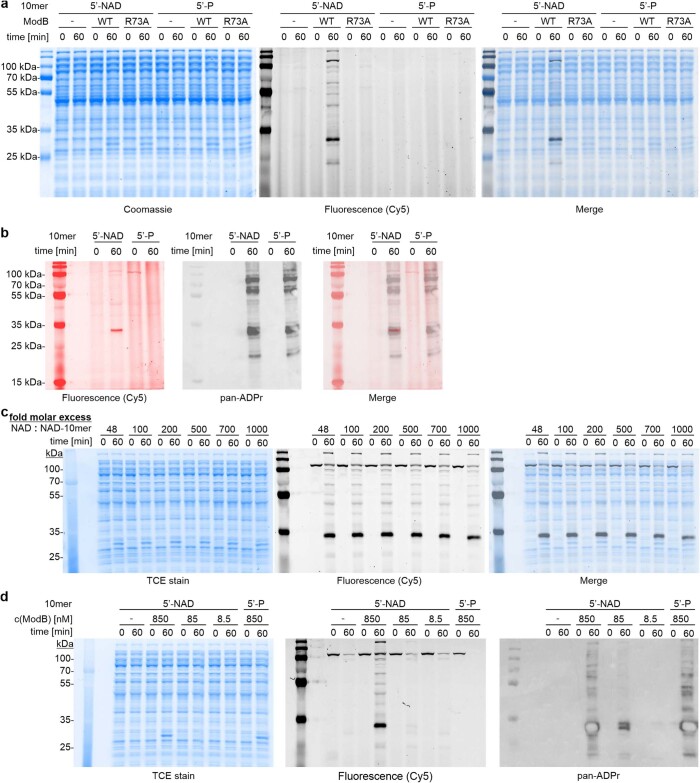
Characterisation of the specificity of ModB-mediated RNAylation in *E. coli* lysates. **a**, RNAylation of *E. coli* cell lysate in the presence of ModB WT or inactive ModB R73A, G73A or the absence of ModB using 3′-Cy5-labelled NAD-10mer or P-10mer. Time point 0 shows lysate before addition of ModB, 60 min shows RNAylation after 60 min incubation with ModB. Reactions were analysed by 12 % SDS-PAGE, protein visualised by Coomassie staining and RNAylation recorded via fluorescence (Cy5). n = 2 of biologically independent replicates. **b**, Samples from lysate RNAylation with Cy5-labelled 5′-NAD- or 5′-P-10mer (as presented in Extended Data Fig. 8c) before addition of ModB (0 min) and after 60 min incubation in the presence of ModB (60 min) were analysed by 10 % SDS-PAGE and RNAylation monitored by fluorescence (Cy5, here shown in red). Subsequently, Western blotting was performed and ADP-ribosylation was detected using pan-ADPr binding reagent (MABE1016, shown in grayscale). n = 2 of biologically independent replicates. Different band patterns were observed for ModB-mediated RNAylation and ADP-ribosylation in *E. coli* lysates indicating a distinct target specificity of ModB for RNAylation. NAD concentration in the lysates exceeds utilised NAD-10mer concentration by 48-fold. NAD concentration in the lysates of 22.5 µM (n = 1 biologically independent replicates, n = 3 technical replicates) was determined using the NAD/NADH-Glo assay (Promega). **c**, Lysate RNAylation by ModB in the presence of various molar excesses of NAD over NAD-10mer-Cy5 ranging from 48-fold (native lysate) to 1000-fold via additional spike-in NAD (n = 2). Cy5 represents RNAylation. TCE stain indicates protein load, which is enabled by binding of trichloroethanol in the gel to tryptophan residues in proteins which enhances their fluorescence under UV light and thereby enables their detection^53^. 700-fold molar excess of NAD reduces RNAylation to 67 % (n = 2 biologically independent replicates) compared to “native” lysate. Total Cy5 signals for each lane were quantified to determine and compare RNAylation levels. **d**, Lysate RNAylation and ADP-ribosylation in the presence of various ModB concentrations (850, 85 and 8.5 nM) monitored by fluorescence (Cy5 for RNAylation) and Western blot (pan-ADPr for ADP-ribosylation). TCE stain indicates protein load. In average, RNAylation is reduced to 8.6 % and ADP-ribosylation is reduced to 6.9 % in lysates with ModB concentrations that approximate the cellular conditions (85 nM). Total Cy5 or pan-ADPr signals (excluding ModB ADP-ribosylation signal) for each lane were quantified to determine and compare RNAylation or ADP-ribosylation levels, respectively. n = 2 biologically independent replicates.

Kinetic analysis of the ModB activity in these lysates showed that several *E.* *coli* proteins were RNAylated (Extended Data Figs. 8c and 9a), including rS1 (which migrates in a similar way to an RNAylated rS1 we added as a marker) and a protein with a mass of around 35 kDa. Notably, this pattern was not observed in the presence of 5′-monophosphorylated RNA–Cy5. We also characterized the simultaneous ADP-ribosylation in the same lysates showing different patterns of ADP-ribosylation targets and RNAylation targets of ModB (Extended Data Fig. 9b). In *E.* *coli*, NAD–RNA concentrations amount to around 5 µM (ref. ^4^), compared with an approximately 700-fold excess of NAD (2.6 mM; ref. ^37^). To simulate this molar excess of NAD over NAD–RNA in the lysate assay, we added NAD to our lysates. This showed that with a 700-fold excess of NAD, RNAylation still occurs with an efficiency of approximately 67% (Extended Data Fig. 9c). We then assessed the intensity of ModB relative to *E.* *coli* proteins by proteomics, which revealed that a 100-fold dilution, relative to our standard assay conditions, may resemble relative ModB intensity during infection^38^ (Extended Data Table 3). In lysates with ModB concentrations closer to those in cellular conditions, similar ADP-ribosylation and RNAylation patterns were observed as under standard conditions (Extended Data Fig. 9d).

These results indicate that in cellular conditions in which NAD is much more abundant than NAD–RNA, ModB RNAylates specific target proteins (Extended Data Figs. 8c and 9c). Because ModB was previously assumed to preferentially ADP-ribosylate proteins involved in translation^1^, we monitored the RNAylation patterns of isolated *E.* *coli* ribosomes (Fig. 5a) and observed a similar pattern to that for the lysates (Extended Data Figs. 8c and 9).

**Extended Data Fig. 10:**
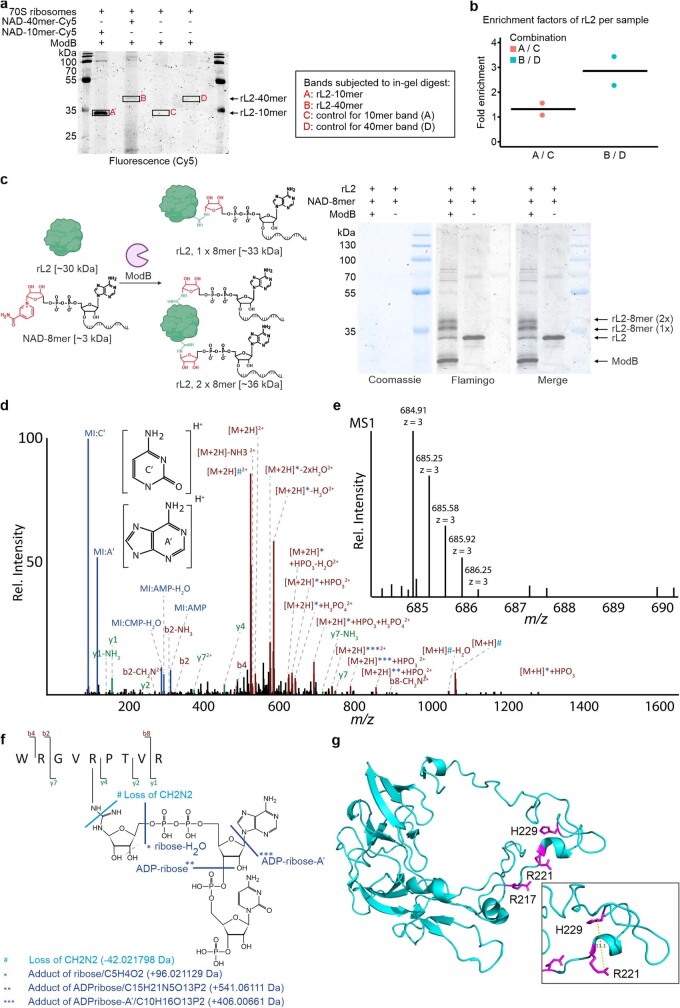
Scope of ModB RNAylation targets in *E. coli*. **a**, RNAylation of *E. coli* ribosomes by ModB. RNAylated protein is shifted upon incubation with NAD-40mer compared to NAD-10mer which itself increases protein weight by approx. 3 kDa. Relative enrichment of RNAylated target protein was assessed by subjecting RNAylated protein bands and respective control bands generated in the absence of RNA to in-gel digest and LC-MS/MS analysis (n = 2). **b**, Plot of the enrichment of fractional spectral counts for 50S ribosomal protein L2 (rL2) based on in-gel-digest and LC-MS/MS analysis presented in a. Enrichment is calculated for RNAylation with NAD-10mer (A/C) or NAD-40mer (B/D), relative to the respective, non-RNAylated control bands based on spectral counts from Scaffold (n = 2). **c**, Analysis of the *in vitro* RNAylation of rL2 by ModB in the presence of NAD-8mer. RNAylated rL2 proteins have reduced electrophoretic mobility during SDS-PAGE. Protein was visualised by fluorescent protein stain (Flamingo) and protein ladder visualised by Coomassie staining. Signals were quantified using ImageLab indicating that about 80 % of rL2 is RNAylated by ModB *in vitro* (n = 3). Band patterns indicated that rL2 can be RNAylated once or even twice *in vitro*. **d**–**f**, Tandem MS-based identification of RNAylated rL2 peptide. **d**, MS/MS fragment ion spectrum (spectrum ID: 8679) of RNAylated rL2 peptide WRGVRPTVR carrying ADP-ribose plus cytidine-monophosphate and a 3′-phosphate group. The spectrum shows marker ions of adenine (A’) and cytosine (C’) as well as AMP and CMP. The precursor ion ([M+xH]^x+^) is detected unshifted, shifted by the mass of ADP-ribose (*) and by ADP-ribose with adenine loss (**). Also, precursor ions show a specific loss of 42.021798 Da, which can be explained by a loss of CH_2_N_2_ at the modified arginine. **e**, Isotopic peak pattern of the precursor ion shown in **d**, as detected in the corresponding MS precursor ion scan. **f**, Schematic sequence and RNA adduct representation of the RNAylated peptide shown in **d** and **e** including annotations of fragment ions. The fragmentation products observed in the MS/MS spectrum, shown in **d**, of the ADP-ribose+CMP+3′-phosphate adduct are indicated in the structure by light blue (mass loss) and dark blue (mass adducts) lines. **g**, Selected RNAylated residues of rL2 identified by LC-MS/MS. The catalytically important H229 is 11.1 Å apart from R221. rL2 structure derived from a 1.98 Å cryo-EM structure (7K00)^66^. The schematic protein in c was created using BioRender (https://biorender.com). Source data

To identify the RNAylated proteins, we RNAylated the *E.* *coli* ribosome with a 40-nucleotide-long NAD–RNA, resulting in a gel shift of RNAylated ribosomal proteins. MS analysis of the isolated gel band identified the ribosomal protein L2 (rL2) as a target for RNAylation by ModB (Extended Data Fig. 10a,b). rL2 is a protein with a mass of around 35 kDa and is probably the target observed in the lysates (Extended Data Figs. 8c and 9). It is evolutionarily highly conserved and is required for the association of the 30S and 50S subunits, involved in tRNA binding to both the A and P sites, and important for peptidyltransferase activity^39^. Similar to rS1, PNPase and RNase E, rL2 contains an RNA-binding domain that is homologous to the OB fold^40^. In vitro RNAylation assays found that about 80% of the rL2 was RNAylated by ModB in the presence of NAD–RNA (Extended Data Fig. 10c). In vitro RNAylation sites of rL2 were identified using the LC–MS/MS approach, including an MS data search with RNPxl, as described above. Trinucleotides (ADPr–C) were found to be attached to R217 and R221 (Extended Data Fig. 10d–g and Supplementary Table 6). R221 is located close (11 Å away) to H229, which is indispensable for ribosomal peptidyltransferase activity^39^. Future studies will reveal whether the RNAylation of rL2 and rS1 influences the translation efficiency of the ribosome (Fig. 5b).

## ModB is important for phage infection

To investigate the functional role of ModB during phage infection, we compared the phenotypes of WT T4 and T4 ModB(R73A, G74A). We observed that the burst size (the number of virions released per infected *E.* *coli* cell) of T4 ModB(R73A, G74A) was decreased fourfold by 50 min after infection (15 ± 3 progeny per cell) compared with WT T4 (60 ± 32 progeny per cell) (Fig. 5c). By 140 min after infection, phages produced by WT T4 (6.6 × 10^5^ ± 1.3 × 10^5^ progeny) significantly exceeded the number of progeny from T4 ModB(R73A, G74A) (5.5 × 10^4^ ± 3.1 × 10^4^) (Fig. 5c and Supplementary Fig. 4a). At 140 min after infection, a 12-fold decrease in the progeny number compared with the WT T4 phage was observed for T4 ModB(R73A, G74A). Thus, ModB inactivation noticeably affects phage propagation properties.

We also observed a delay in lysis of approximately 20 min for the *E.* *coli* culture grown in the presence of the mutant phages (Fig. 5d). To determine whether ModB affects the infection cycle at the intra- or extracellular stage of infection, we measured the kinetics of phage adsorption to the cell (Fig. 5e). We observed a significantly lower adsorption rate for mutant phages. At 8 min after infection, around 61.3 ± 7.3% of the T4 ModB(R73A, G74A) mutants successfully entered *E.* *coli*, compared with 85.3 ± 2.4% for WT T4 phages (Fig. 5e and Supplementary Fig. 4b). These results indicate that phages are generated in the presence of inactivated ModB are less effective in the first stages of the infection, namely the attachment to, and penetration of, the host. This finding is consistent with the delayed host lysis.

## Discussion

Most of the interactions between RNA and proteins are non-covalent^41^, but there are some exceptions^42^. These include the peptidyl–tRNA intermediates in protein biosynthesis^43^ (which are esters) and the adenoviral VPg proteins that form a phosphodiester bond (by means of a tyrosine OH group) with a nucleotide, which is then used to initiate transcription^44,45^. Here we show that an ART can attach NAD-capped RNAs to target proteins post-transcriptionally through the formation of glycosidic bonds. This finding represents a distinct biological function of the NAD cap on RNAs in bacteria, namely the activation of the RNA for enzymatic transfer to an acceptor protein. We discovered that the RNAylation of target proteins (a previously undescribed post-translational protein modification) has a role in the infection of the bacterium *E.* *coli* by bacteriophage T4. We discovered that ModB is a target-specific ART that RNAylates proteins that are part of the translational apparatus. We found that rS1 and rL2 are RNAylated at specific Arg residues in their RNA-binding regions. Moreover, we identified predominantly *E.* *coli* transcripts that are linked to rS1 during T4 phage infection. Inactivation of ModB caused a delay in bacterial lysis during phage infection and decreased the number of progeny released. It remains unclear how the mutation of ModB (a non-capsid protein) will affect phage adsorption to the host cell. Precisely defining phage composition and architecture in future studies might help to explain this phenomenon.

Our findings introduce a molecular mechanism by which the T4 phage targets the translational machinery of its host and indicate that RNAylation might have a role in bacteriophage pathogenicity. It remains to be determined, however, whether ADP-ribosylation or RNAylation is the more important function of ModB. The T4 mutant ModB(R73A, G74A) abolished not only RNAylation but also ADP-ribosylation activity. This makes it difficult to determine whether the observed effects on T4 infection are due to RNAylation specifically or to the loss of ADP-ribosylation activity.

ModB was known to be an enzyme that uses NAD as a substrate to ADP-ribosylate host proteins during T4 infection. During this study, it became clear that ModB accepts not only NAD as a substrate, but also NAD–RNA. Enzymes typically have high specificity for their substrates and tolerate only limited chemical modifications. It was therefore surprising that ModB tolerates the attachment of a bulky RNA chain to the 3′ OH group of NAD (NAD–RNA) for the modification of a specific subset of target proteins. Remarkably, all four of the proteins (rS1, rL2, RNase E and PNPase) identified here as RNAylation targets of ModB are well known to interact with RNA. We therefore assume that both the ability of ModB to accept NAD–RNA as a substrate and the RNA affinity of the target protein determine RNAylation specificity. We did not succeed in generating a mutant of ModB that only ADP-ribosylates or RNAylates. RNAylation occurs by an ADP-ribosylation-like mechanism that involves the same catalytic residues as ADP-ribosylation, but the RNA affinity of the target protein might determine RNAylation specificity.

We considered why a phage ART would attach specific RNAs to proteins involved in translation. When a T4 phage infects *E.* *coli* it aims to reprogram the host ribosome to translate its mRNAs^46^. One way to achieve this may be a controlled shutdown of ribosomes that do not participate in the translation of T4 mRNAs. The discovery of crucial ribosomal proteins, rS1 and rL2, as RNAylation targets leads us speculate that RNAylation might impair their functionality, such as modulating peptidyltransferase activity. The fact that mostly *E.* *coli* transcripts are linked to rS1 in vivo suggests that undesired host gene-expression events are stopped by RNAylation. In this way, the phage might exploit RNAylation to inactivate distinct host ribosomes.

Future studies could show whether ribosomes that translate *E.* *coli* transcripts are blocked by RNAylation. This proposed mechanism would enable the phage to regulate the activity of the ribosome throughout the infection cycle and to stop the translation of host proteins.

Why only one of the three known T4 ARTs carries out efficient RNAylation is not understood. ModA and ModB both contain characteristic features of Arg-specific ARTs, such as the active-site motif R-S-EXE^1^. Differences in substrate specificity are therefore probably due to sequence differences (ModA and ModB are 25% identical and have 47% homologous amino acids)^1^.

ARTs are not limited to phages. ADP-ribosylated proteins have been detected in hosts following infection by various viruses, including influenza, coronaviruses and HIV. As well as viruses using ARTs as weapons, the mammalian antiviral defence system uses host ARTs to inactivate viral proteins. Moreover, mammalian ARTs and poly-(ADPr) polymerases are regulators of critical cellular pathways and are known to interact with RNA^47^. Thus ARTs might catalyse RNAylation reactions in different organisms, making RNAylation a phenomenon of broad biological relevance.

Finally, RNAylation may be considered as both a post-translational protein modification and a post-transcriptional RNA modification. Our findings challenge the established views of how RNAs and proteins interact with each other. The discovery of these previously undescribed RNA–protein conjugates comes at a time when the structural and functional boundaries between different classes of biopolymer are becoming increasingly blurred^48,49^.

## Methods

### General

Reagents were purchased from Sigma-Aldrich and used without further purification. Oligonucleotides, DNA and RNA were purchased from Integrated DNA Technologies (Supplementary Tables 7–10). Concentrations of DNA and RNA were determined by measurements using the NanoDrop ND-1000 spectrophotometer. Radioactively labelled proteins and nucleic acids were visualized using storage phosphor screens (GE Healthcare) and a Typhoon 9400 imager (GE Healthcare). Uncropped gel and blot images are provided (Supplementary Fig. 1).

### Preparation of 5′ppp–RNA, 5′p–RNA and 5′-NAD–RNA by in vitro transcription

DNA templates for Qβ RNA (100-nucleotide RNA) and *E.* *coli* RNAI were amplified by PCR (primer sequences are listed in Supplementary Table 9), and PCR products were analysed by 2% agarose gel electrophoresis and purified using the QIAquick PCR purification kit (QIAGEN). 5′-Triphosphate (ppp) Qβ RNA and RNAI were synthesized by in vitro transcription in the presence of 1× transcription buffer (40 mM Tris, pH 8.1, 1 mM spermidine, 10 mM MgCl_2_, 0.01% Triton X-100), 5% DMSO, 10 mM DTT, 4 mM of each NTP, 20 μg T7 RNA polymerase (2 mg ml^−1^, purified in our laboratory) and 200 nM DNA template. NAD–RNAI was made under similar conditions using 2 mM ATP and 4 mM NAD. The same conditions were applied for the synthesis of a mixture of α-^32^P-labelled 5′-NAD and pppQβ RNAs, except we used 2 mM ATP, 80 μCi ^32^P-α-ATP and 4 mM NAD instead of 4 mM ATP. The in vitro transcription reactions were incubated at 37 °C for 4 h and digested with DNase I (Roche). RNA was purified by denaturing PAGE, isopropanol-precipitated and resuspended in Millipore water. RNA sequences are listed in Supplementary Table 7.

To convert 5′ppp–RNAs into 5′-monophosphate–RNAs (5′p–RNAs), 250 pmol Qβ RNA was treated with 60 U RNA 5′-polyphosphatase (Epicentre) in 1× polyphosphatase reaction buffer at 37 °C for 70 min. Protein was removed from 5′p–RNAs by phenol–chloroform extraction and residual phenol–chloroform was removed by three rounds of diethyl ether extraction. 5′p–RNAs were isopropanol precipitated and resuspended in Millipore water.

### 5′-radiolabelling of 5′-monophosphate and NAD-capped RNAs

We treated 120 pmol 5′p-Qβ RNA or 6.25 nmol 5′p–RNA 8-mer (Supplementary Table 7) with 50 U T4 polynucleotide kinase in 1× reaction buffer B and 1,250 μCi ^32^P-γ-ATP. The reaction was incubated at 37 °C for 2 h. The resulting 5′-^32^P-RNA 8-mer and 5′-^32^P-Qβ RNA were separated from residual protein by phenol–chloroform extraction. The remaining ^32^P-γ-ATP was removed by washing with three column volumes of Millipore water and centrifugation in 10 kDa (for Qβ RNA) or 3 kDa (for the 8-mer) Amicon filters (Merck Millipore) at 14,000 rpm at 4 °C four times. RNA sequences are listed in Supplementary Table 7. To convert the purified 5′-^32^P-RNAs into 5′-^32^P-NAD-capped RNA, 800 pmol 5′-^32^P-RNA 8-mer or 30 pmol 5′-^32^P-Qβ RNA was incubated in 50 mM MgCl_2_ in the presence of a spatula tip of nicotinamide mononucleotide phosphorimidazolide, synthesized as described^52^, at 50 °C for 2 h. RNAs were purified by washing with Millipore water and centrifugation in 10 kDa (Qβ RNAs) or 3 kDa (8-mer) Amicon filters at 14,000 rpm at 4 °C four times. The concentrations of the 5′-^32^P-RNAs were measured using a NanoDrop ND-1000 spectrophotometer and were used to calculate the approximate concentrations of yielded 5′-NAD-capped ^32^P-RNAs, assuming an approximate yield of the imidazolide reaction of 50% (ref. ^52^). The 5′-^32^P-ADPr–RNA 8-mer was synthesized by incubating 8 µM 5′-^32^P-NAD–RNA 8-mer and 0.08 µM ADP-ribosyl cyclase CD38 (R&D Systems) in 1× degradation buffer at 37 °C for 4 h. The reaction was purified by P/C/I-diethyl ether extraction and filtration through 3 kDa filters and washing with four column volumes of Millipore water.

### Cloning of ADP-ribosyltransferases, ADP-ribose hydrolases and target proteins

To amplify bacteriophage T4 genes *modA* (GeneID: 1258568; Uniprot: P39421), *modB* (GeneID: 1258688; Uniprot: P39423) and *alt* (GeneID: 1258760; Uniprot: P12726), a single plaque from bacteriophage T4 revitalization was resuspended in Millipore water and used in a ‘plaque’ PCR, analogous to bacterial-colony PCR. The gene encoding the ADP-ribosylhydrolase ARH1 (GeneID: 141; Uniprot: P54922) was purchased from IDT as gBlocks and amplified by PCR. *E.* *coli* genes coding for rS1 (GeneID: 75205313; Uniprot: P0AG67), rL2 (GeneID: 947820; Uniprot: P60422) and PNPase (GeneID: 947672; Uniprot: P05055) were PCR-amplified from genomic DNA of *E.* *coli* K12, which was isolated using a GenElute Bacterial Genomic DNA Kit (Sigma-Aldrich). Nucleotide sequences are listed in Supplementary Table 8. XhoI and NcoI restriction sites were introduced during amplification using appropriate primers (Supplementary Table 9). The resulting PCR product was digested with XhoI and NcoI (Thermo Fisher Scientific) and cloned into the pET–28c vector (Merck Millipore). After Sanger sequencing, the resulting plasmids were transformed into *E*. *coli* One Shot BL21 (DE3) (Life Technologies). The ARH1 D55,56A, ModB(R73A) and rS1 mutants were generated by site-directed mutagenesis using a procedure based on the Phusion Site-Directed Mutagenesis Kit (Thermo Scientific). The resulting plasmids were sequenced and transformed into *E*. *coli* One Shot BL21 (DE3). All strains used and generated in this work are summarized in Supplementary Table 10.

### Purification of rS1, rS1 domains and variants, rL2, the PNPase S1 domain, RNase E(1–529), Alt, NudC, NudC*(V157A, E174A, E177A, E178A) and NudC(E178Q)

Isopropyl beta-d-thiogalactoside (IPTG)-induced *E.* *coli* One Shot BL21 (DE3) containing the respective plasmid (Supplementary Table 10) was cultured in LB medium at 37 °C. Protein expression was induced at an optical density at 600 nm (OD_600_) of 0.8, bacteria were collected after centrifugation for 3 h at 37 °C and lysed by sonication (30 s at 50% power, five times) in HisTrap buffer A (50 mM Tris-HCl, pH 7.8, 1 M NaCl, 1 M urea, 5 mM MgSO_4_, 5 mM β-mercaptoethanol, 5% glycerol, 5 mM imidazole, one tablet per 500 ml complete EDTA-free protease inhibitor cocktail (Roche)). The lysate was cleared by centrifugation (37,500*g* for 30 min at 4 °C) and the supernatant was applied to a 1 ml Ni-NTA HisTrap column (GE Healthcare). The protein was eluted with an imidazole gradient using an analogous gradient of HisTrap buffer B (HisTrap buffer A with 500 mM imidazole added) and analysed by SDS–PAGE.

Further protein purification was achieved by size-exclusion chromatography (SEC) through a Superdex 200 10/300 GL column (GE Healthcare) using SEC buffer containing 0.5 M NaCl and 25 mM Tris-HCl, pH 8. Fractions of interest were analysed by SDS–PAGE, pooled and concentrated in Amicon Ultra-4 centrifugal filters (molecular weight cut-off (MWCO) 10 kDa with centrifugation at 2,000 rpm and 4 °C). Protein concentration was measured with a NanoDrop ND-1000 spectrophotometer. Finally, proteins were stored in SEC buffer supplemented with 50% glycerol at −20 °C.

### Purification of ARH1 and ARH1(D55A, D56A)

*E.* *coli* BL21 DE3 pET28-ARH1 and BL21-pET28-ARH1 D55A, D56A (Supplementary Table 10) were grown to an OD_600_ = 0.6 at 37 °C and 175 rpm. Afterwards, bacteria were allowed to cool to room temperature for 30 min. Expression was induced with 1 mM IPTG, and bacteria were finally grown overnight at room temperature while shaking at 175 rpm. Bacteria were collected by centrifugation and proteins were purified in a similar way to rS1 variants.

### Purification of ModA

*E.* *coli* BL21 DE3 pET28-ModA (Supplementary Table 10) was grown to an OD_600_ = 1 at 37 °C with shaking at 175 rpm. Protein expression was induced with 0.5 mM IPTG and bacteria were collected by centrifugation after 3 h at 37 °C. Pelleted bacteria were resuspended in 50 mM NaH_2_PO_4_, pH 8, 300 mM NaCl, 1 mM DTT with one tablet per 500 ml complete EDTA-free protease inhibitor cocktail (Roche) and lysed by sonication (3× 1 min at 5% power). Lysates were centrifuged at 3,000*g* at 4 °C for 20 min. Sediments were washed by resuspension in 30 ml 50 mM Tris-HCl, pH 7.5, 2 mM EDTA, 100 mM NaCl, 1 M urea, 1 mM DTT and one tablet EDTA-free protease inhibitor (Roche), and centrifuged at 10,000*g* at 4 °C for 20 min. Pellets containing inclusion bodies were resuspended in 40 ml 100 mM Tris, pH 11.6, 8 M urea, transferred to 12–14 kDa MWCO dialysis bags (Roth) and dialysed overnight against 50 mM NaH_2_PO_4_, 300 mM NaCl. Protein solutions were centrifuged at 20,000*g* at 4 °C for 30 min. Supernatants were batch purified using disposable 10 ml columns (Thermo Fisher Scientific) packed with 2 ml Ni-NTA agarose (Jena Bioscience) and equilibrated with 10 column volumes of 50 mM NaH_2_PO_4_ (pH 8), 300 mM NaCl. Proteins were purified by washing the columns with 30 column volumes of 50 mM NaH_2_PO_4_, 300 mM NaCl, 15 mM imidazole, eluted with 5 ml 50 mM NaH_2_PO_4_, 300 mM NaCl, 300 mM imidazole and concentrated in Amicon (Merck Millipore) filters (MWCO 10 kDa with centrifugation at 2,000 rpm and 4 °C). Finally proteins were purified by SEC, as described for rS1.

### Purification of ModB and ModB(R73A, G74A)

*E.* *coli* BL21 DE3 pET28–ModB and *E.* *coli* BL21 DE3 pET28–ModB(R73A, G74A) (Supplementary Table 10) were grown to OD_600_ = 2.0 at 37 °C with shaking at 185 rpm and cooled to 4 °C while being shaken at 160 rpm for at least 30 min. Protein expression was induced by the addition of 1 mM IPTG. The cultures were then incubated for 120 min at 4 °C, with shaking at 160 rpm and bacteria were collected by centrifugation (4,000 rpm at 4 °C for 25 min). The ModB protein was purified from the supernatant as described for rS1 variants.

### Alphafold prediction of ModB structure

The Alphafold prediction of ModB structure was performed with AlphaFold2.ipynb (v.1.3.0, https://colab.research.google.com/github/sokrypton/ColabFold/blob/main/AlphaFold2.ipynb) with default parameters (use_templates = false, use_amber = false; msa_mode = MMseqs2 (UniRef+Environmental), model_type = “AlphaFold2-ptm”, max_msa = null, pair_mode = unpaired+paired, auto advanced settings). The ModB protein sequence was retrieved from Uniprot (primary accession: P39423). The ModB structure prediction model from rank_1 was further assessed using PyMol.

### In vitro ADP-ribosylation and RNAylation of rS1 and rL2 with ^32^P-labelled NAD, NAD–8-mer, NAD–Qβ RNA or NAD–10-mer–Cy5

rS1 (0.3 µM) was ADP-ribosylated in the presence of 0.25 μCi μl^−1^ ^32^P-NAD or RNAylated in the presence of one of 0.6 µM ^32^P-NAD–8-mer, 0.03 µM ^32^P-NAD–Qβ RNA or 0.8 µM NAD–10-mer–Cy5 (Supplementary Table 7) by 1.4 µM ModB and in 1× transferase buffer (10 mM Mg(OAc)_2_, 22 mM NH_4_Cl, 50 mM Tris-acetate pH 7.5, 1 mM EDTA, 10 mM β-mercaptoethanol and 1% glycerol) at 15 °C for at least 120 min. Samples (5 μl) were taken before the addition of ModB and after 1, 2, 5, 10, 30, 60 and 120 min, and mixed with 5 μl 2× Laemmli buffer to stop the reaction. Reactions were assessed by 12% SDS–PAGE and gels were stained in Instant Blue solution (Sigma-Aldrich) for 10 min. Radioactive signals were visualized using storage phosphor screens and a Typhoon 9400 imager. The intensity of the radioactive bands was quantified using ImageQuant 5.2 (GE Healthcare). The RNAylation with NAD-capped Cy5-labelled RNA was visualized with the ChemiDoc (Bio-Rad) Cy5 channel. Gels were then stained by Coomassie solution and imaged using the same system. In some cases, stain-free imaging of proteins in SDS gels was performed by 2,2,2-trichloroethanol (TCE) incorporated in the gel. TCE binds to tryptophan residues of the proteins, which enhances their fluorescence under ultraviolet light and thereby enables their detection^53^.

rL2 was ADP-ribosylated or RNAylated at the same settings using either 6.4 µM NAD or 6.4 µM NAD–8-mer as a substrate to modify 4.6 µM rL2 in the presence of 1.57 µM ModB for 4 h for LC–MS/MS measurements. For shift assays, 538 nM rL2 was RNAylated by 2.61 µM ModB in the presence of 6 µM NAD–8-mer. 12% SDS– PA gels were fixed with a solution of 40% ethanol and 10% acetic acid overnight and stained using Flamingo fluorescent protein dye (Bio-Rad) for up to 6 h and imaged with the ChemiDoc (Bio-Rad). Signal intensity was quantified in ImageLab (Bio-Rad). Where indicated, statistical tests were performed using two-sided *t*-tests in R (v.4.2.2) implemented in the ggpubr package (v.0.6.0) using a significance level of 0.05.

### In vitro RNAylation of *E.coli* RNA polymerase with NAD–10-mer–Cy5

We incubated 0.8 µM NAD–10-mer–Cy5 (Supplementary Table 7) with 0.5 µM of protein *E.* *coli* RNA polymerase (New England Biolabs) and 3 µM Alt or ModA in the presence of 1× transferase buffer at 15 °C for 60 min. Samples were taken before the addition of Alt or ModA and after 60 min incubation. The reactions were stopped by the addition of 1 volume of 2× Laemmli buffer. Reactions were analysed by 10% SDS–PAGE with rS1 RNAylated by ModB with NAD–10-mer–Cy5 as a reference protein. RNAylated proteins were visualized using the ChemiDoc (Bio-Rad) Cy5 channel. Afterwards, gels were stained in Coomassie solution and imaged using the same system.

### Analysis of protein rS1 self-RNAylation

In 20-μl reactions, 3.6 µM ^32^P-ADPr–8-mer (Supplementary Table 7) was incubated with either 2.6 µM rS1, 3.9 µM ModB or both 2.59 µM rS1 and 3.9 µM ModB in 1× transferase buffer. As a positive control, equal amounts of protein rS1 and ModB were incubated with 0.6 µM ^32^P-NAD–8-mer. All reactions were incubated at 15 °C for 60 min. Samples were taken before the addition of ModB or after 60 min, and reactions were stopped by adding one volume of 2× Laemmli buffer. Reactions were analysed by 12% SDS–PAGE and autoradiography imaging.

### RNAylation of protein rS1 with Qβ RNA (100-nucleotide–RNA) and specificity for the 5′-NAD cap

0.05 µM ^32^P-NAD–Qβ RNA, 0.15 µM 5′-^32^P-Qβ RNA or 0.15 µM 5′-^32^PPP-Qβ RNA (Supplementary Table 7) was incubated with 2.3 µM rS1 and 1.4 µM ModB in the presence of 1× transferase buffer at 15 °C for 60 min. Samples were taken before the addition of ModB and after 60 min, and reactions were stopped by adding 1 volume 2× Laemmli buffer. Reactions were analysed by 10% SDS–PAGE, applying rS1–^32^P-ADPr in 1× Laemmli buffer as a reference, and subsequent autoradiography imaging.

### Preparation of RNAylated and ADP-ribosylated rS1 for enzymatic treatments

ADP-ribosylation or RNAylation reactions were performed with radio-labelled substrates, washed and equilibrated in 1× transferase or 1× degradation buffer for further enzymatic treatments. The reactions were washed with four column volumes of the corresponding buffer by centrifugation at 10,000*g* at 4 °C in 10 kDa Amicon (Merck Millipore) filters. Proteins RNAylated with Cy5-labelled RNA were equilibrated in the same buffers using Zeba Spin desalting columns (7 kDa MWCO, 0.5 ml) (Thermo Fisher Scientific) according to the manufacturer’s instructions.

### Nuclease P1 digest of protein rS1 RNAylated with 100-nucleotide–RNA (rS1-100-nucleotide–RNA)

An rS1–100-nucleotide-RNA (^32^P) mixture (19 μl) was equilibrated in 1× transferase buffer and incubated with either 1 μl nuclease P1 or 1 μl Millipore water at 37 °C for 60 min. Samples were taken at the beginning and after 60 min, and reactions were stopped by adding one volume of 2× Laemmli buffer. Reactions were analysed by 10% SDS–PAGE, applying rS1–^32^P-ADPr in 1× Laemmli buffer as a reference, and subsequent autoradiography imaging.

### Tryptic digest of ^32^P-labelled rS1–8-mer and rS1–ADPr

Mixtures (19 μl) of both rS1 and rS1–8-mer (^32^P) and of rS1 and rS1–ADPr (^32^P) in 1× degradation buffer were incubated with either 0.2 µg Trypsin (Sigma, EMS0004, mass-spectrometry grade) or Millipore water as a negative control at 37 °C. Samples were taken before the addition of Trypsin/Millipore water and after 120 min. Reactions were stopped by adding one volume 2× Laemmli buffer to samples and were analysed by 12% SDS–PAGE and autoradiography imaging.

### Chemical removal of ADP-ribosylation and RNAylation in vitro

Aliquots from washed and equilibrated ADP-ribosylated (1 μl) and RNAylated (2 μl) (^32^P) rS1 were treated with either 10 mM HgCl_2_ or 500 mM NH_2_OH (refs. ^54,55^) at 37 °C for 1 h. Reactions were stopped by adding 2× Laemmli buffer and analysed by 12% SDS–PAGE.

### Enzymatic removal of ADP-ribosylation and RNAylation in vitro

Aliquots from washed and equilibrated (in 1× degradation buffer) ADP-ribosylated (1 μl) and RNAylated (2 μl) rS1 (^32^P) were treated with 0.5 U endonuclease P1 (Sigma-Aldrich)^56^ or 0.95 µM ARH1 or ARH3 (human recombinant, Enzo Life Science)^57^ in the presence of 10 mM Mg(OAc)_2_, 22 mM NH_4_Cl, 50 mM HEPES, 1 mM EDTA, 10 mM β-mercaptoethanol and 1% (v/v) glycerol in a total volume of 20 μl at 37 °C for 1 h. Enzymatic reactions were stopped by adding 2× Laemmli buffer and analysed by 12% SDS–PAGE.

### Inhibition of RNAylation and ADP-ribosylation with 3-methoxybenzamide

Reactions (20 μl) of 1.4 µM ModB and 2.3 µM protein rS1 with either 1 µM ^32^P-NAD–8-mer or 3 µM 5′-^32^P–8-mer (Supplementary Table 7) were incubated in the presence of 2 mM 3-MB (50 mM stock in DMSO) or the absence of the inhibitor (DMSO only) at 15 °C (ref. ^58^). Samples were taken before the addition of ModB and after 60 min. Reactions were stopped by the addition of 1 volume 2× Laemmli buffer and analysed by 12% SDS–PAGE.

### Effect of RNA secondary structure on RNAylation efficiency

We incubated 1.1 µM NAD–RNA–Cy5 (linear, 5′ overhang, 3′ overhang and blunt ends; Supplementary Table 7) with 0.9 µM rS1 and 0.4 µM ModB in 1× transferase buffer. Samples of 5 µl were taken before the addition of ModB protein and 2, 5, 10, 30, 60 and 120 min after the start of the reaction. The samples were directly mixed with one volume of 2× Laemmli buffer to stop the reaction. The conversion of the substrates was analysed by 12% SDS–PAGE, following visualization on ChemiDoc (Bio-Rad) in the Cy5 channel. The maximum observed signal intensity of RNAylated rS1 protein was used to determine the relative conversion for each of the analysed substrates at distinct time points.

### Culture of the *E.coli* B strain and infection with T4 phages

Precultures of *E.* *coli* B strain pTAC-rS1 (Supplementary Table 10) were incubated in LB medium with 100 µg ml^−1^ ampicillin at 37 °C and 185 rpm overnight. For the main cultures, 150 ml LB medium with 100 µg  ml^−1^ ampicillin were inoculated with preculture to an OD_600_ = 0.1. At OD_600_ = 0.4, protein expression was induced by the addition of 1 mM IPTG. At OD_600_ = 0.8, cultures were either infected with bacteriophage T4 at a multiplicity of infection (MOI) of 10 (20 ml phage solution) (DSM 4505, Leibniz Institute DSMZ) or not infected by adding 20 ml LB medium instead (negative control). Cultures were incubated for 20 min at 37 °C with shaking at 240 rpm. Bacteria were collected by centrifugation at 4,000*g* at room temperature for 15 min. Pellets were stored at −80 °C.

### Purification of His-tagged rS1 from infected *E.coli* strain B pTAC-rS1

Bacterial pellets were resuspended in 10 ml buffer A and lysed via sonication (1× 5 min, cycle 2 at 50% power). Lysates were centrifuged at 37,500*g* at 4 °C for 30 min. The supernatant was filtered through 0.45-μm filters (Sarstedt). rS1 from bacteriophage T4-infected or non-infected *E.* *coli* B strain was purified from the supernatant by gravity Ni-NTA affinity chromatography. Ni-NTA agarose slurry (1  ml, Thermo Fisher Scientific) was added to a 10 ml propylene column and equilibrated in buffer A. The supernatant was loaded onto the column twice. The column was washed with a mixture of 95% buffer A and 5% buffer B containing 29.75 mM imidazole. Protein was eluted from the column with 10 ml buffer B.

His-tagged-protein rS1 from T4-infected or uninfected *E.* *coli* B strain pTAC-rS1 was washed with two filter volumes of 1× degradation buffer (12.5 mM Tris-HCl, pH 7.5, 25 mM NaCl, 25 mM KCl, 5 mM MgCl_2_) by centrifugation in 10-kDa Amicon filters at 5,000*g* at 4 °C and concentrated to a final volume of 120 μl. The fractions were analysed by 12% SDS–PAGE analysis and the gel was stained in Instant Blue solution for 10 min and imaged immediately.

### Purification of His-tagged rS1 and rL2 for LC–MS/MS analysis

*E.* *coli* B strain with endogenously His-tagged rS1 and *E.* *coli* B strain expressing His-tagged rS1 WT, R139A or R139K were infected with T4 to an MOI of 5.0, as described above for 8 min. 100 ml culture was collected and the pellet resuspended in 1.5 ml Ni-NTA buffer A with 15 mM imidazole (50 mM Tris-HCl, pH 7.8, 1 M NaCl, 1 M urea, 5 mM MgSO_4_, 5 mM β-mercaptoethanol, 5% glycerol, 15 mM imidazole, one tablet per 500 ml complete EDTA-free protease inhibitor cocktail (Roche)). Cells were lysed by sonication (three times for 2 min at 80% power) and supernatant was cleared by centrifugation at 17,000*g* at 4 °C for 30 min. The supernatant was incubated with 75 µl Ni-NTA magnetic beads (Jena Bioscience) equilibrated in Ni-NTA buffer A with 15 mM imidazole for 1 h at 4 °C. Magnetic beads were washed seven times with 1 ml Ni-NTA buffer A with 15 mM imidazole and three times with Ni-NTA buffer without imidazole but with 4 M urea. Finally, protein was eluted by addition of Ni-NTA elution buffer (50 mM Tris-HCl, pH 7.8, 1 M NaCl, 1 M Urea, 5 mM MgSO_4_, 5 mM β-mercaptoethanol, 5% glycerol, 300 mM imidazole, one tablet per 500 ml complete EDTA-free protease inhibitor cocktail (Roche)). Protein was equilibrated in 1× transferase buffer with Zeba columns (7 kDa MWCO, 0.5 ml) according to the manufacturer’s instructions, and protein was digested with trypsin in a 1:20 ratio (w/w) at 37 °C for 3 h. Peptides were C18-purified using 50 mM triethylamine-acetate (pH 7.0) buffer in combination with 0–90% acetonitrile and Chromabond C18 WP spin columns (20 mg, Macherey Nagel). Purified peptides were dissolved in HPLC-grade H_2_O and subjected to LC–MS/MS analysis (see below).

In vitro RNAylated rS1 (D2) reactions in 1× transferase buffer were directly digested (without further purification) with 1 µg RNase A (Thermo Fisher Scientific) and 100 U RNase T1 (Thermo Fisher Scientific) at 37 °C for 1 h, following tryptic digest at 37 °C for 3 h in the same buffer with trypsin (Promega) in a 1:30 ratio (w/w) relative to the total protein content per sample. Peptides were purified with Chromabond C18 WP spin columns as described above and used for LC–MS/MS analysis (see below).

In vitro RNAylation reactions of rL2 with NAD–8-mer and ADP-ribosylation reactions were purified at similar settings to the proteins from T4 phage-infected *E.* *coli*. Here, reactions (200 µl) were incubated with 100 µl Ni-NTA beads equilibrated in 800 µl Ni-NTA buffer A with 10 mM imidazole and 40 U murine RNase inhibitor (New England Biolabs) at 4 °C for 1 h. Beads were washed eight times with 1 ml streptavidin wash buffer (50 mM Tris-HCl, pH 7.4, 8 M urea) at room temperature and protein was eluted with 130 µl Ni-NTA elution buffer. Purified proteins were rebuffered in 100 mM NH_4_OAc using Zeba spin desalting columns (7 kDa MWCO, 0.5 ml) according to the manufacturer’s instructions. rL2 samples were dissolved in 4 M urea in 50 mM Tris-HCl (pH 7.5) and incubated for 30 min at room temperature, followed by dilution to 1 M urea with 50 mM Tris-HCl (pH 7.5). 10 μg RNase A (Thermo Fisher Scientific) and 1 kU RNase T1 (Thermo Fisher Scientific) were added, following incubation for 4 h at 37 °C. For protein digestion, 0.5 µg trypsin (Promega) was added to each sample and digestion was performed overnight at 37 °C. Samples were adjusted to 1% acetonitrile (ACN) and to pH 3 using formic acid. Samples were cleaned up using C18 columns (Harvard Apparatus) according to the manufacturer’s instructions.

### LC–MS/MS analysis of His-tagged, in vitro RNAylated rS1 and rL2

Cleaned-up rS1 and rL2 peptide samples were dissolved in 2% ACN, 0.05% trifluoroacetic acid and subjected to LC–MS/MS analysis using an Orbitrap Exploris 480 mass spectrometer (Thermo Fisher Scientific) coupled to a Dionex Ultimate 3000 RSLCnano system. Peptides were loaded on a Pepmap 300 C18 trap column (Thermo Fisher Scientific) (flow rate, 10 µl min^−1^) in buffer A (0.1% (v/v) formic acid) and washed for 3 min with buffer A. Peptide separation was performed on an in-house-packed C18 column (30 cm; ReproSil-Pur 120 Å, 1.9 µm, C18 AQ; inner diameter, 75 µm; flow rate 300 nl min^−1^) by applying a linear gradient of buffer B (80% (v/v) ACN, 0.08% (v/v) formic acid). The main column was equilibrated with 5% buffer B for 18 s, the sample was applied and the column was washed for 3 min with 5% buffer B.

A linear gradient of 10–45% buffer B over 44 min was applied to elute peptides, followed by 4.8 min washing at 90% buffer B and 6 min at 5% buffer B. Eluting rS1 and rL2 peptides were analysed for 58 min in positive mode using a data-dependent top-20 acquisition method. The resolution for MS1 and MS2 were set to 120,000 and 30,000 full-width at half-maximum, respectively, and automatic gain control (AGC) targets were set to 10^6^ (MS1) and 10^5^ (MS2). The MS1 scan range was set to *m*/*z* = 350–1,600. Precursors were fragmented using 28% normalized, higher-energy collision-induced dissociation fragmentation. Other analysis parameters were set as follows: isolation width, 1.6 *m*/*z*; dynamic exclusion, 9 s; maximum injection times for MS1 and MS2, 60 ms and 120 ms, respectively.

For all measurements, the lock mass option (*m*/*z* 445.120025) was used for internal calibration.

### Analysis of in vitro RNAylated rS1 and rL2 MS data

MS data were analysed and validated manually using the OpenMS pipeline RNPxl and OpenMS TOPPASViewer^30^. Precursor mass tolerance was set to 6 ppm. MS/MS mass tolerance was set to 20 ppm. A neutral loss of 42.021798 Da (C_1_H_2_N_2_) at Arg residues was defined, as well as adducts of ribose minus H_2_O (78.010565 Da, C_5_H_2_O), ADP-ribose (541.06111 Da, C_15_H_2_1N_5_O_13_P_2_) and ADPr without adenine (485.97295 Da; C_10_H_17_O_16_P_3_)^31^. Results were filtered for a 1% false discovery rate on peptide spectrum match level. Ion chromatograms for rS1 peptides were extracted and visualized using Skyline (v.21.2.0.369)^59^.

### LC–MS/MS analysis of His-tagged rS1 isolated from T4-phage-infected *E.coli*

LC–MS/MS analysis of protein digests was performed on an Exploris 480 mass spectrometer connected to an electrospray ion source (Thermo Fisher Scientific). Peptide separation was done using the Ultimate 3000 nanoLC-system (Thermo Fisher Scientific), equipped with a packed-in-house C18 resin column (Magic C18 AQ 2.4 µm, Dr. Maisch). The peptides were eluted from a precolumn in backflush mode with a gradient from 98% solvent A (0.15% formic acid) and 2% solvent B (99.85% ACN, 0.15% formic acid) to 35% solvent B over 40 min and 90 min, respectively. The flow rate was set to 300 nl min^−1^. The data-dependent acquisition mode for label-free quantification was set to obtain one high-resolution MS scan at a resolution of 60,000 (*m*/*z* of 200) with scanning range from 350 to 1,650 *m*/*z*. MS/MS scans were acquired for the 20 most-intense ions (90 min gradient) and for the most-intense ions detected within 2 s (cycle 1 s, 40 min gradient). To increase the efficiency of MS/MS attempts, the charged-state screening mode was adjusted to exclude unassigned and singly charged ions. The ion accumulation time was set to 25 ms for MS and ‘auto’ for MS/MS scans. The AGC was set to 300% for MS survey scans and 200% for MS/MS scans.

Raw MS spectra were analysed using MaxQuant (v.1.6.17.0 and 2.0.3.0) using a fasta database of the targets proteins and a set of common contaminant proteins. The following search parameters were used: full tryptic specificity required (cleavage after Lys or Arg residues); three missed cleavages allowed; carbamidomethylation (C) set as a fixed modification; and oxidation (M; +16 Da), deamidation (N, Q; +1 Da) and ADP-ribosylation (K; +541 Da) set as variable modifications. MaxQuant was executed in the default setting. All MaxQuant parameters are listed in Supplementary Tables 1 and 2. The MS proteomics data have been deposited with the ProteomeXchange Consortium by the PRIDE partner repository under the dataset identifier PXD041714.

### Generation of *E.coli* B strain with endogenously His-tagged rS1

The *E.* *coli* B strain with endogenously His-tagged rS1 was created by homologous recombination of linear transforming DNA (tDNA) using the pRET/ET plasmid in the *E.* *coli* B strain. The linear tDNA was generated by fusion PCR aligning four fragments: 156 base pairs (bp) of the *rpsA* gene with an additional His-tag amplified from the pET28 rS1 vector (serving as the left homologous flank), a 70-bp fragment of the native rpsA terminator, the Flp-flanked kanamycin cassette from pKD4 and 140 bp of the 3′ flanking region of the *rpsA* gene (the right homologous flank). The primers used are indicated in Supplementary Table 9. The subsequent procedure for recombination is based on the protocol for the *E.* *coli* Gene Deletion Kit by RET/ET Recombination (Gene Bridges). In brief, *E.* *coli* B strain containing the pRED/ET plasmid was grown in LB medium supplemented with 100 µg ml^−1^ ampicillin at 30 °C. At OD_600_ = 0.35, L-arabinose was added to 0.33% (w/v) to induce expression of the RED/ET recombination system during growth at 37 °C for 1 h. Next, 1.4 ml culture was collected by centrifugation at 3,000*g* at 4 °C for 1 min, and cells were washed twice with 1 ml cold 10% glycerol and finally resuspended in 50 µl 10% glycerol. Cells were electroporated with 1 µg tDNA using a MicroPulser Electroporator (Bio-Rad) at standard settings (Ec1). Electroporated cells were immediately resuspended in 1 ml prewarmed LB medium and incubated at 37 °C with shaking at 600 rpm for 3 h. Finally, cells were plated on kanamycin (30 µg ml^−1^) LB–agar plates. Cells took 2 days to recover and grow. Successful recombination was evaluated by Sanger sequencing and correct protein expression was validated by pull-down and proteomics.

### RNAylomeSeq

Cultures (100 ml) of *E.* *coli* B strain with endogenously His-tagged rS1 (Supplementary Table 10) in LB medium supplied with 1 mM CaCl_2_, 1 mM MgCl_2_ and 30 µg ml^−1^ kanamycin were grown at 37 °C in 250 ml baffled Erlenmeyer flasks to an OD_60_ of around 0.8. T4 phage WT or T4 phage ModB(R73A, G74A) were added to an MOI of 5.0. For the uninfected negative control, the same volumes of LB medium were added to the cultures. Cultures were then incubated at 37 °C for 8 min and *E.* *coli* was collected by centrifugation at 3,000*g* for 13 min. Dried pellets were stored at −80 °C.

Pellets from the 100 ml culture infected with either WT T4 phage, T4 phage ModB(R73A, G74A) or the uninfected control (LB) were resuspended in 2 ml Ni-NTA wash buffer (10 mM imidazole, 50 mM Tris-HCl, pH 7.5, 1 M NaCl, 1 M urea, 5 mM MgSO_4_, 5 mM β-mercaptoethanol, 5% glycerol, pH 8.0, EDTA-free protease inhibitor (Roche, one tablet per 500 ml)) on ice and lysed by sonication (6 min, 50% power, 0.5 s pulse). The lysate was cleared from the cell debris by centrifugation at 21,000*g* at 4 °C for 30 min. Supernatant (1.9  ml), 50 µl Ni-NTA agarose beads (Jena Bioscience, equilibrated in Ni-NTA wash buffer), 80 U murine RNase inhibitor (New England Biolabs) and 4.72 µg rS1 D2 RNAylated with NAD-capped RNAI were combined and incubated at 4 °C in a rotary mixer for 30 min. Entire samples were transferred to Mobicol mini spin columns (MoBiTec). Beads were washed four times with 200 µl Ni-NTA wash buffer and subsequently eight times with 200 µl streptavidin wash buffer (50 mM Tris-HCl, pH 7.5, 8 M urea). Beads were equilibrated in standard ligation buffer (10 mM MgCl_2_, 50 mM Tris-HCl, pH 7.4) and blocked with bovine serum albumine (BSA) before 3′ RNA-adapter ligation at 4 °C overnight in the presence of standard ligation buffer, 50 mM β-mercaptoethanol, 0.05 µg µl^−1^ BSA, 15% (v/v) DMSO, 5 µM adenylated RNA-3′-adapter, 0.5 U µl^−1^ T4 RNL1 (New England Biolabs) and 10 U µl^−1^ T4 RNL2, tr. K227Q (New England Biolabs). Protein was rebound by the addition of NaCl to 1.5 M and incubation at 20 °C, with shaking at 400 rpm for 20 min. Beads were subsequently washed six times with streptavidin wash buffer and equilibrated in first strand buffer (50 mM Tris-HCl, pH 8.3, 3 mM MgCl_2_, 75 mM KCl) and blocked with BSA. Reverse transcription of protein-bound RNA was done in a 30-µl scale for 1 h at 40 °C using 10 U µl^−1^ Superscript IV Reverse Transcriptase (Invitrogen) in the presence of 5 µM RT primer, first strand buffer, 25 mM β-mercaptoethanol, 0.05 µg µl^−1^ BSA and 0.5 mM dNTPs. After incubation, NaCl was added to 1.5 M and the solution was incubated at 20 °C, with shaking at 400 rpm for 1 h to rebind RNA–cDNA hybrids. Beads were subsequently washed five times with 0.25× streptavidin wash buffer (2 M urea, 50 mM Tris-HCl, pH 7.5), equilibrated in ExoI buffer (10 mM Tris-HCl, pH 7.9, 5 mM β-mercaptoethanol, 10 mM MgCl_2_, 50 mM NaCl) and blocked with BSA. Residual RT primer was removed by ExoI digest with 1 U µl^−1^ *E.* *coli* ExoI (New England Biolabs) in ExoI buffer at 37 °C for at least 30 min. Finally, beads were washed with 200 µl 0.25× streptavidin wash buffer five times and subsequently with 200 µl immobilization buffer (10 mM Na-HEPES, pH 7.2, 1 M NaCl) three times. cDNA was eluted by incubation of beads in 100 µl 150 mM NaOH at 55 °C for 25 min and by washing with 100 µl MQ water. Eluate pH was neutralized by the addition of 0.05 volumes of 3 M NaOAc, pH 5.5. cDNA was removed from the residual protein by phenol–chloroform extraction and precipitated with 2.5 volumes of ethanol in the presence of 0.3 M NaOAc, pH 5.5 overnight. Precipitated cDNA was C-tailed using 1 U µl^−1^ TdT (Thermo Fisher) in the presence of 1.25 mM CTP and 1× TdT buffer at 37 °C for 30 min and subsequently inactivated at 70 °C for 10 min. 5 µM cDNA anchor (hybridization of forward and reverse anchor, Supplementary Table 9) was ligated to C-tailed cDNA in standard ligation buffer in the presence of 10 µM ATP and 1.5 U µl^−1^ T4 DNA Ligase (Thermo Fisher Scientific) at 4 °C overnight. Ligation reactions were inactivated at 70 °C for 10 min and cDNA was ethanol precipitated.

For the preparation of the Illumina RNAylomeSeq library, cDNA was amplified by PCR using 2 U Phusion Polymerase (Thermo Fisher Scientific) in the presence of 5% (v/v) DMSO, 200 µM dNTPs and 2,500 nM New England Biolabs Next Universal and Index Primer each (Primer Set 1, New England Biolabs). PCR products were purified by native PAGE and ethanol-precipitated. The double-stranded DNA (dsDNA) concentration was determined using a Quantus fluorometer (Promega) and library size was determined with the Bioanalyzer (Agilent). Equimolar amounts of each library were sequenced on a MiniSeq system (Illumina) using the MiniSeq High-Output Kit (150 cycles, Illumina) generating 20 million 151-bp single-end reads.

### Analysis of next-generation sequencing data

Next-generation sequencing (NGS) data were demultiplexed using bcl2fastq (v.2.20.0, Illumina). Fastq files were assessed using FastQC (v.0.11.9) and Illumina sequencing adapters were trimmed from reads using cutadapt (v.1.18). Reads were aligned to a reference genome composed of an *E.* *coli* K12 (U00096.3), bacteriophage T4 (NC_000866.4) and RNAI (our design) with hisat2 (v.2.2.1). Primary alignments were selected using samtools (v.1.7) and reads per genomic feature were counted with featureCounts (v.2.0.1 from Subread package). The resulting counts table was subjected to further analysis and data visualization in R (v.4.1.2). Read counts were normalized to the overall number of mapped reads per sample and to the respective read counts for the RNAI spike-in as follows:

$${{\rm{n}}{\rm{o}}{\rm{r}}{\rm{m}}{\rm{r}}{\rm{e}}{\rm{a}}{\rm{d}}{\rm{c}}{\rm{o}}{\rm{u}}{\rm{n}}{\rm{t}}}_{i,j}=\frac{{{\rm{r}}{\rm{e}}{\rm{a}}{\rm{d}}{\rm{c}}{\rm{o}}{\rm{u}}{\rm{n}}{\rm{t}}}_{i,j}\times {\rm{r}}{\rm{e}}{\rm{a}}{\rm{d}}{\rm{c}}{\rm{o}}{\rm{u}}{\rm{n}}{\rm{t}}({{\rm{R}}{\rm{N}}{\rm{A}}{\rm{I}}}_{j})}{\sum _{i}{{\rm{r}}{\rm{e}}{\rm{a}}{\rm{d}}{\rm{c}}{\rm{o}}{\rm{u}}{\rm{n}}{\rm{t}}}_{i,j}}$$

Data visualization was done with a custom R script^60^ and alignments were manually inspected in Integrative Genomics Viewer (IGV v.2.4.9). Hits were identified based on the following criteria: log_2_-transformed fold change (LFC) ≥ 1.5 comparing WT T4 and the T4 R73A, G74A mutant and log_2_-normalized mean expression among WT and R73A, G74A sample of one replicate ≥ −0.5.

### Quantitative PCR validation of NGS data

cDNAs from RNAylomeSeq were diluted 1:30 in Millipore water. Quantitative PCR was performed on 1 µl diluted cDNA in 10 µl scale in technical duplicates amplifying regions of 100–150 bp with the iTaq Universal SYBR Green Supermix (Bio-Rad), according to the manufacturer’s instructions, using the primers indicated in Supplementary Table 9. The log_2_-transformed difference in cycle-threshold values for WT T4 and T4 R73A, G74A infected samples from corresponding replicates was computed and an LFC ≥ 1 was set as a threshold for cDNA enrichment.

### Ribosome RNAylation and proteomic analysis of RNAylated proteins

70S ribosomes (4.3 µg µl^−1^) were RNAylated in transferase buffer in the presence of either 1 µM NAD–10-mer–Cy5 or 1 µM NAD–40-mer–Cy5 (Supplementary Table 7) by 0.05 µg µl^−1^ ModB at 15 °C for 90 min. RNAylated and non-RNAylated control samples were analysed using 12% SDS–PAGE. To identify RNAylated proteins, SDS–PAGE-separated protein bands were excised and proteins were digested in gel as described previously^61^. LC-MS was carried out on an Exploris 480 mass spectrometer connected to an Ultimate 3000 RSLCnano system with a Proflow upgrade and a nanospray flex ion source (all Thermo Scientific). Peptide mixtures were then analysed on the LC-MS system described above with a peptide-separating gradient of 30 min from 2% to 35% buffer B. Peptide separation was performed on a reverse-phase HPLC column (75 μm × 42 cm) packed in-house with C18 resin (2.4 μm, Dr. Maisch). Peptides were ionized at 2.3 kV spray voltage with a heated capillary temperature at 275 °C and funnel RF level at 40. MS survey scans were acquired with a resolution of 120.000 at *m*/*z* 200 and full MS AGC target of 300% with a maximal IT of 50 ms. The mass range was set to 350–1,650. Fragment spectra were acquired in data-dependent acquisition mode with a quadrupole isolation window of *m*/*z* = 1.5, an AGC target value of 200% and a resolution of 15.000, and fragmentation was induced with a normalized higher-energy collision-induced dissociation collision energy of 27%. MS raw data were searched with SEQUEST embedded in Proteome Discoverer 2.2 (Thermo Scientific) against a Uniprot *E.* *coli* protein database containing the bacteriophage T4 protein ModB. Spectral counts were exported from Scaffold Viewer and total spectral counts per sample were used to normalize spectral counts for all other proteins by division in Microsoft Excel 2016 followed by calculation of the ratio of normalized spectral counts from modified and unmodified bands.

### RNAylation of proteins in *E. coli* lysates

A fresh pellet from 40 ml *E.* *coli* B strain culture at an OD_600_ of around 0.8 was resuspended in 2 ml transferase buffer (10 mM Mg(OAc)_2_, 22 mM NH_4_Cl, 50 mM Tris-acetate, pH 7.5, 1 mM EDTA, 10 mM 2-mercaptoethanol, 1% glycerol). Cells were lysed by sonication (3× 2 min at 50% power, 0.5 s pulse) and the lysate was cleared from the cell debris by centrifugation at 27,670*g* at 4 °C for 30 min. The supernatant was used in RNAylation assays.

Lysate (100 µl) was incubated in the presence of 0.93 µM NAD–10-mer–Cy5 (0.47 µM with reference to the NAD-capped) or 0.93 µM P–10-mer–Cy5 (Supplementary Table 7), 0.37 U murine RNase inhibitor (New England Biolabs) and 0.69 µM ModB at 15 °C. Samples of 10 µl were taken before the addition of ModB and after 2, 5, 10, 20, 30 and 60 min, and were immediately resuspended in one volume of 2× Laemmli buffer. Samples were analysed by 12% SDS–PAGE applying the same reference (rS1 RNAylated with NAD–10-mer–Cy5) to each gel. The Cy5 signal was recorded using the Cy5 blot option of the ChemiDoc Imaging System at a manual exposure of 90 s. Gels were then stained in Coomassie solution and imaged with the same system.

*E.* *coli* lysates with various concentrations of ModB were processed and analysed by proteomics as described previously^38^.

### Determination of NAD concentrations in *E.coli* lysates

A dilution series of *E.* *coli* cell lysate was prepared in PBS. NAD was diluted in PBS starting from a 100 mM stock creating NAD solutions of 1,000 nM to 3.125 nM. The NAD solutions, the lysate dilution series and a PBS blank were assessed for their NAD concentrations using the NAD/NADH-Glo Assay (Promega), according to the manufacturer’s instructions in triplicates. Luminescence measurements were carried out on a Tecan plate reader (Spark) in a 384-well flat white plate. A linear fit (*R*^2^ = 0.9836) was performed for NAD concentrations between 400 nM and 4 nM with a linear correlation to intensity. The equation was used to calculate NAD concentrations for the *E.* *coli* lysate as the mean of the technical triplicates.

### Western blotting

Proteins were separated by 10% SDS–PAGE and gels were equilibrated in transfer buffer (25 mM Tris, pH 8.3, 192 mM glycine, 20 % (v/v) methanol). Polyvinylidene difluoride membranes with a pore size of 0.2 μm (GE Healthcare) were activated in methanol for 1 min and equilibrated in transfer buffer. Proteins were transferred from gels to the membranes in a semi-dry manner at 300 mA for 1.5 h, unless indicated differently. After the transfer, membranes were dehydrated by soaking in methanol and washed twice with TBS-Tween (TBS-T; 10 mM Tris-HCl, pH 7.5, 150 mM NaCl, 0.05% (v/v) Tween 20). Afterwards, 10 ml blocking buffer (5% (w/v) milk powder in TBS-T) were added to the membranes and incubated at room temperature for 1 h. To detect ADP-ribosylated proteins, membranes were incubated with a 1:10,000 dilution of anti-pan-ADPr binding reagent MABE1016 (Merck) in 10 ml washing buffer (1% (w/v) milk powder in TBS-T) at 4 °C overnight^62^. Membranes were washed and incubated with 10 ml of a 1:10,000 dilution of the horseradish peroxidase–goat-anti-rabbit IgG secondary antibody (Advansta) in washing buffer at room temperature for 1 h. Afterwards, membranes were washed with PBS. ADP-ribosylated proteins were visualized by chemiluminescence using the SignalFire ECL Reagent or the SignalFire Elite ECL Reagent (Cell Signaling Technology), according to the manufacturer’s instructions.

If proteins in SDS–PAGE gels needed to be visualized before blotting, a TCE staining method^53^ was used. Resolving gels were supplemented with 0.5% (v/v) TCE. For visualization, gels were activated by ultraviolet transillumination (with a wavelength of 300 nm) for 60 s. Proteins then showed fluorescence in the visible spectrum.

### Quantification of RNAylation

rS1 proteins were isolated from *E.* *coli* strain B pTAC rS1 bacteria (Supplementary Table 10) that were either uninfected or infected with bacteriophage T4. rS1 (1.5 µM) was treated with 1 µM ARH1 in the presence of 12.5 mM Tris-HCl, pH 7.5, 25 mM NaCl, 25 mM KCl and 5 mM MgCl_2_. Alternatively, rS1 (1.5 µM) was treated with 0.5 U endonuclease P1 in 100 mM Mg(OAc)_2_, 220 mM NH_4_Cl, 500 mM HEPES, pH 7.5, 10 mM EDTA, 100 mM β-mercaptoethanol and 10% glycerol. Digests were incubated at 37 °C for 2 h. Afterwards, digests were precipitated by the addition of nine volumes of ethanol and precipitated by centrifugation (14,000 pm) at 4 °C for 1 h. Protein pellets were resuspended in 10 μl 1× Laemmli buffer and analysed by Western blotting. ADPr modifications were detected by the primary antibody MABE1016 (Merck) as described above. The pan-ADPr signals for ADP-ribosylated rS1 were normalized to the corresponding band intensities in the TCE stain. Normalized intensities for untreated rS1 were then divided by the intensity for P1-treated rS1 to yield the fractions of ADP-ribosylated and RNAylated rS1 for the two modifications.

### Phage mutagenesis

The CRISPR–Cas9 spacer plasmids were generated by introducing the *modB* spacer sequence into the DS-SPCas plasmid (Addgene, 48645) (Supplementary Table 10). The *modB-*carrying vector pET28_ModB was used as a donor DNA for homologous recombination in CRISPR–Cas9-mediated mutagenesis. The pET28_ModB plasmid was modified by site-directed mutagenesis, during which point mutations R73A and G74A were exposed to *modB*. The R73A mutation led to the inactivation of ADP-ribosyltransferase activity. The G74A mutation was located in the protospacer adjacent motif and was required to prevent the cleavage of donor DNA by Cas9 nuclease. The applied primers are listed in Supplementary Table 9. The resulting plasmids were sequenced and transformed into *E*. *coli* BL21 (DE3).

The CRISPR–Cas9-mediated mutagenesis was based on previous work^33^. The DS_SPCas_ModB plasmid with the target spacer sequence and the donor plasmid pET28a_ModB_R73A/G74A were co-transformed into *E.* *coli* DH5α. The cells were further infected by bacteriophage T4 (1:10,000 phages:cells), and the plaque assay was done. The plates were incubated overnight at 37 °C and the resulting plaques were screened for mutants. Single plaques were picked by sterile pipet tips and transferred into 200 µl Pi–Mg buffer (26 mM Na_2_HPO_4_, 68 mM NaCl, 22 mM KH_2_PO_4_, 1 mM MgSO_4_, pH 7.5) supplied with 2 µl CCl_3_H. The samples were incubated at room temperature for 1 h. Next, 2 µl of the sample was transferred to a new PCR tube and heated to 95 °C for 10 min. The sample was further used for DNA amplification using PCR (primers used are listed in Supplementary Table 9). The amplified DNA was purified by agarose gel electrophoresis and submitted for Sanger sequencing.

### Plaque assay

The *E.* *coli* culture of interest was grown to an OD_600_ of around 0.8–1.0. Next, 300 µl of the culture was infected with 100 µl of WT T4 phage or T4 ModB(R73A, G74A) (Supplementary Table 10) mutant, with either defined or unknown MOI. The bacteria–phage suspension was incubated at 37 °C for 7 min and subsequently transferred to 4 ml LB soft agar (0.75%), mixed and poured onto an LB-agar plate (1.5% LB agar). The plates were incubated at 37 °C overnight and validated the following day.

### Time course of T4-mediated lysis of *E.coli*

LB medium (100 ml in 500-ml baffled flasks) was inoculated with *E.* *coli* B culture overnight to OD_600_ = 0.1 and was then incubated at 37 °C with shaking at 180 rpm until OD_600_ = 0.8 was reached. The culture was cooled to room temperature and infected by either WT T4 phages or T4 ModB(R73A, G74A) mutants (Supplementary Table 10) to an MOI of 5. The culture was further incubated at room temperature with shaking at 150 rpm. Cell lysis was tracked by measuring the OD_600_ at different times of infection (0–200 min after infection). The experiment was run in biological triplicates.

### Burst-size determination

LB medium (100  ml in 500-ml baffled flasks) was inoculated with *E.* *coli* B culture overnight to OD_600_ = 0.1 and was then incubated at 37 °C with shaking at 180 rpm until OD_600_ = 0.8 was reached, as above. The culture was infected either by WT T4 phages or T4 ModB(R73A, G74A) mutant (Supplementary Table 10) to an MOI of 0.01 and further incubated at 37 °C without shaking.

To determine the total number of infective centres, *T*_0_ (comprising unadsorbed and already adsorbed phages), at 5 min after infection, 100 µl of infected culture was used to reinfect 300 µl *E.* *coli* B cells (OD_600_ = 1.0) with a subsequent plaque assay. The number of unadsorbed phages (*U*) was determined by transferring 1 ml infected culture to 50 µl CCl_3_H. In this way, *E.* *coli* cells were disrupted, after which the unadsorbed phages remained intact and were used for plaque assay. *T*_0_−*U*, represents the number of initially infected centres. The number of unadsorbed phages (*U*_*x*min_) was continuously traced during infection and used to calculate the number of T4-phage progeny (T4-phage progeny = *U*_*x*min_/(*T*_0_−*U*_5min_). The time point at which the first increase in phage number was observed was treated as the first burst time point and was used to calculate the phage burst size (burst size  =  *U*_burst1_/(*T*_0_−*U*_5min_)).

Data were plotted using OriginPro 2020b software. Error bars represent s.d. of the means for three biological replicates. For selected time points, statistical tests were done as two-sided *t*-tests in R (v.4.2.2) implemented in the ggpubr package (v.0.6.0) using a significance level of 0.05.

### Phage adsorption kinetics

LB medium (100 ml in 500-ml baffled flasks) was inoculated with *E.* *coli* B culture overnight to an OD_600_ = 0.1 and incubated at 37 °C with shaking at 180 rpm until OD_600_ = 0.8 was reached, as above. The culture was cooled to room temperature and infected by either WT T4 phages or T4 ModB(R73A, G74A) mutants (Supplementary Table 10) to an MOI of 0.1. Immediately after infection, 100 µl of the culture was used to determine the number of total infective centres, *T*_0_, by plaque assay. Then 100 µl of the culture was taken at different time points of infection (0–25 min after infection) and 5 µl CCl_3_H was added to disrupt *E.* *coli* cells. This suspension was subsequently used to determine the number of unadsorbed phages (*U*_*x*min_) by plaque assay. The calculation of the adsorption rate was performed as follows: adsorption rate (%) = 100% − (*U*_xmin_/*T*× 100%).

Data were plotted using OriginPro 2020b software. Error bars represent s.d. of the means for three biological replicates. For selected time points, statistical tests were done as two-sided *t*-tests in R (v.4.2.2) implemented in the ggpubr package (v.0.6.0) using a significance level of 0.05.

### Reporting summary

Further information on research design is available in the Nature Portfolio Reporting Summary linked to this article.

## Online content

Any methods, additional references, Nature Portfolio reporting summaries, source data, extended data, supplementary information, acknowledgements, peer review information; details of author contributions and competing interests; and statements of data and code availability are available at 10.1038/s41586-023-06429-2.

### Supplementary information

Supplementary Information This file contains Supplementary Figs. 1–4, full legends for Supplementary Tables 1–6 (files supplied separately) and Supplementary Tables 7–10.

Reporting Summary

Supplementary Table 1 MaxQuant output for LC–MS/MS analysis of endogenously His-tagged rS1 from T4 phage-infected *E.* *coli* B strain.

Supplementary Table 2 MaxQuant output for LC–MS/MS analysis of His-tagged rS1 WT, rS1(R139A) and rS1(R139K) mutants from T4 phage-infected *E.* *coli*.

Supplementary Table 3 In vitro ADP-ribosylation and RNAylation sites in rS1 protein as identified by LC–MS/MS analysis.

Supplementary Table 4 Genes identified to contribute to the RNAylome by RNAylomeSeq.

Supplementary Table 5 MaxQuant output for LC–MS/MS analysis of *E.* *coli* cell lysate with addition of ModB to various concentrations.

Supplementary Table 6 In vitro ADP-ribosylation and RNAylation sites in rL2 protein as identified by LC––MS/MS analysis.

### Source data

Source Data Fig. 2

Source Data Fig. 4

Source Data Fig. 5

Source Data Extended Data Fig. 8

Source Data Extended Data Fig. 10

## Figures and Tables

**Extended Data Table 1:**
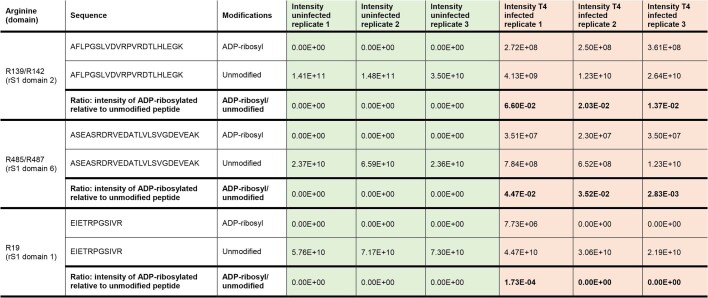
ADP-ribosylation of endogenously His-tagged rS1 during T4 phage infection

MaxQuant intensities are presented for T4 phage-infected and uninfected samples in biologically independent triplicates (n = 3). R139/R142 located in rS1 domain 2 and R485/R487 in rS1 domain 6 appear as ADP-ribosylation sites on rS1 *in vivo* in all three replicates. The ratio comparing intensity of ADP-ribosylated and unmodified species of the same peptide is computed for each sample and peptide.

**Extended Data Table 2:**
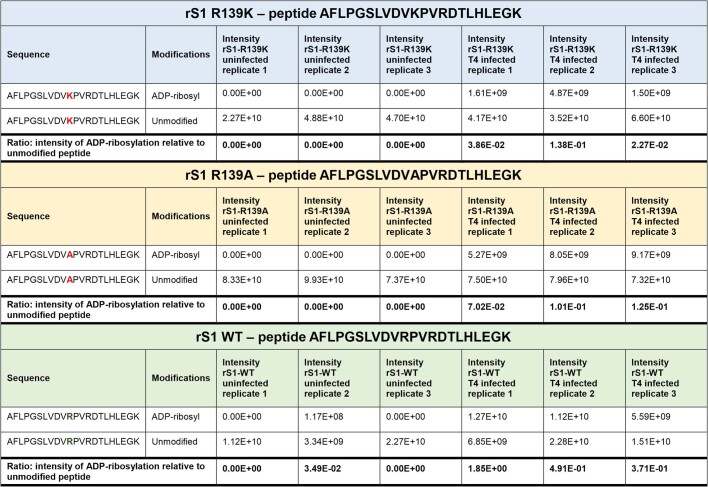
ADP-ribosylation of rS1-WT, -R139K and -R139A during T4 phage infection

MaxQuant intensities are presented for T4 phage-infected and uninfected samples in biologically independent triplicates (n = 3) only for the respective peptide of the R139 mutation site which is expected for the respective rS1 version. ADP-ribosylation of the peptide in rS1 is observed *in vivo* in all three replicates. However, ADP-ribosylation at position 139 is abolished by R139A or R139K mutations (mutation indicated in red). The intensity of ADP-ribosylated peptide relative to the intensity of the corresponding unmodified peptide species is at least 3-fold reduced upon R139 mutation. One may speculate that R142 is nevertheless ADP-ribosylated in the mutated rS1 proteins but overall ADP-ribosylation yield at the peptide may be reduced as the potentially predominant ADP-ribosylation site (R139) is not available for modification.

**Extended Data Table 3:**
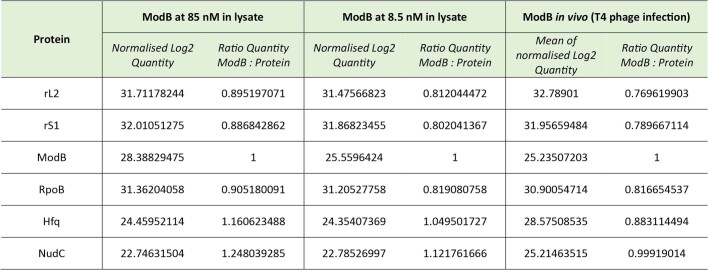
Comparison of ModB protein intensities in lysate assay and *in vivo* via proteomics

Normalised Log2 intensities for selected *E. coli* proteins and ModB found in proteomic analysis of *E. coli* cell lysates for *in vitro* RNAylation (n = 1) and in a previously published data set of the *E. coli* and T4 phage proteome 5 min post-infection^38^ (n = 3). Intensity of ModB is divided by the intensity for various *E. coli* proteins. At 8.5 nM ModB, the ratios approximate conditions found *in vivo*. Raw data is presented in Supplementary Table 5.

## Data Availability

The datasets generated and/or analysed during the current study are available from the corresponding author on reasonable request. NGS data are accessible via GEO record GSE214431. LC–MS/MS raw data for the measurements of rS1 ADP-ribosylation in vivo, in-gel digest and estimation of ModB abundance have been deposited in PRIDE with the accession code PXD041714. LC–MS/MS raw data for measurements of in vitro ADP-ribosylated and RNAylated rS1 and rL2 have been deposited in PRIDE with the accession code PXD038910. Reference genomes for *E.* *coli* (U00096.3) and T4 phage (NC_000866.4) were retrieved from NCBI. Protein structures (2MFI, 2MFL, 2KHI, 5XQ5, 2KHJ, 7K00 and 6H4N) were downloaded from PDB using the indicated accession code (https://www.rcsb.org/). *E.* *coli* K12 pan proteome (UP000000625) and selected protein sequences were retrieved from Uniprot (https://www.uniprot.org/). Supplementary information is available, including raw gel and blot images. Source data are provided with this paper.

## Code availability

The custom R code used to analyse the RNAylomeSeq data is publicly available on Zenodo^60^.
