# Supplementary material for: A viral ADP-ribosyltransferase attaches RNA chains to host proteins
Source: Nature. 2023 Aug 16;620(7976):1054–62. doi: 10.1038/s41586-023-06429-2 (PMC10468400; doi:10.1038/s41586-023-06429-2)
Supplement: Supplementary file 1 — This file contains Supplementary Figs. 1–4, full legends for Supplementary Tables 1–6 (files supplied separately) and Supplementary Tables 7–10. [file 41586_2023_6429_MOESM1_ESM.pdf]

nature portfolio

https://doi.org/10.1038/s41586-023-06429-2

Supplementary information

A viral ADP-ribosyltransferase attaches RNA chains to host proteins

In the format provided by the authors and unedited

nature portfolio

https://doi.org/10.1038/s41586-023-06429-2

Supplementary information

A viral ADP-ribosyltransferase attaches RNA chains to host proteins

In the format provided by the authors and unedited

SUPPLEMENTARY INFORMATION

A viral ADP-ribosyltransferase attaches RNA chains to host proteins

Maik Wolfram-Schauerte[1,2 +], Nadiia Pozhydaieva[1], Julia Grawenhoff[2], Luisa M. Welp[3,4], Ivan Silbern[3,4], Alexander Wulf[3], Franziska A. Billau[2], Timo Glatter[1], Henning Urlaub[3,4], Andres Jäschke[2*], Katharina Höfer[1,2,5* +]

1 Max Planck Institute for Terrestrial Microbiology, 35043 Marburg, Germany.
2 Institute of Pharmacy and Molecular Biotechnology (IPMB), Heidelberg University, 69120 Heidelberg, Germany
3 Max Planck Institute for Multidisciplinary Sciences, 37077 Göttingen, Germany.
4 Institute of Clinical Chemistry, University Medical Center, 37075, Göttingen, Germany.
5 SYNMIKRO, Center for Synthetic Microbiology, 35043 Marburg, Germany
+ These authors contributed equally to this work.
* Corresponding authors: Andres Jäschke, PhD, jaeschke@uni-hd.de; Katharina Höfer, PhD, Katharina.Hoefer@synmikro.mpi-marburg.mpg.de

SUPPLEMENTARY INFORMATION – TABLE OF CONTENTS

**Supplementary Figure 1**: Raw gel and blot images used for figures in this study.	3
**Supplementary Figure 2**: Statistical data on the Alphafold prediction model of ModB structure shown in Extended Data Fig. 1f.	39
**Supplementary Figure 3**: IGV coverage plots for RNAylomeSeq data.	40
**Supplementary Figure 4**: Statistical tests for phenotype of T4 phage ModB R73A, G74A mutant shown in Fig. 5 c-e.	40
**Legend for Supplementary Table 1**: MaxQuant Output for LC-MS/MS analysis of endogenously His-tagged rS1 from T4 phage-infected *E. coli* B strain.	41
**Legend for Supplementary Table 2**: MaxQuant Output for LC-MS/MS analysis of His-tagged rS1-WT, -R139A and -R139K mutants from T4 phage-infected *E. coli*.	41
**Legend for Supplementary Table 3**: *In vitro* ADP-ribosylation and RNAylation sites in rS1 protein as identified by LC-MS/MS analysis.	41
**Legend for Supplementary Table 4**: Genes identified to contribute to the RNAylome by RNAylomeSeq.	41
**Legend for Supplementary Table 5**: MaxQuant Output for LC-MS/MS analysis of *E. coli* cell lysate with addition of ModB to various concentrations.	41
**Legend for Supplementary Table 6**: *In vitro* ADP-ribosylation and RNAylation sites in rL2 protein as identified by LC-MS/MS analysis.	42
**Supplementary Table 7**: RNAs used in this study.	43
**Supplementary Table 8**: Genomic DNA sequence of ARTs, rS1 variants and ADP-ribose hydrolases.	44
**Supplementary Table 9**: Primers used in this study.	51
**Supplementary Table 10**: Strains and plasmids used in this study.	56
**References**	57

Supplementary Figure 1: Raw gel and blot images used for figures in this study.

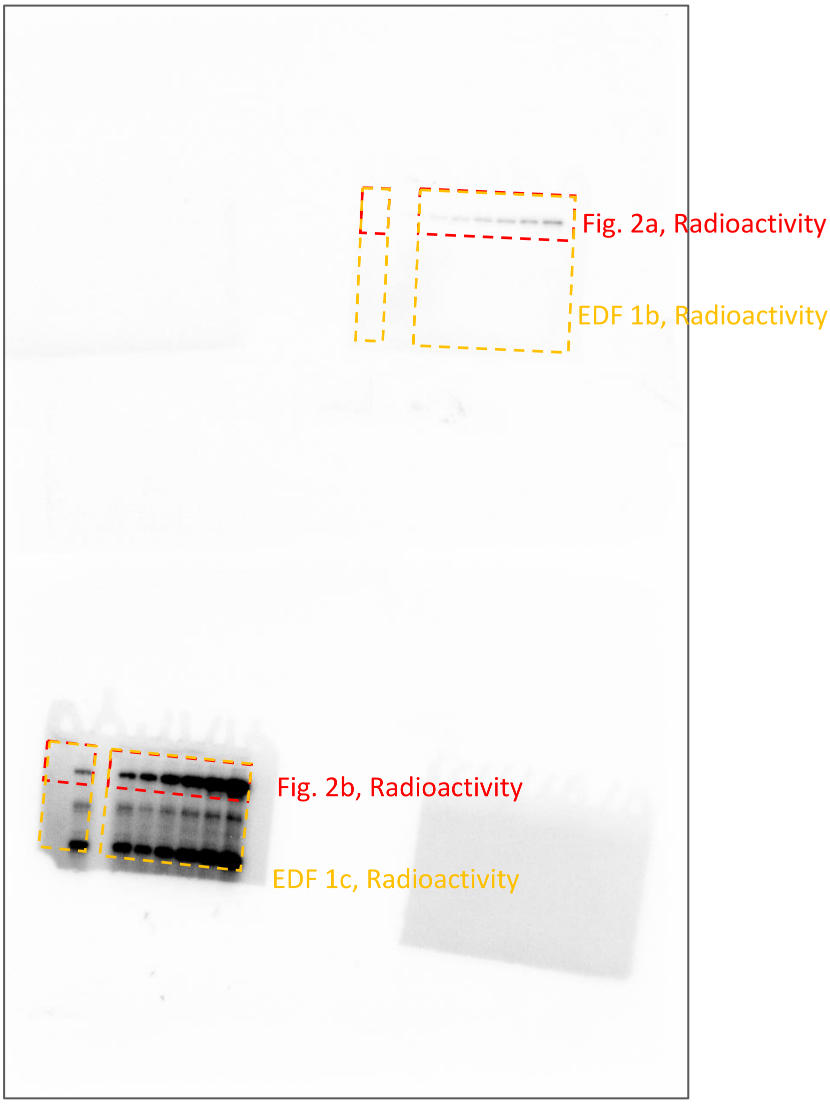

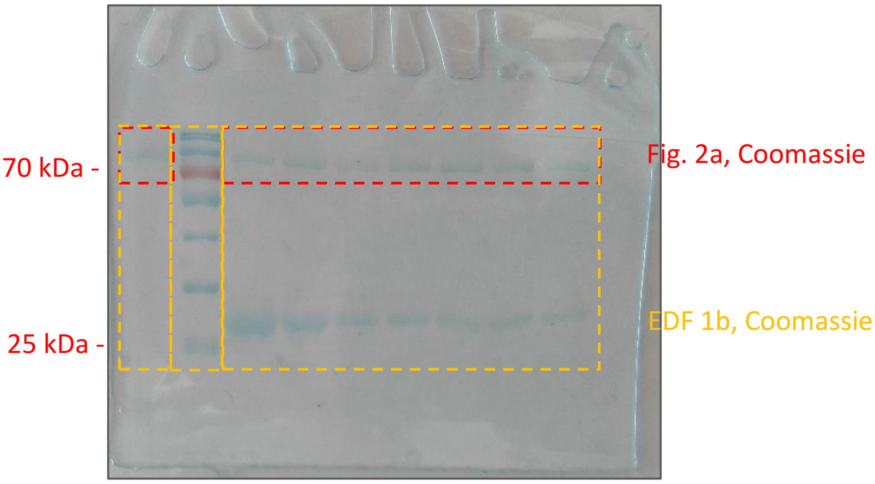

Used in Fig. 2a,b (red) and Extended Data Fig. 1b,c (yellow)

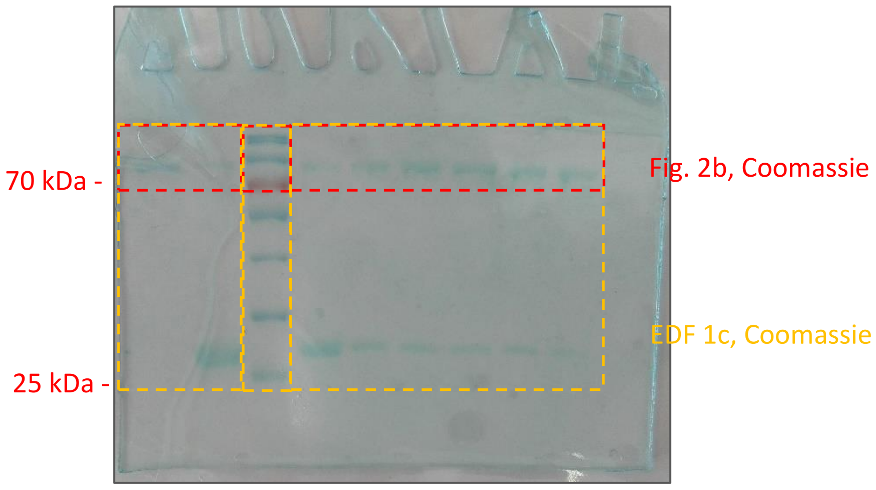

Used in Fig. 2a,b (red) and Extended Data Fig. 1b,c (yellow)

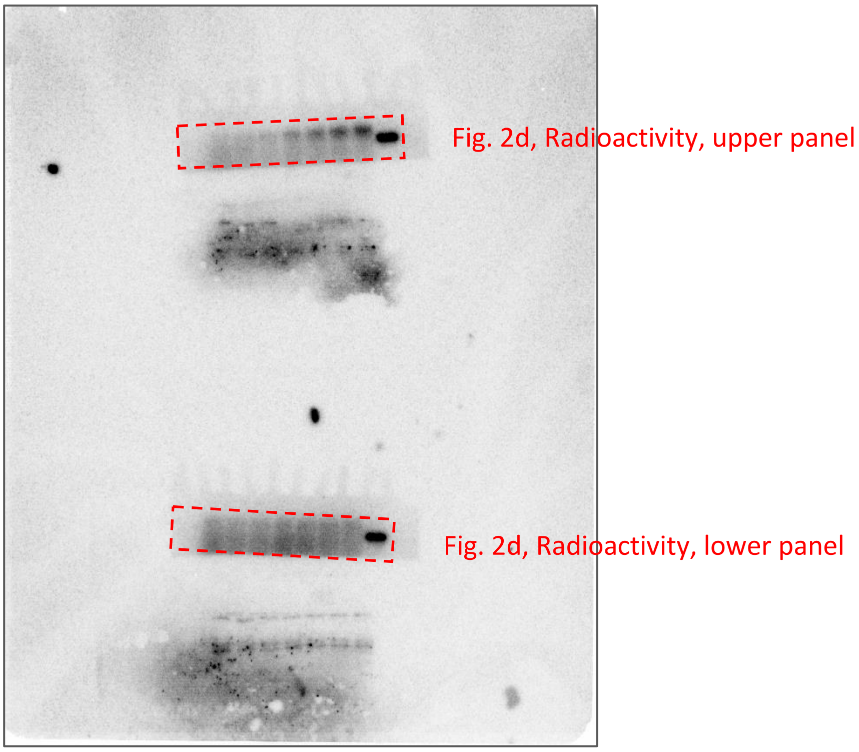

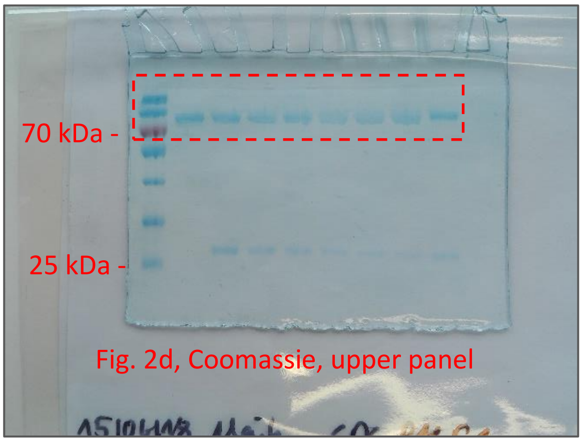

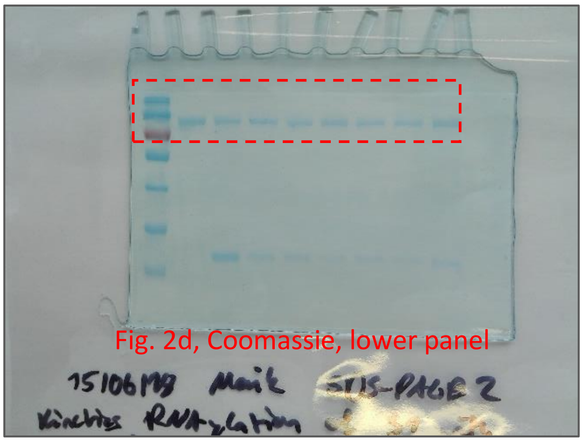

Used in Fig. 2d

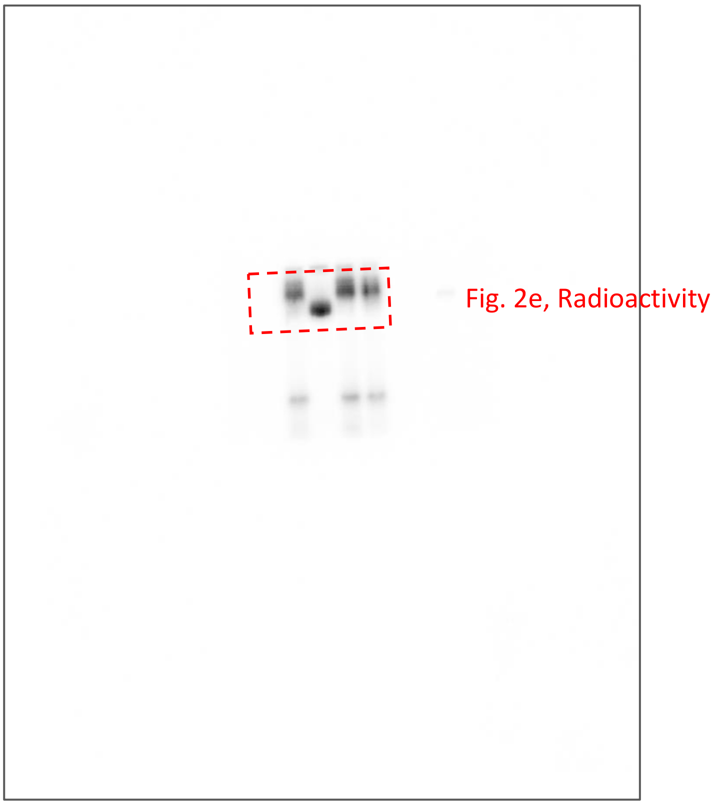

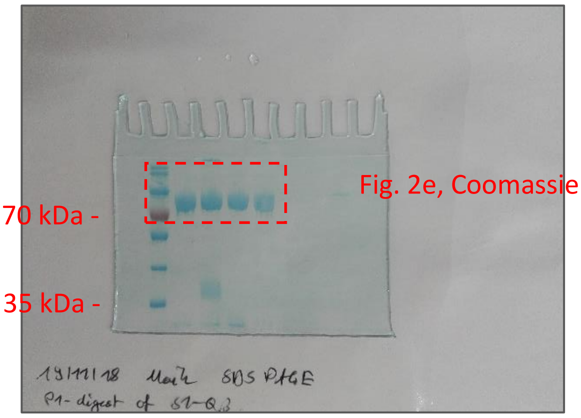

Used in Fig. 2e, right panel

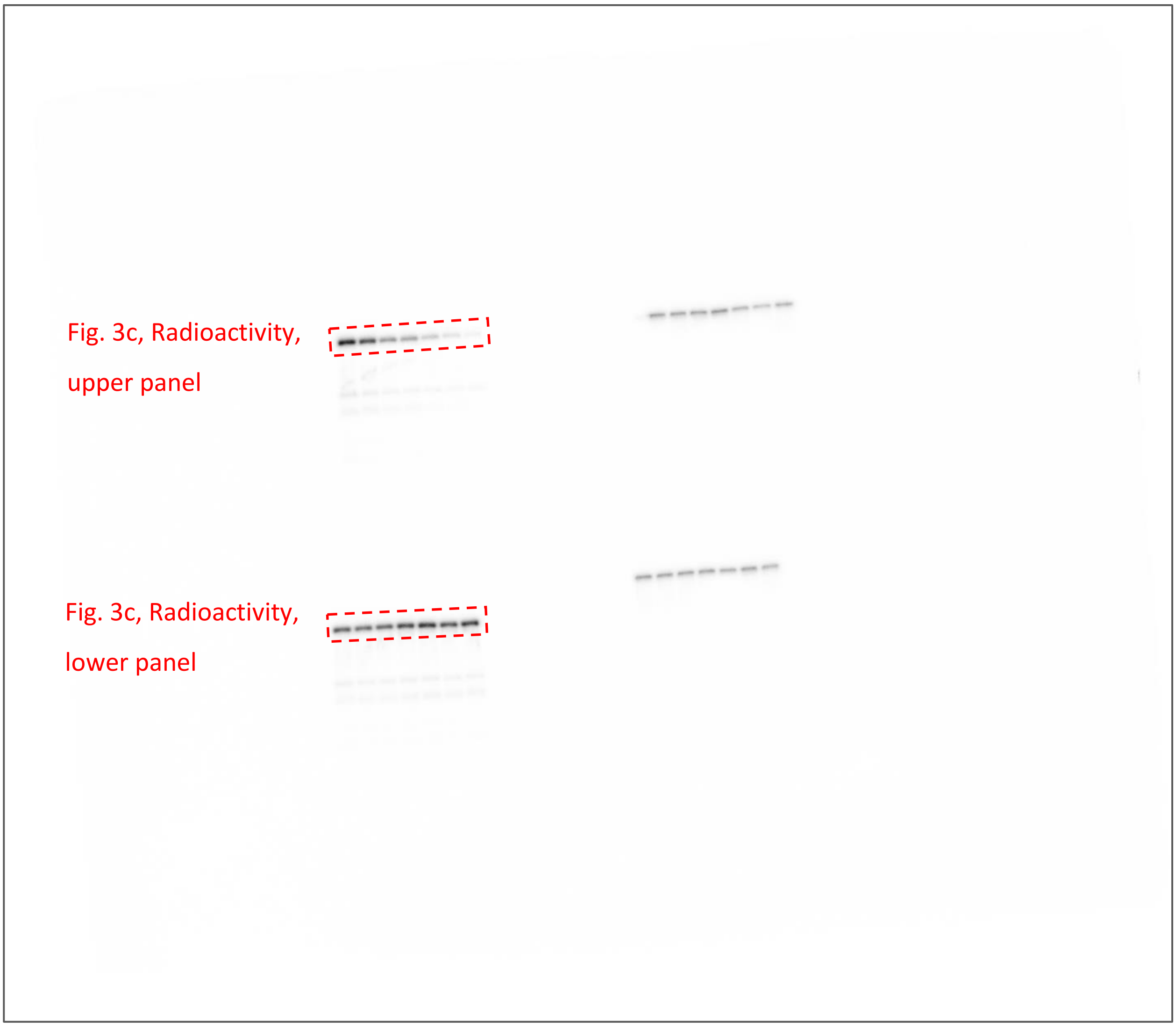

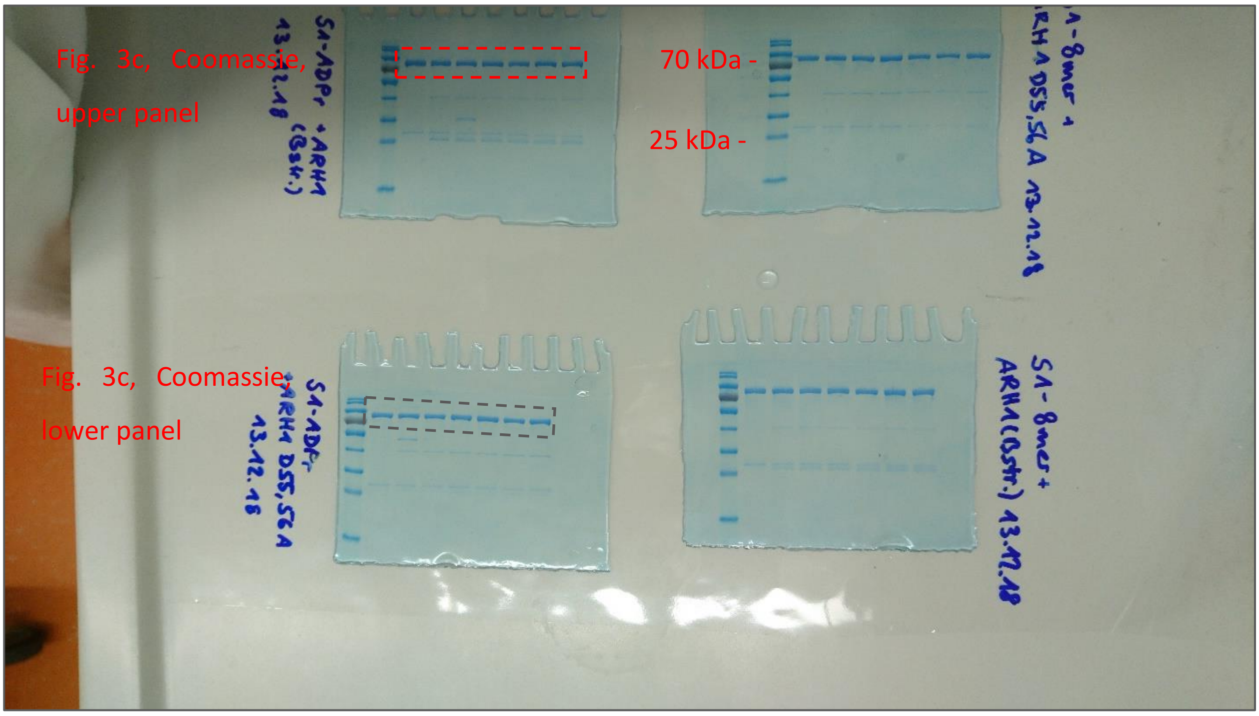

Used in Fig. 3c

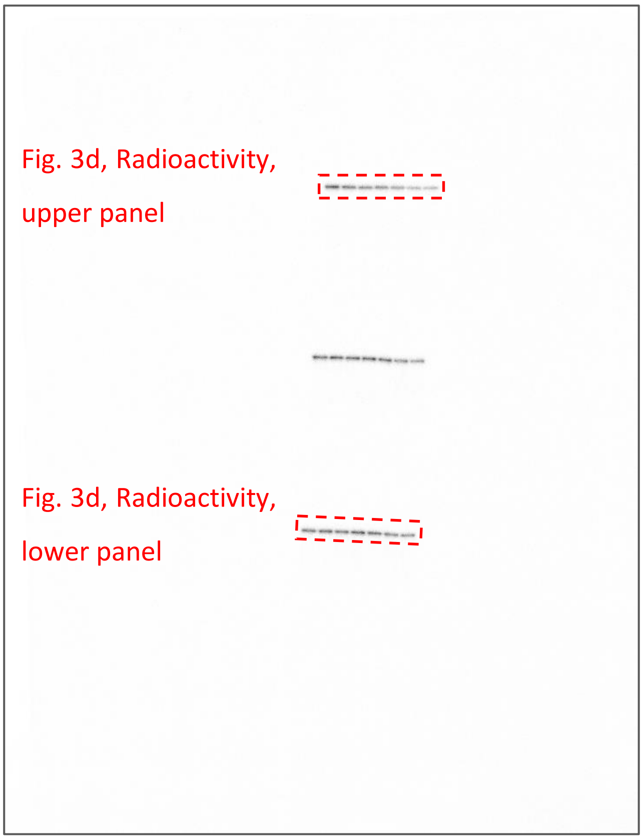

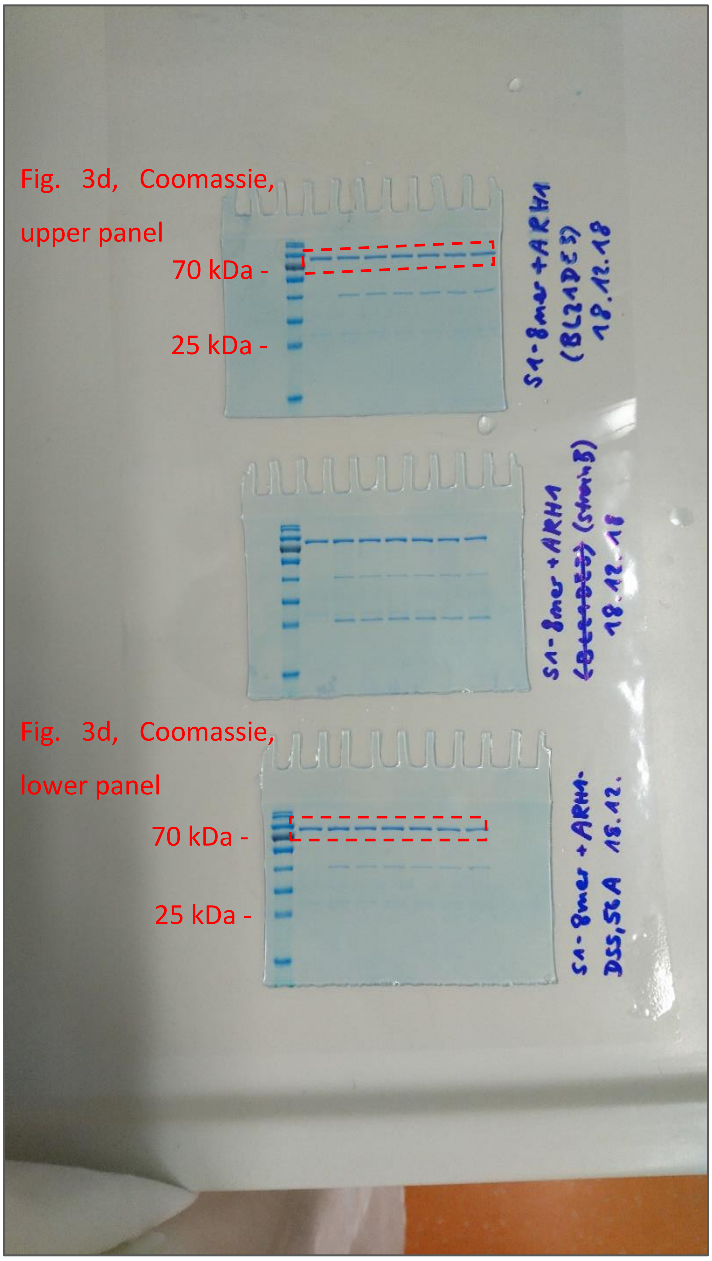

Used in Fig. 3d

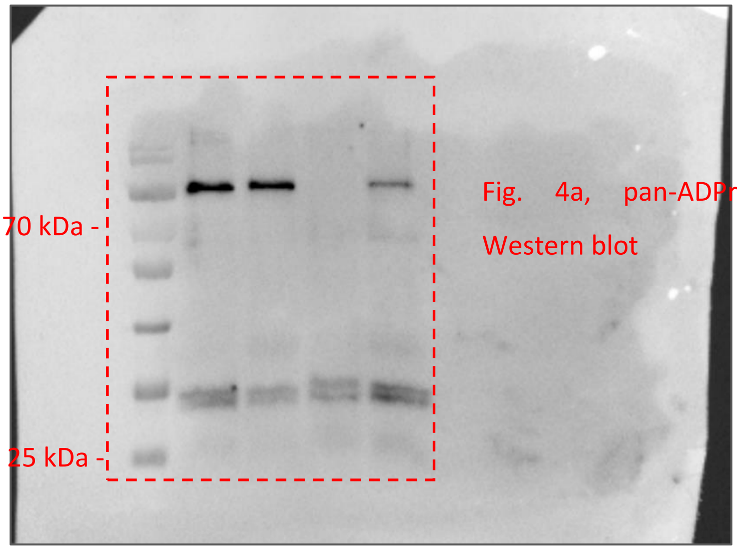

Used in Fig. 4a

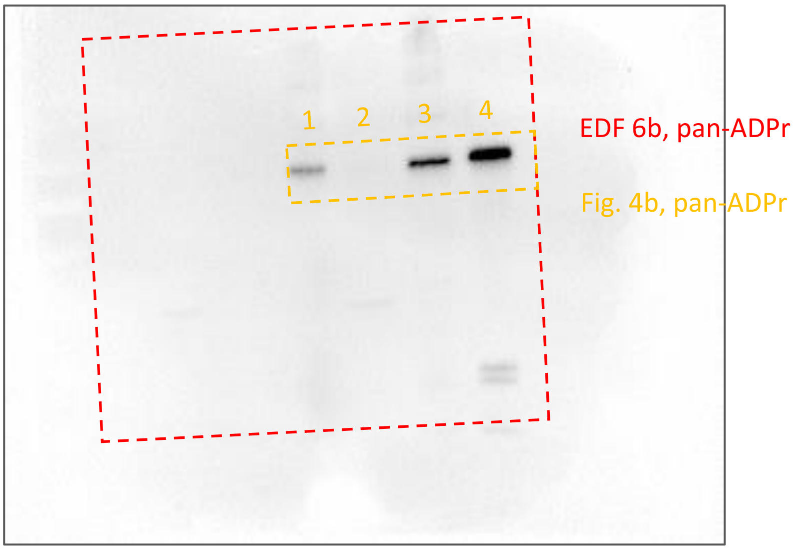

Used in Fig. 4b (yellow) and EDF 6b (red)

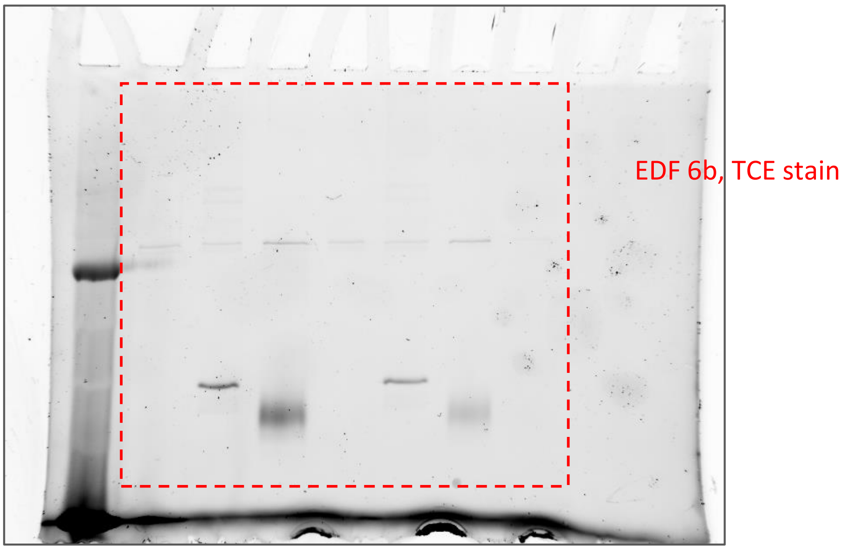

Used in EDF 6b

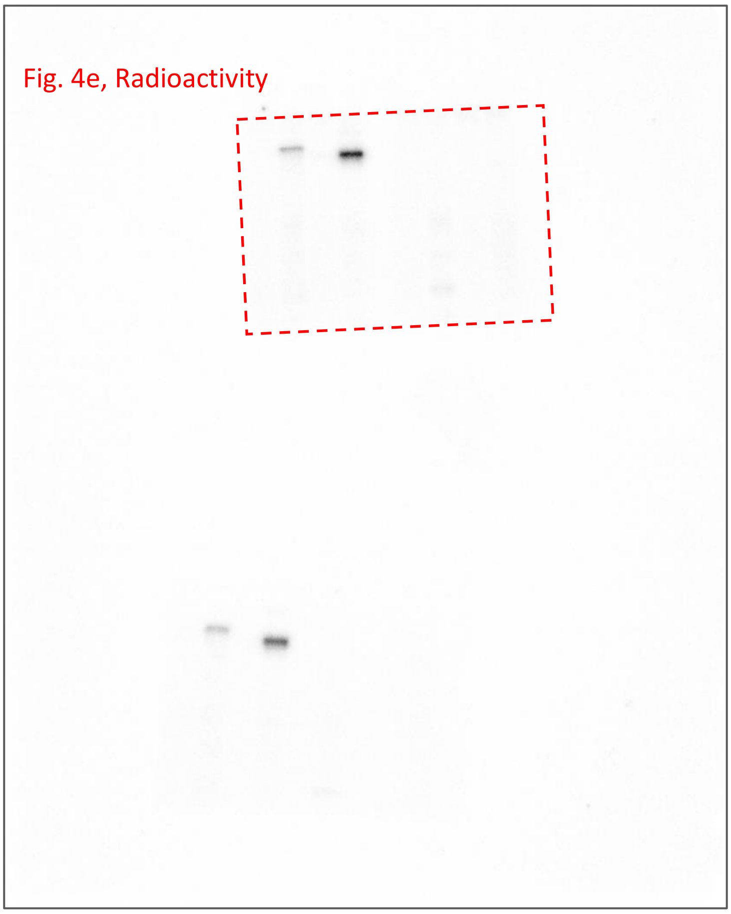

Used in Fig. 4e

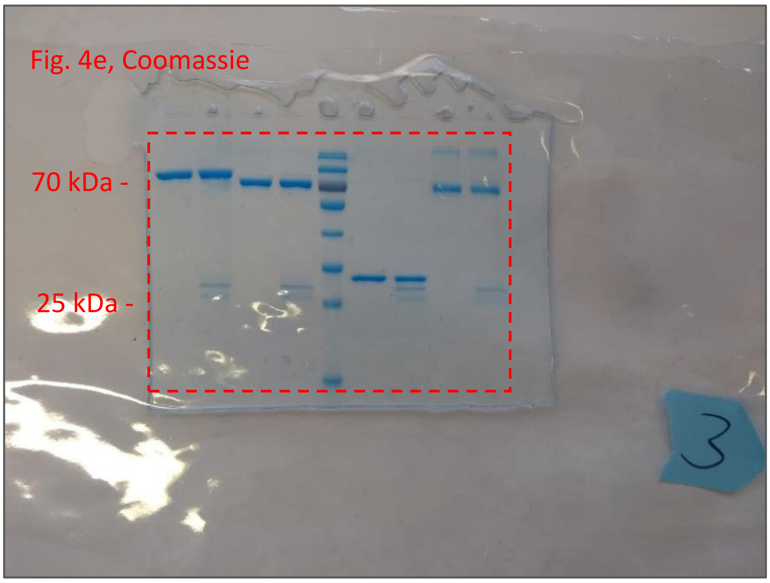

Used in Fig. 4e

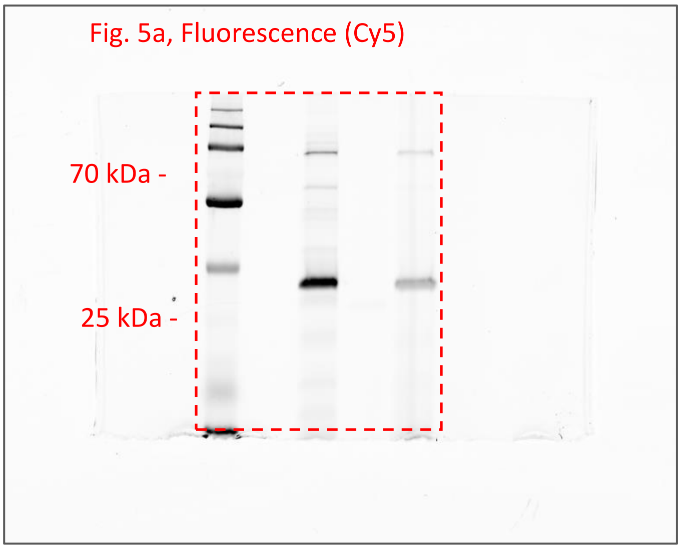

Used in Fig. 5a

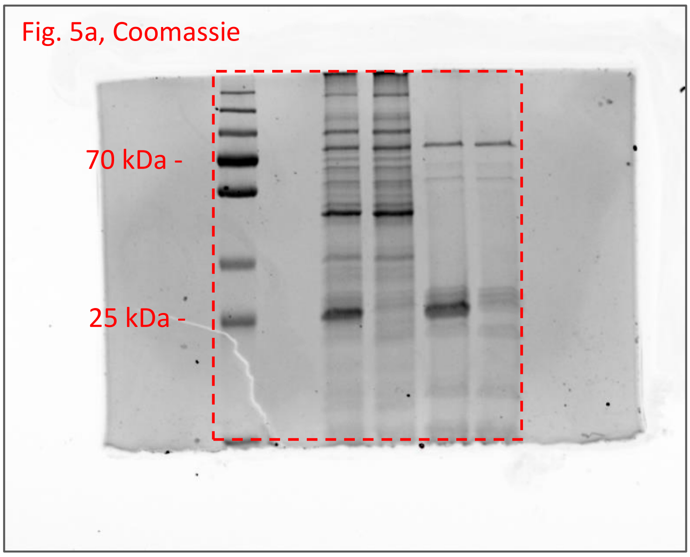

Used in Fig. 5a

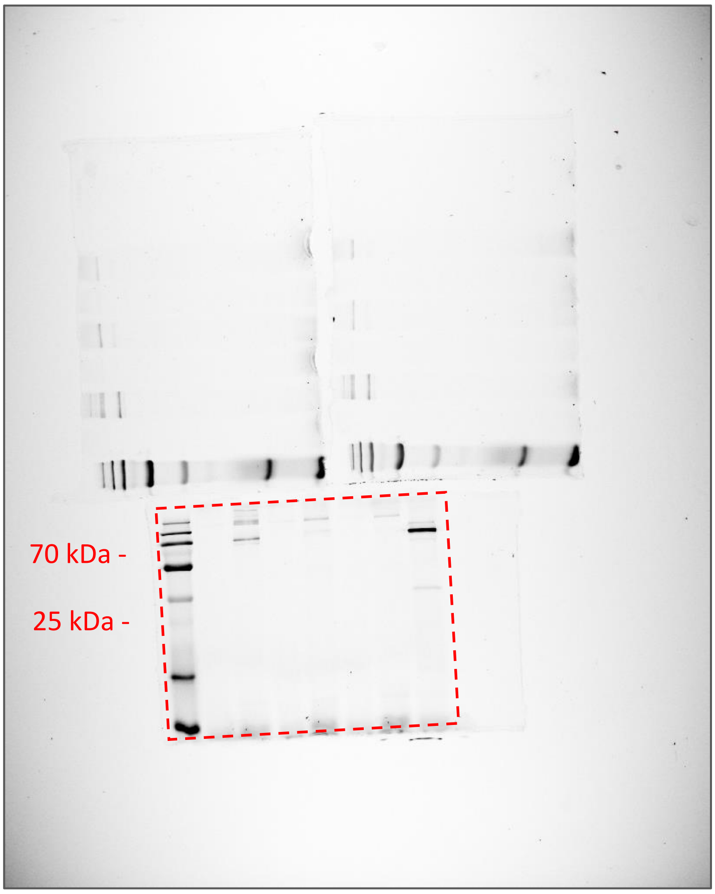

EDF 1a, Fluorescence (Cy5), middle panel

Used in Extended Data Fig. 1a, middle panel

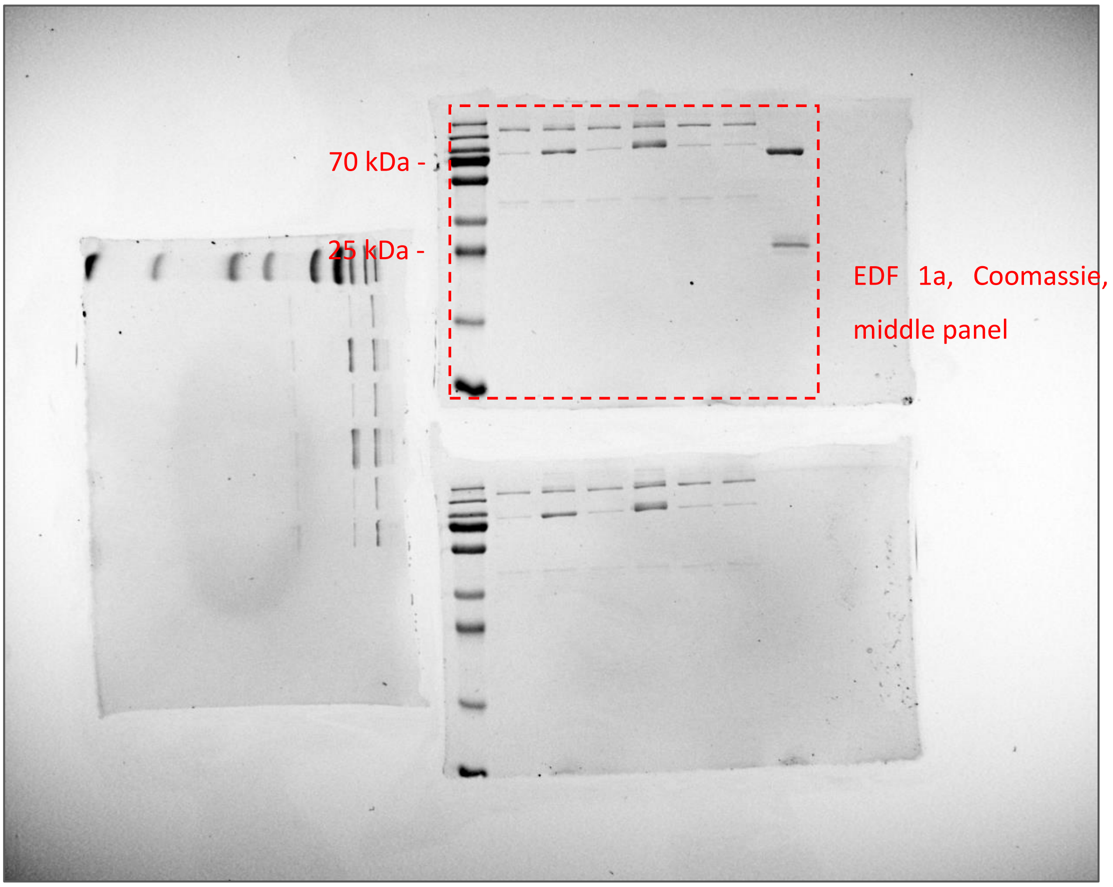

Used in Extended Data Fig. 1a, middle panel

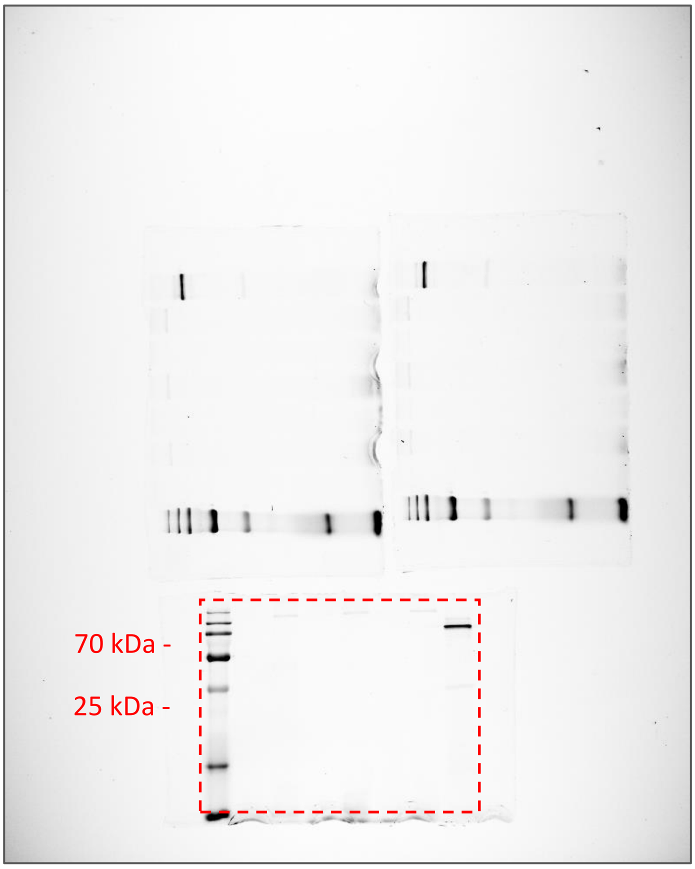

EDF 1a, Fluorescence (Cy5), lower panel

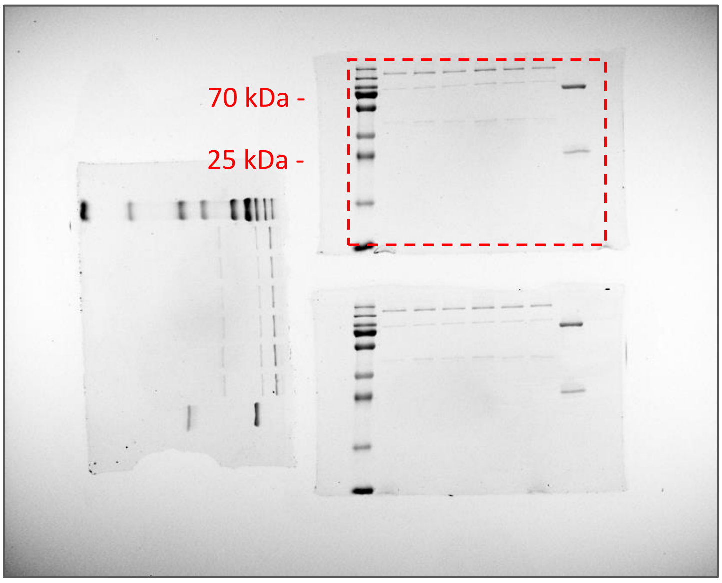

EDF 1a, Coomassie, lower panel

Used in Extended Data Fig. 1a, lower panel

70 kDa -

25 kDa -

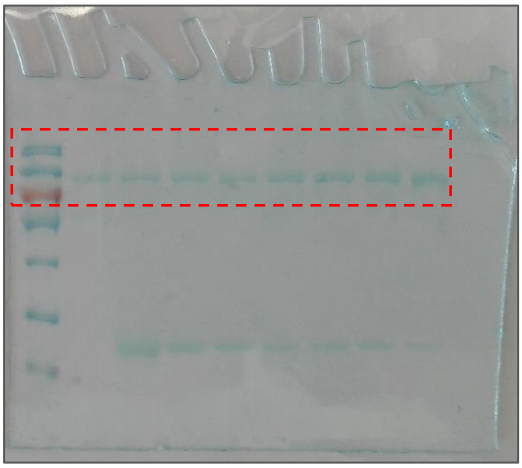

EDF 1d, Coomassie, upper panel

70 kDa -

25 kDa -

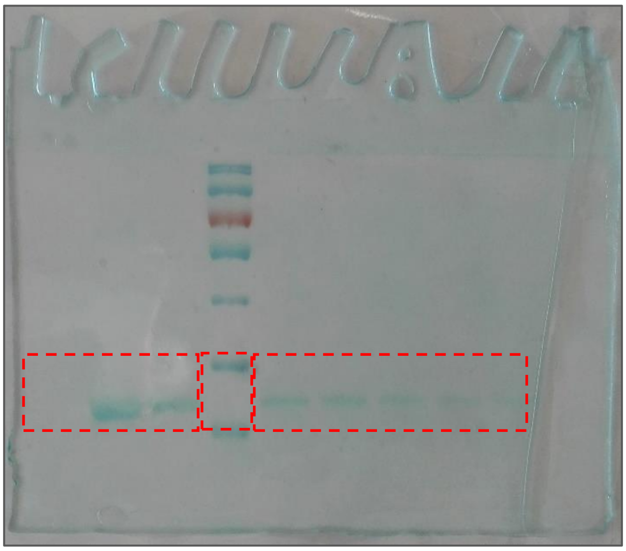

EDF 1d, Coomassie, middle panel

Used in Extended Data Fig. 1d, upper and middle panel

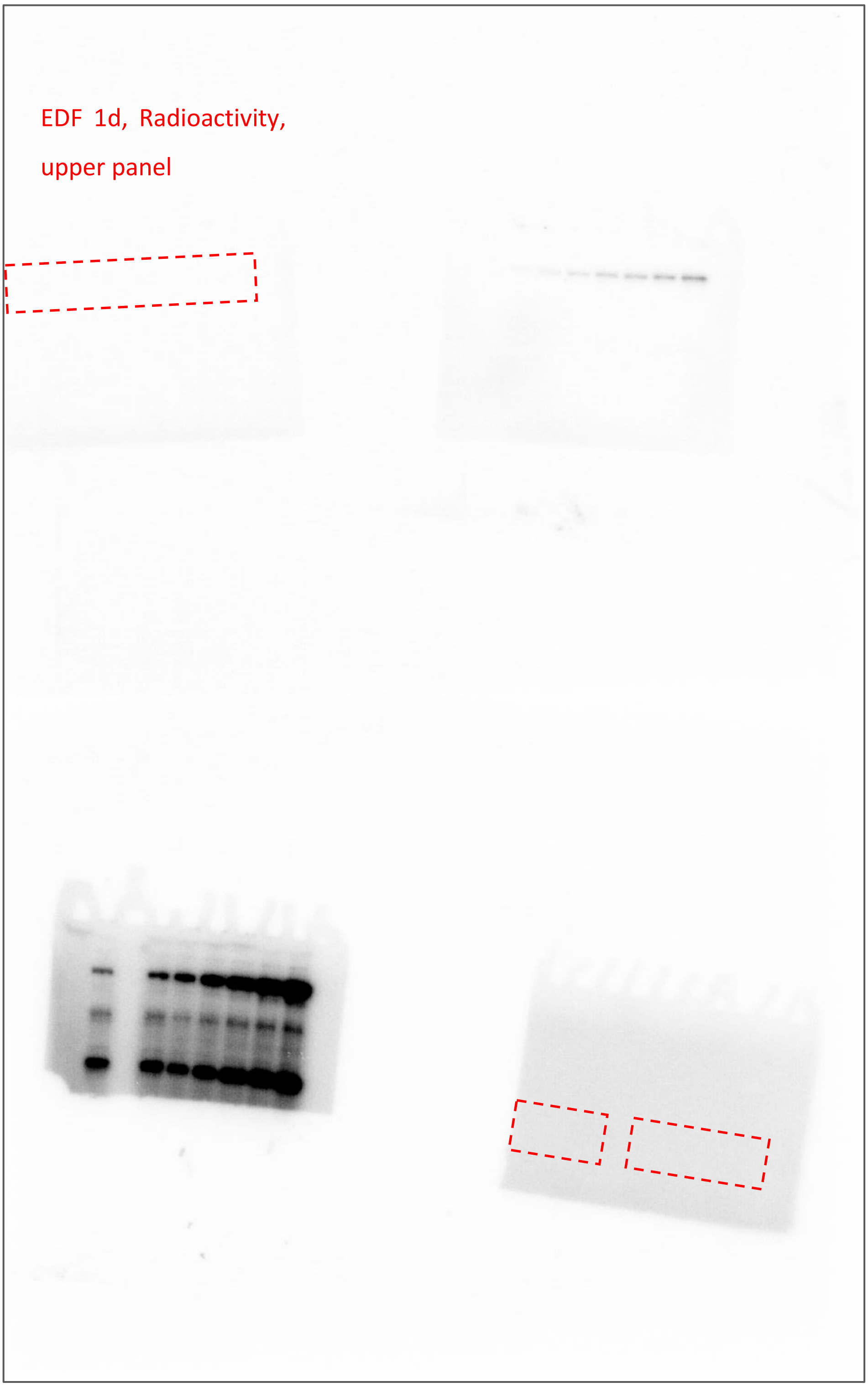

EDF 1d, Radioactivity, middle panel

Used in Extended Data Fig. 1d, upper and middle panel

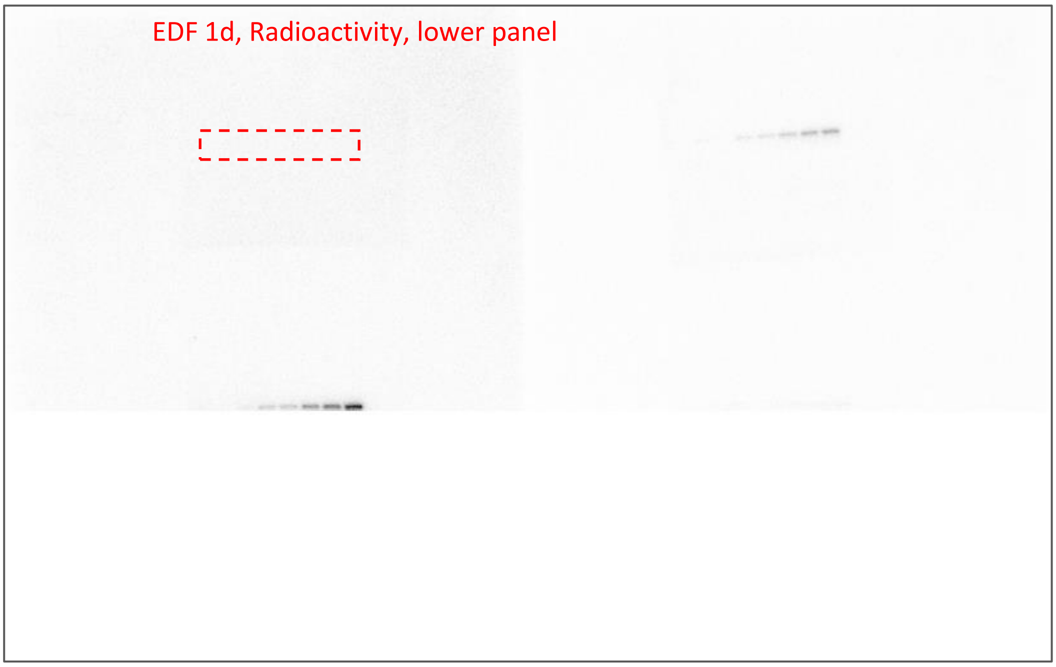

70 kDa -

25 kDa -

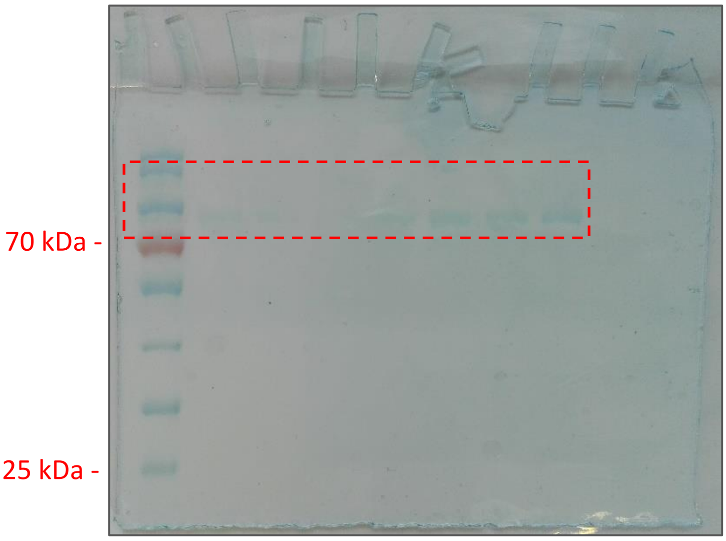

EDF 1d, Coomassie, lower panel

Used in Extended Data Fig. 1d, lower panel

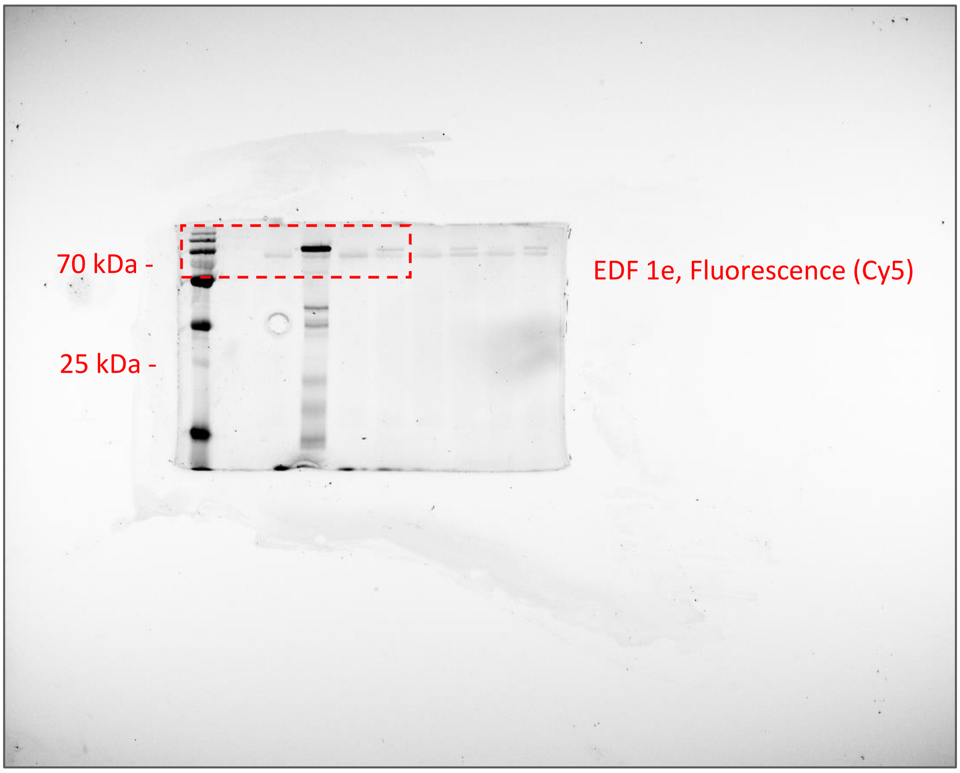

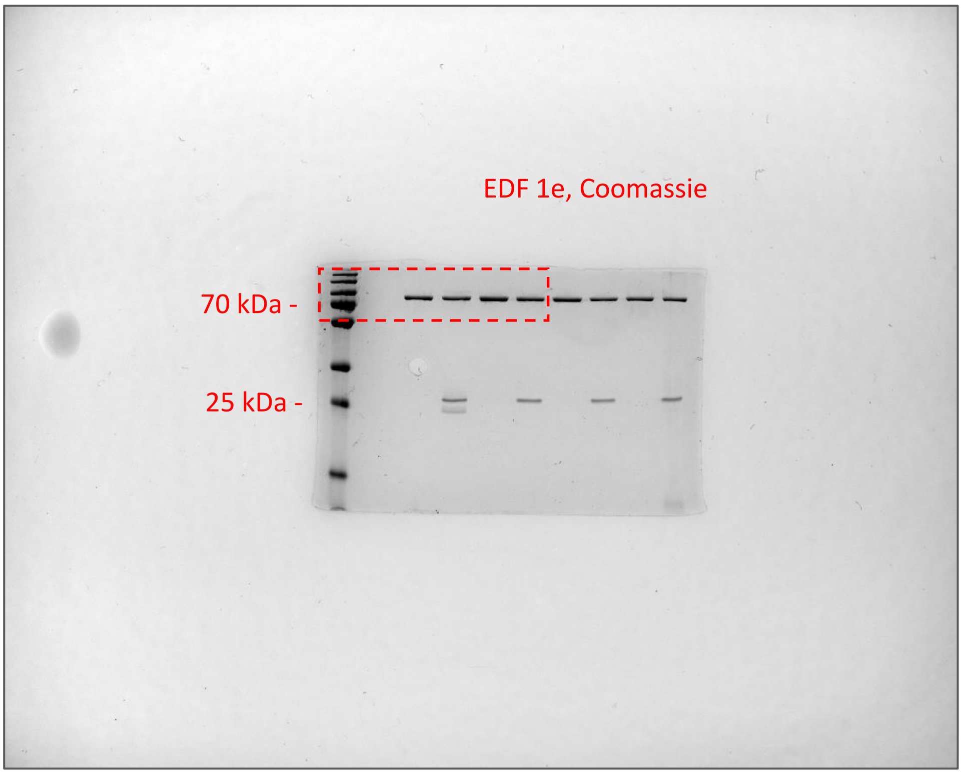

Used in Extended Data Fig. 1e

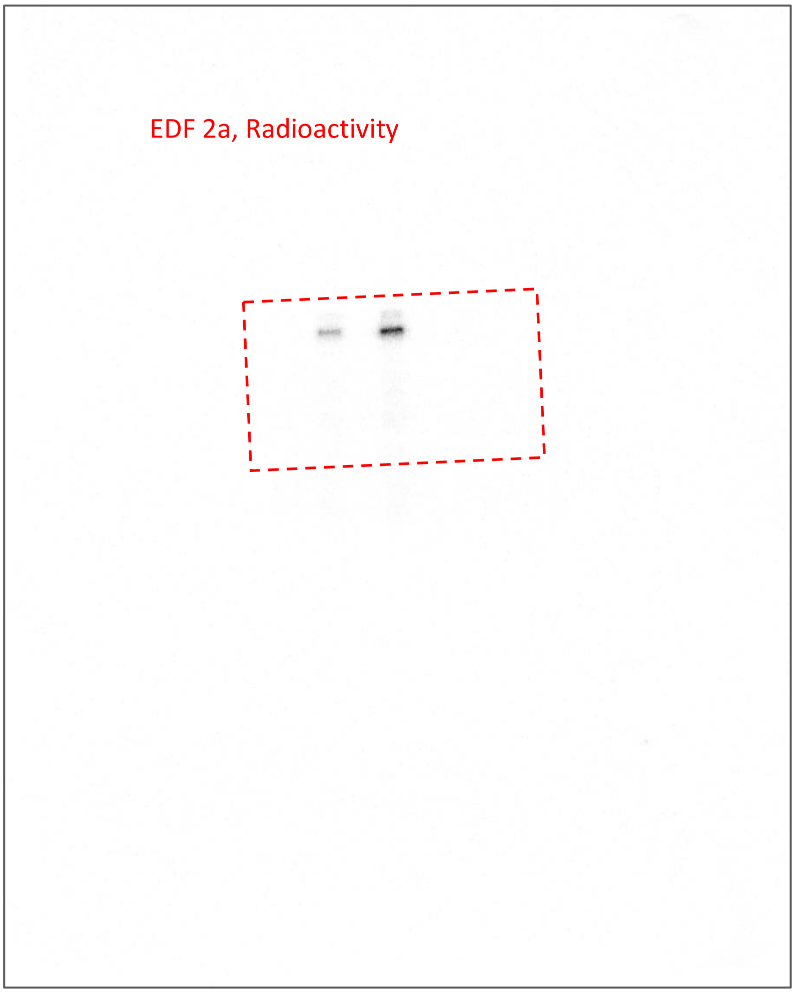

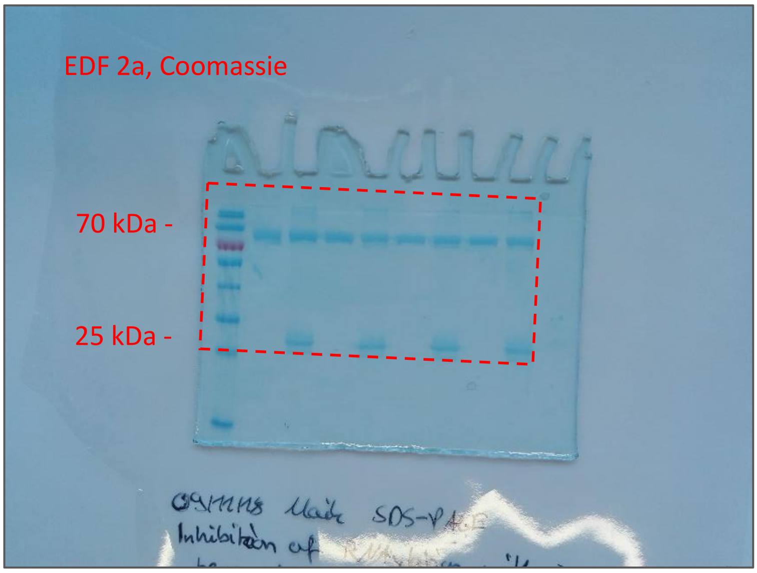

Used in Extended Data Fig. 2a

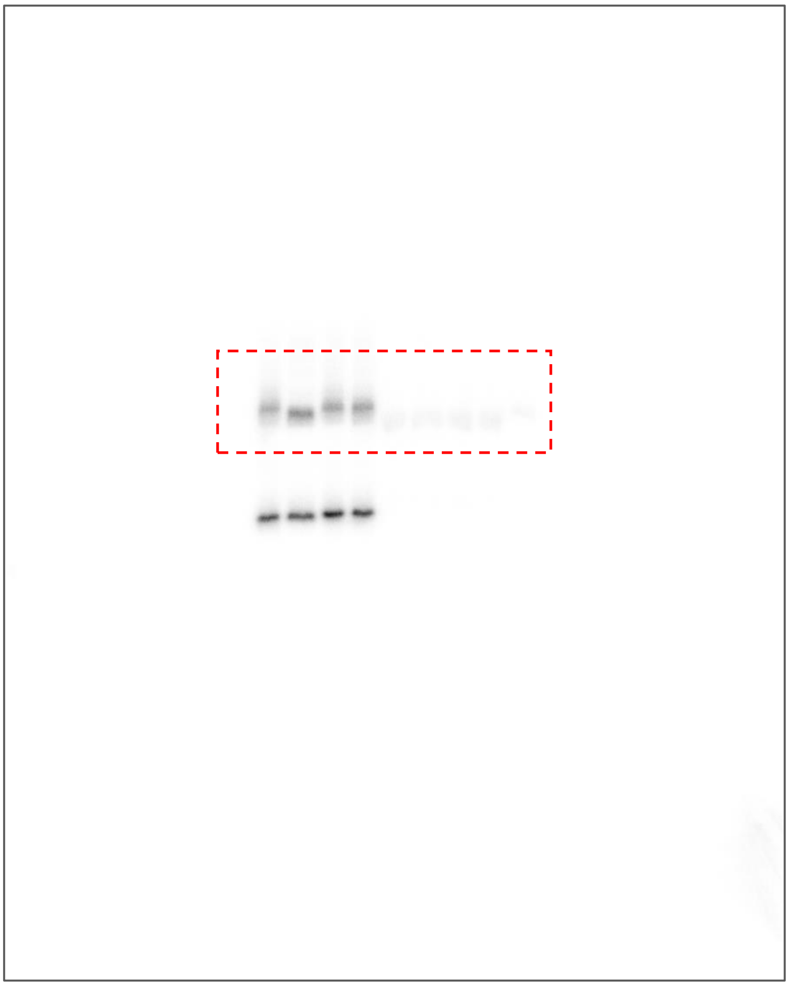

EDF 2b, Radioactivity

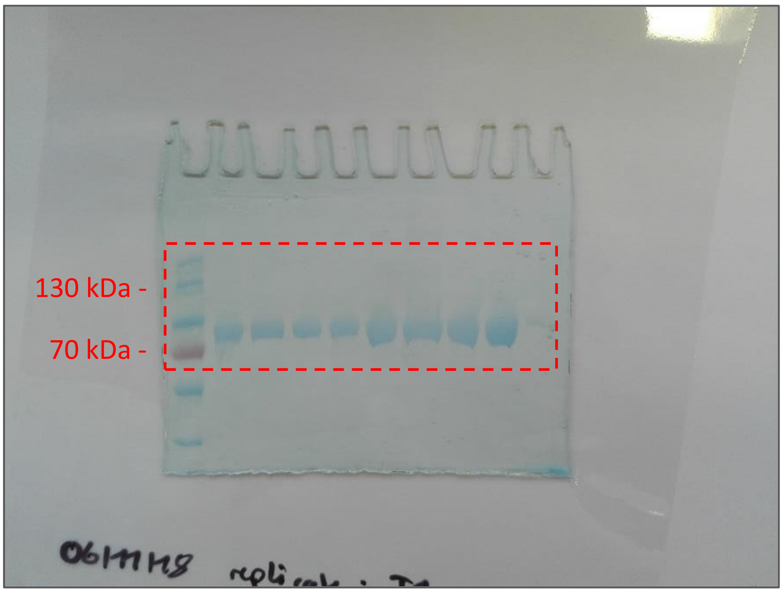

EDF 2b, Coomassie

Used in Extended Data Fig. 2b

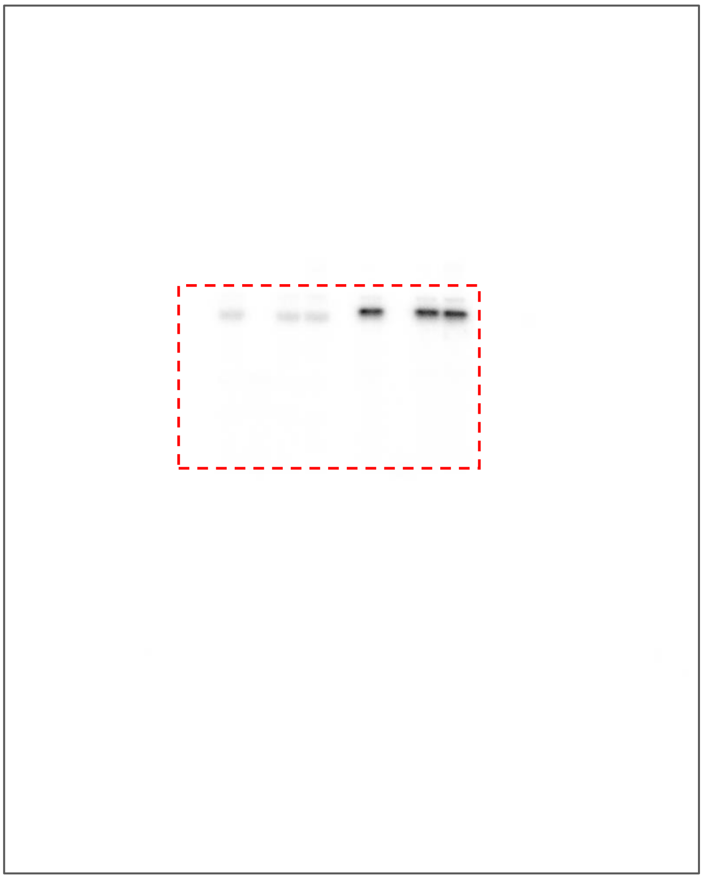

EDF 2c, Radioactivity

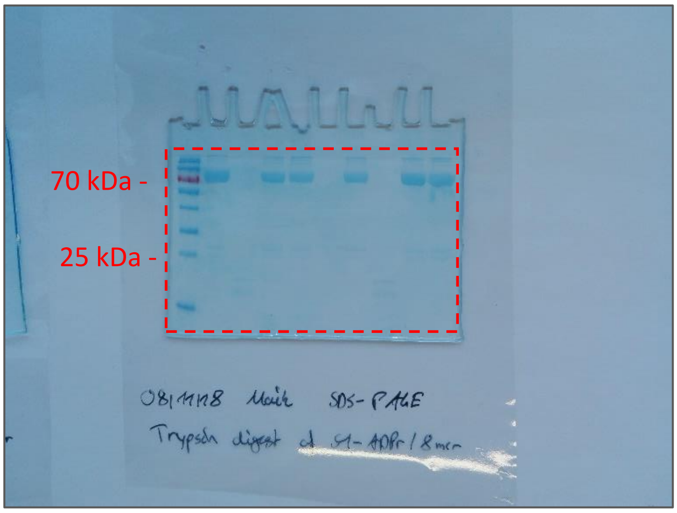

EDF 2c, Coomassie

Used in Extended Data Fig. 2c

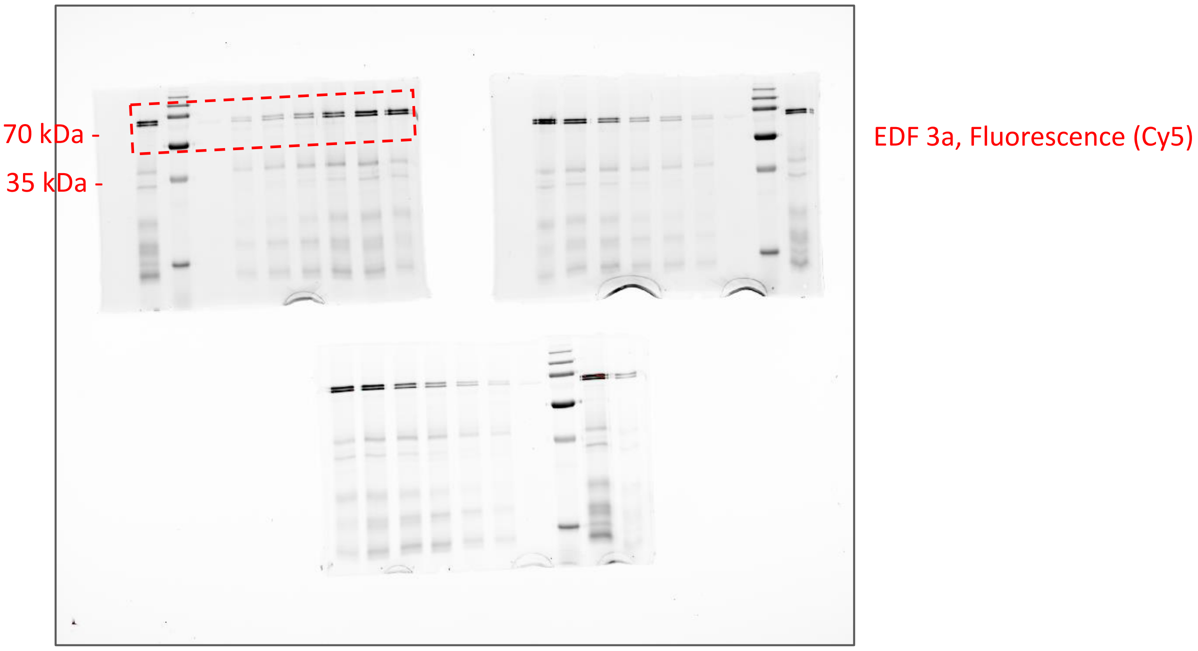

Used in Extended Data Fig. 3a, upper left panel

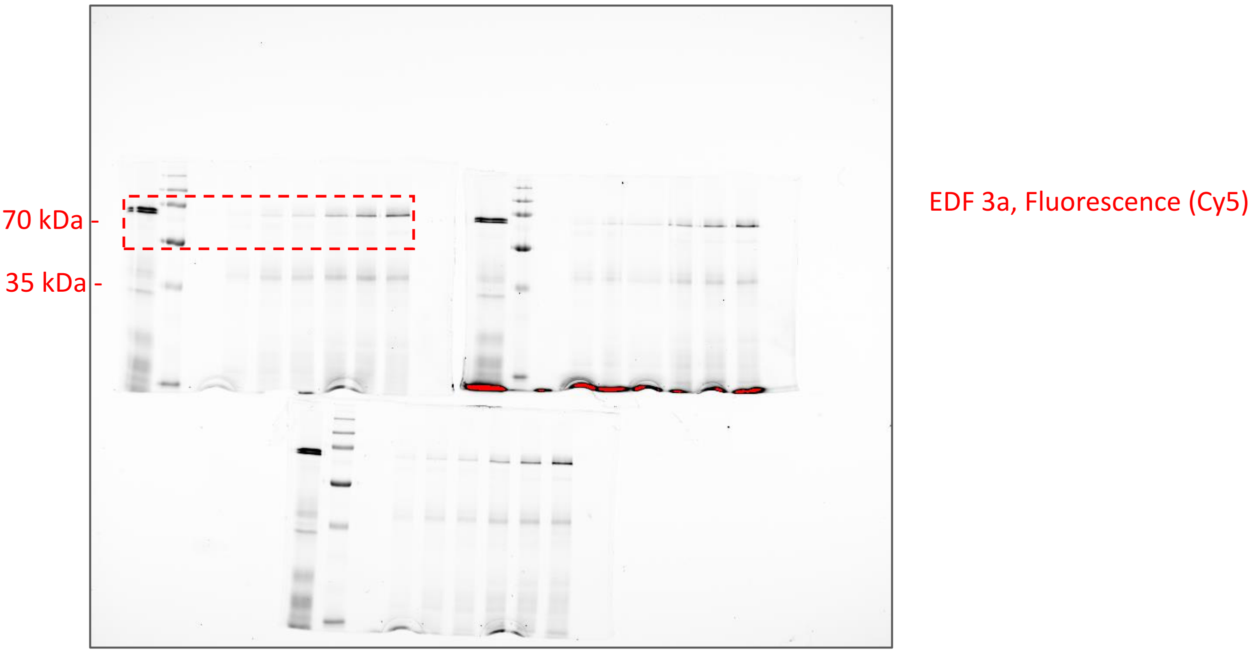

Used in Extended Data Fig. 3a, upper right panel

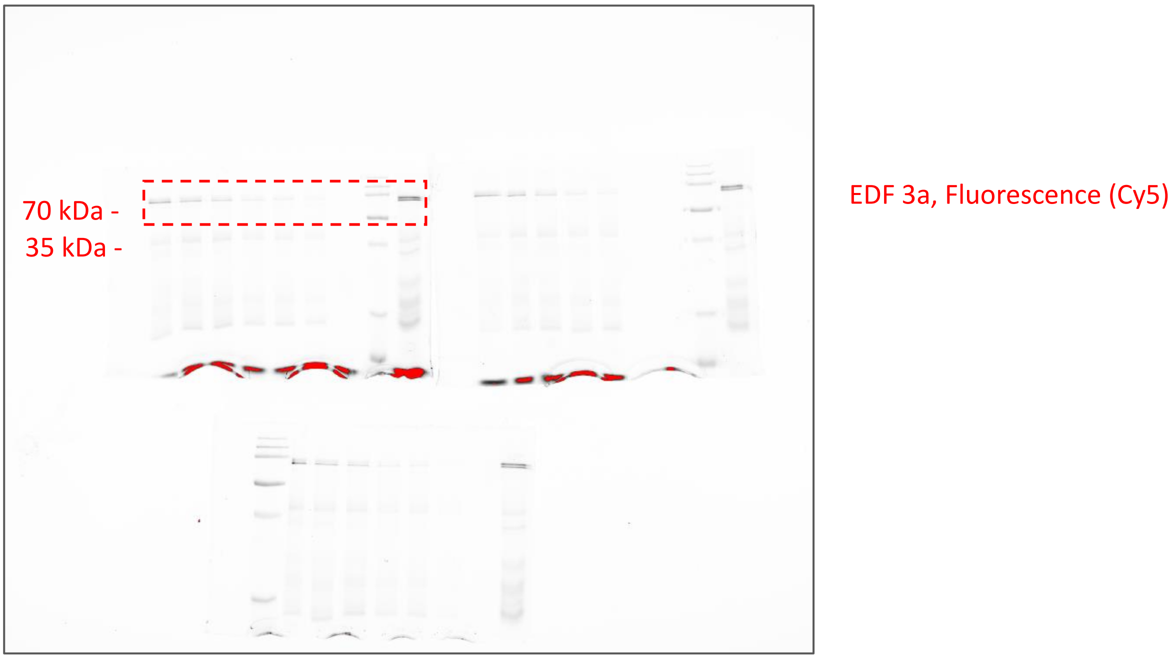

Used in Extended Data Fig. 3a, lower left panel

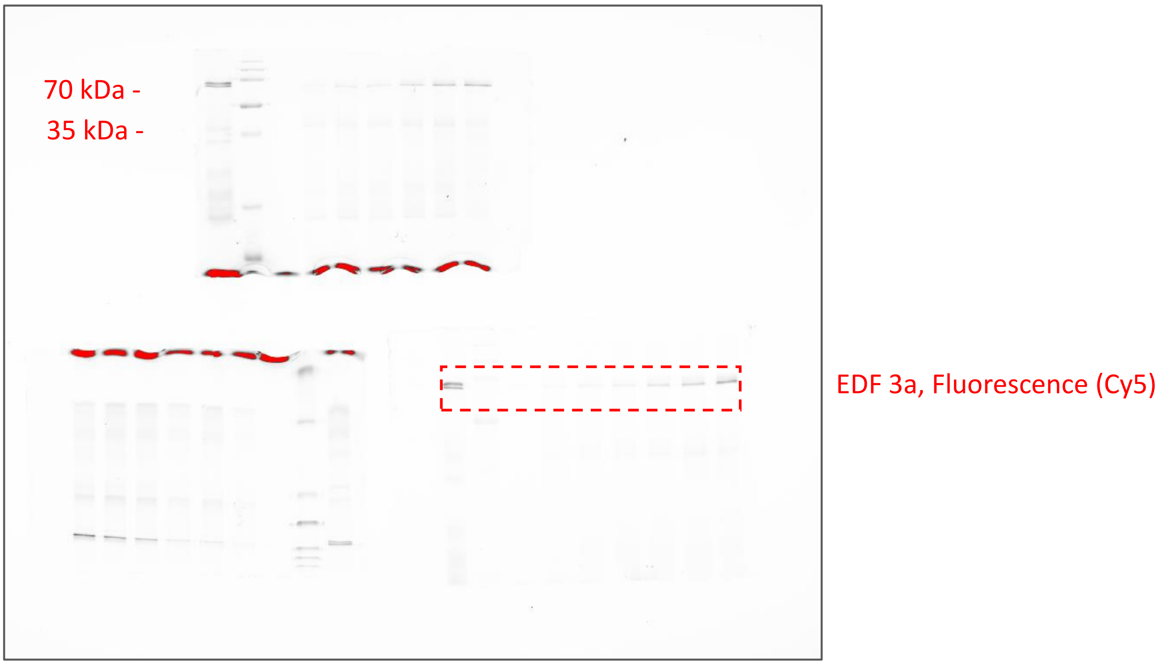

Used in Extended Data Fig. 3a, lower right panel

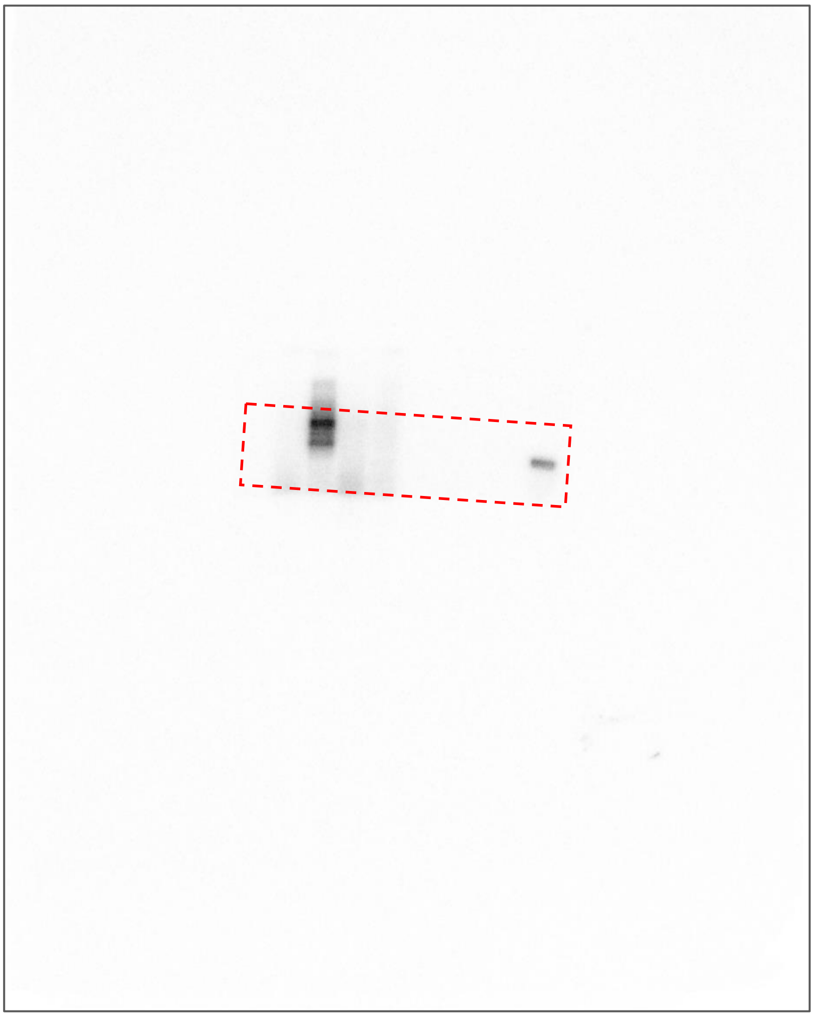

EDF 3b, Radioactivity

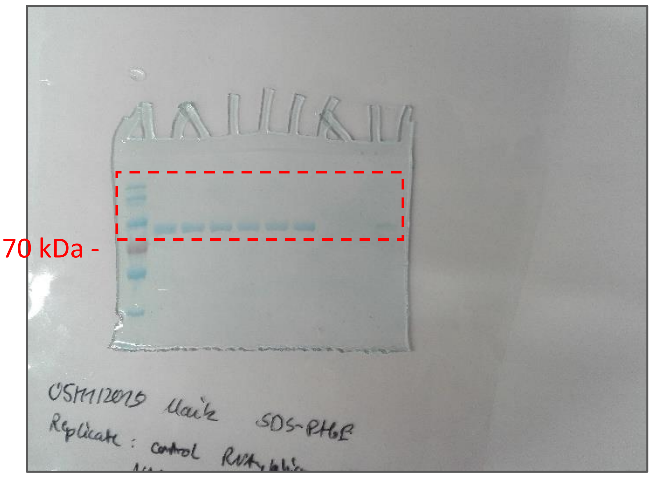

70 kDa -

EDF 3b, Coomassie

Used in Extended Data Fig. 3b

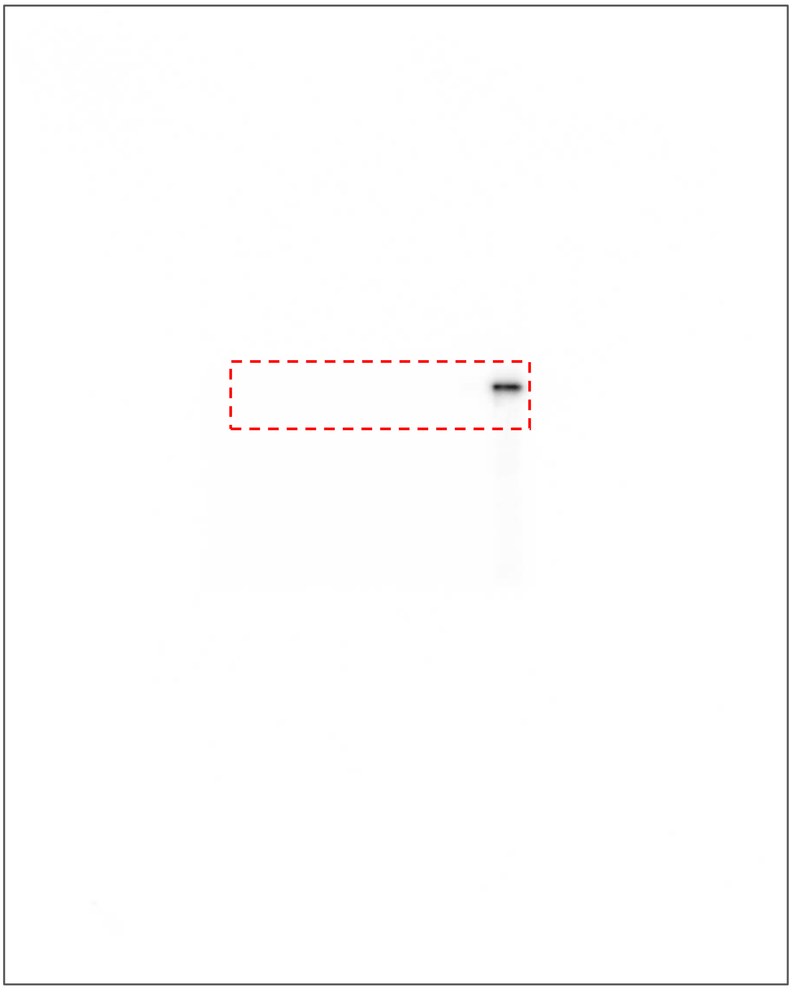

EDF 3c, Radioactivity

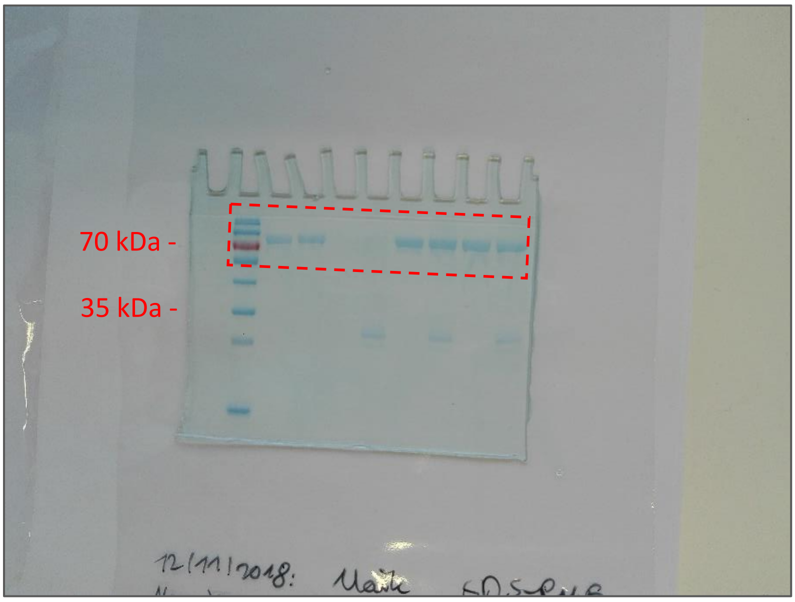

EDF 3c, Coomassie

Used in Extended Data Fig. 3c

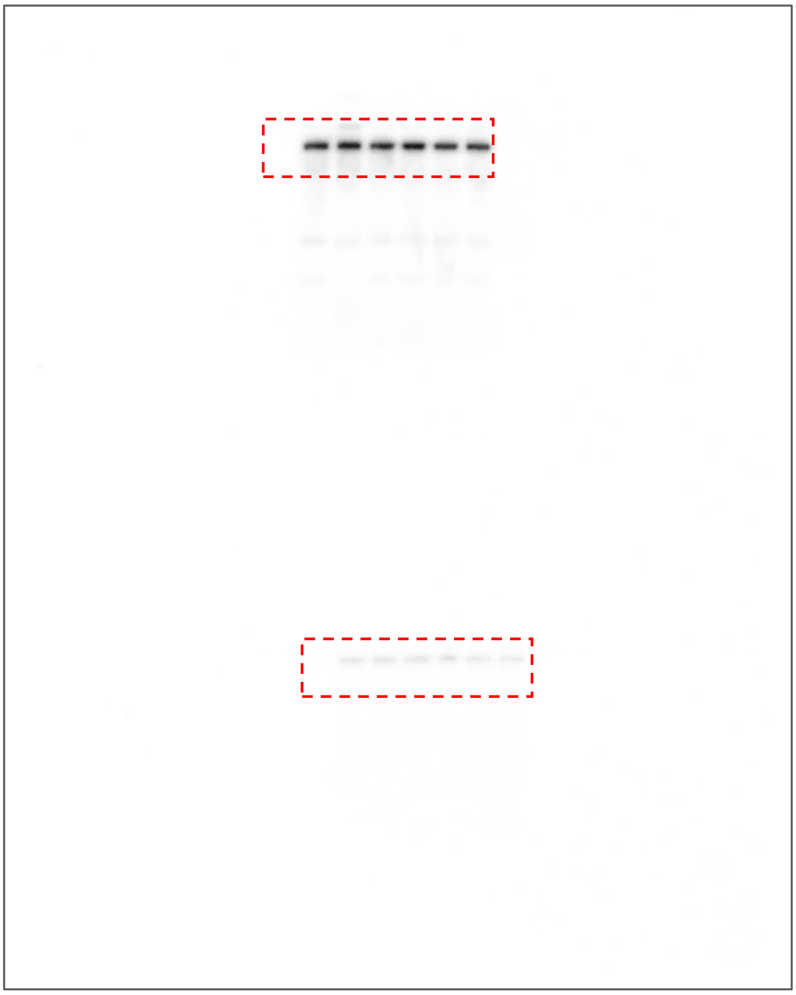

EDF 4a, Radioactivity, ADP-ribosylated rS1

EDF 4a, Radioactivity, RNAylated rS1

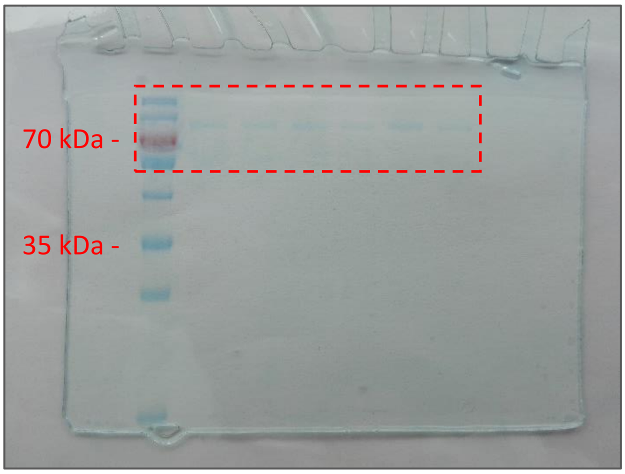

EDF 4a, Coomassie, ADP-ribosylated rS1

Used in Extended Data Fig. 4a

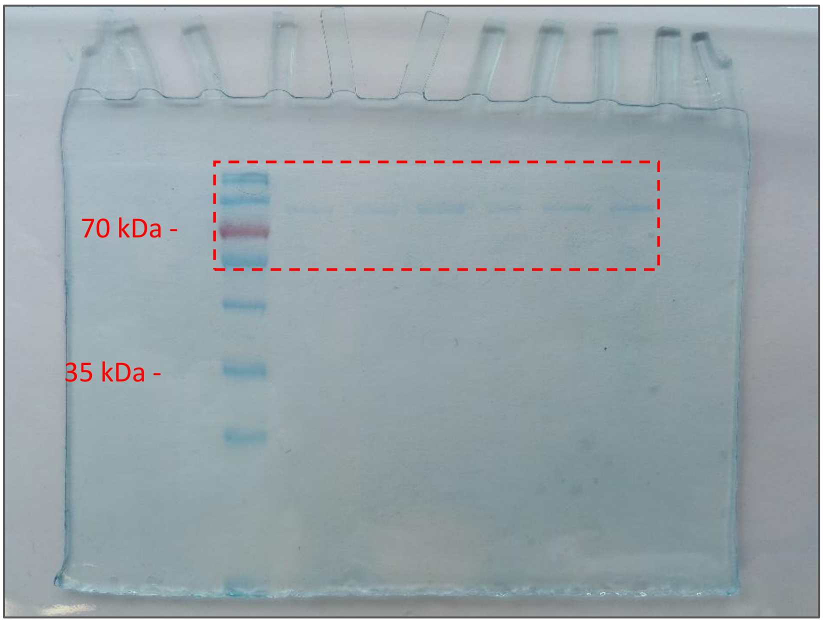

EDF 4a, Coomassie, RNAylated rS1

Used in Extended Data Fig. 4a

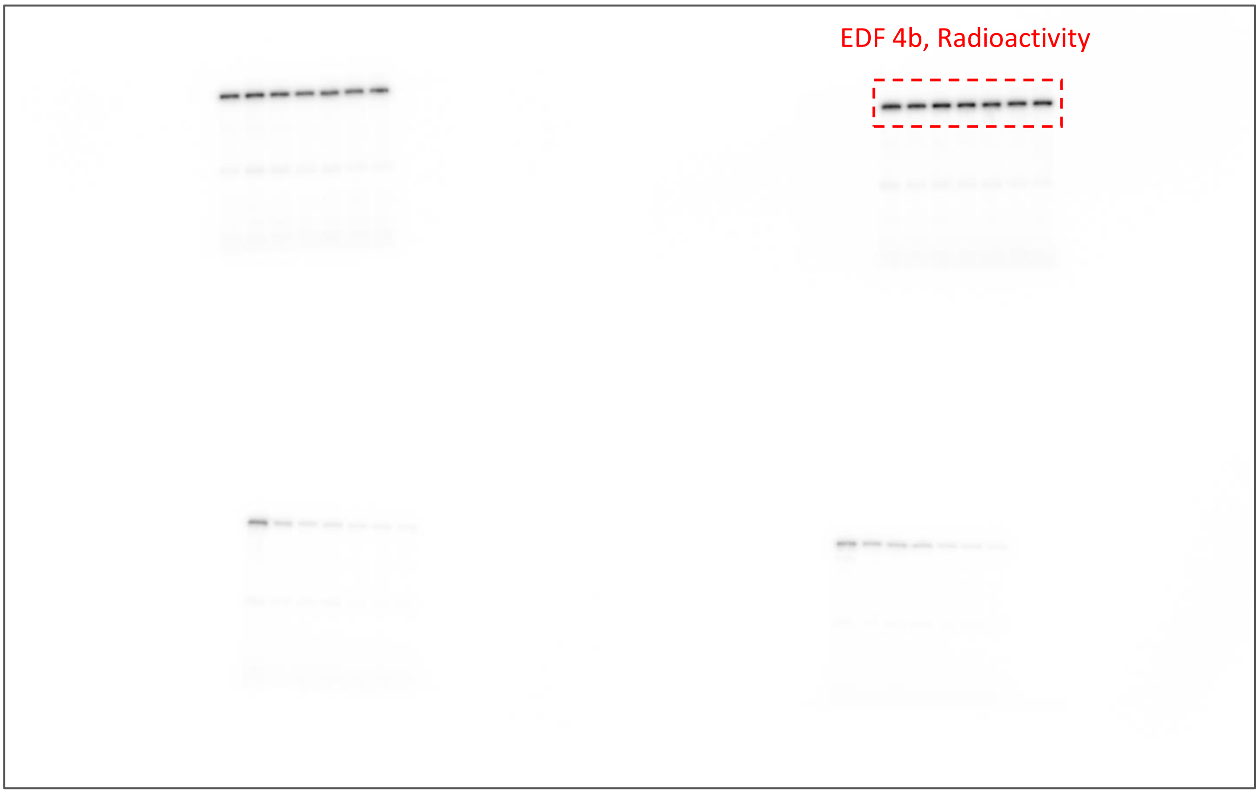

Used in Extended Data Fig. 4b

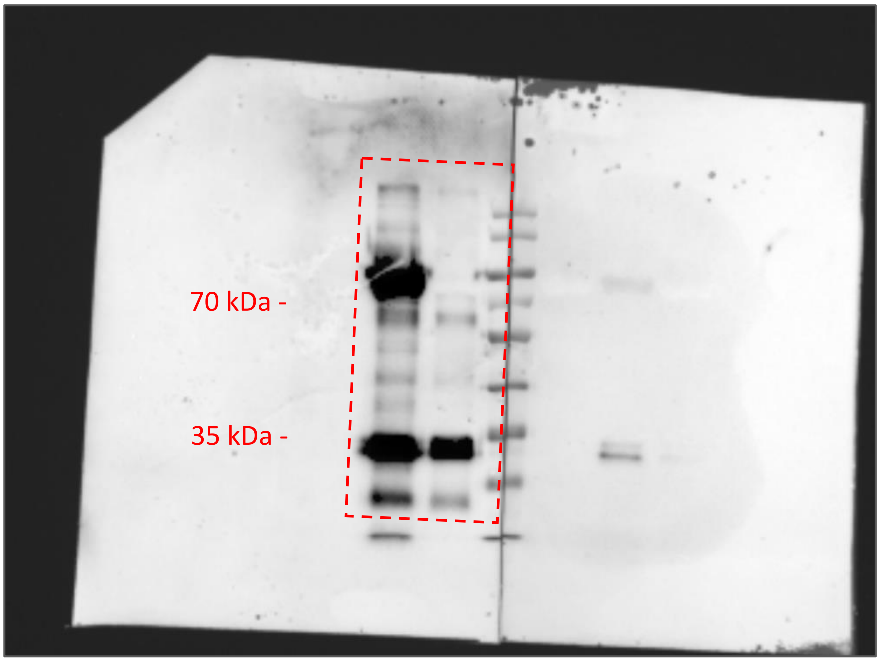

Used in Extended Data Fig. 6a

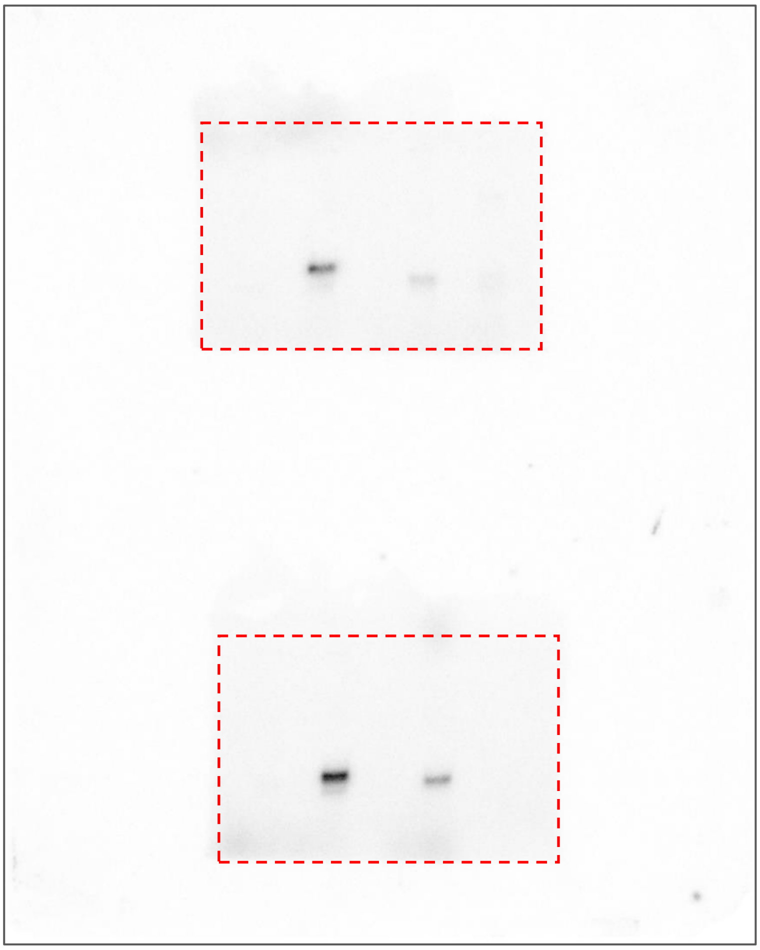

EDF 7b, Radioactivity, upper panel

EDF 7b, Radioactivity, lower panel

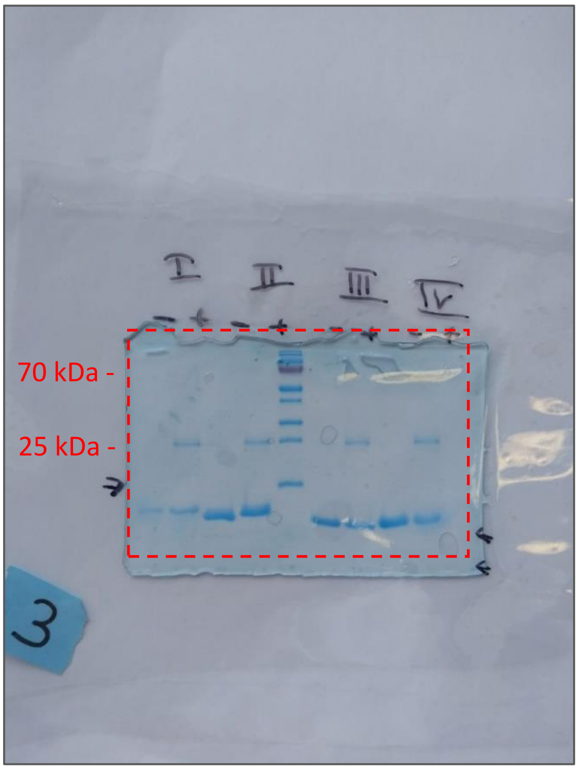

EDF 7b, Coomassie, upper panel

Used in Extended Data Fig. 7b

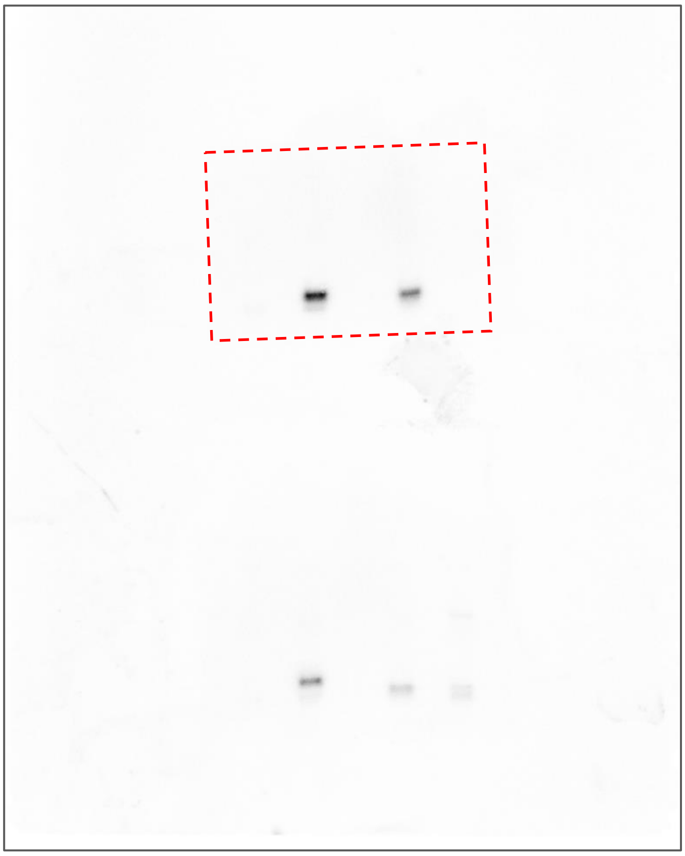

EDF 7b, Radioactivity, lower panel

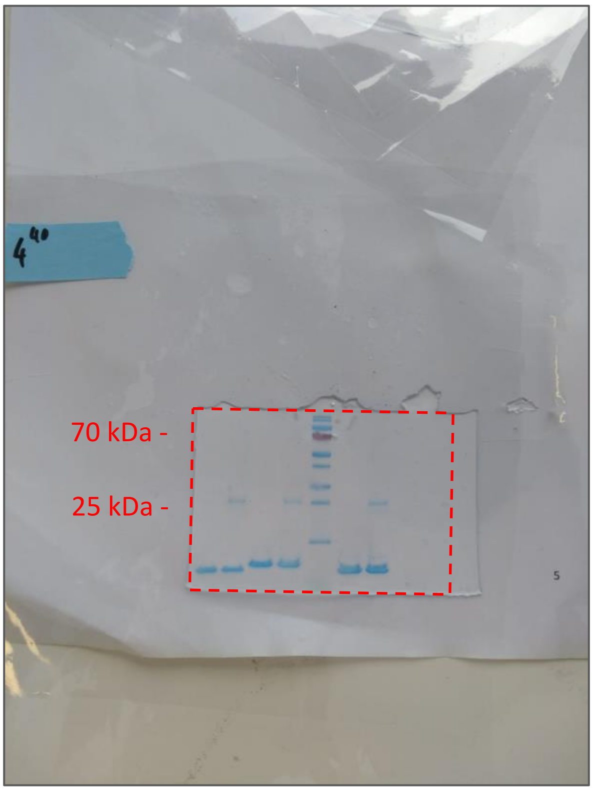

EDF 7b, Coomassie, lower panel

Used in Extended Data Fig. 7b

Used in Extended Data Fig. 8a

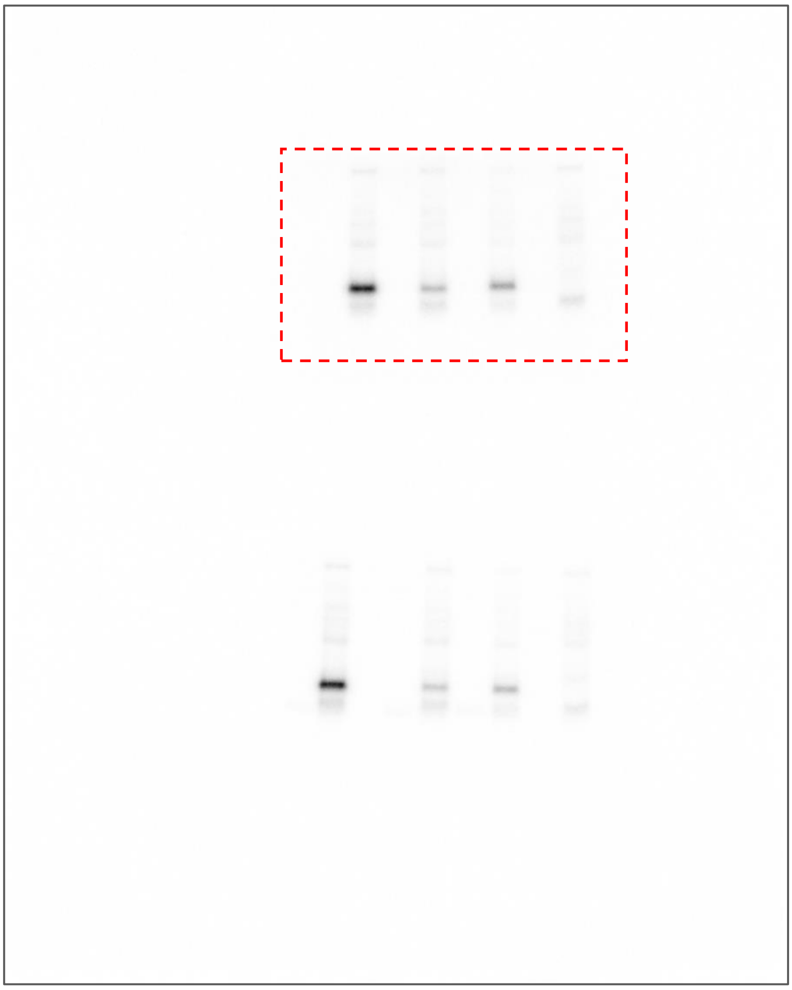

EDF 8a, Radioactivity

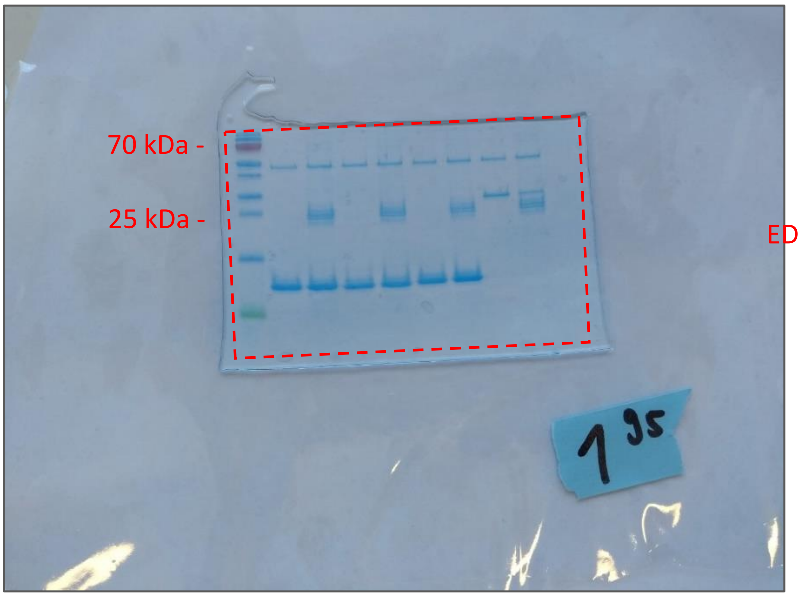

EDF 8a, Coomassie

Used in Extended Data Fig. 8a

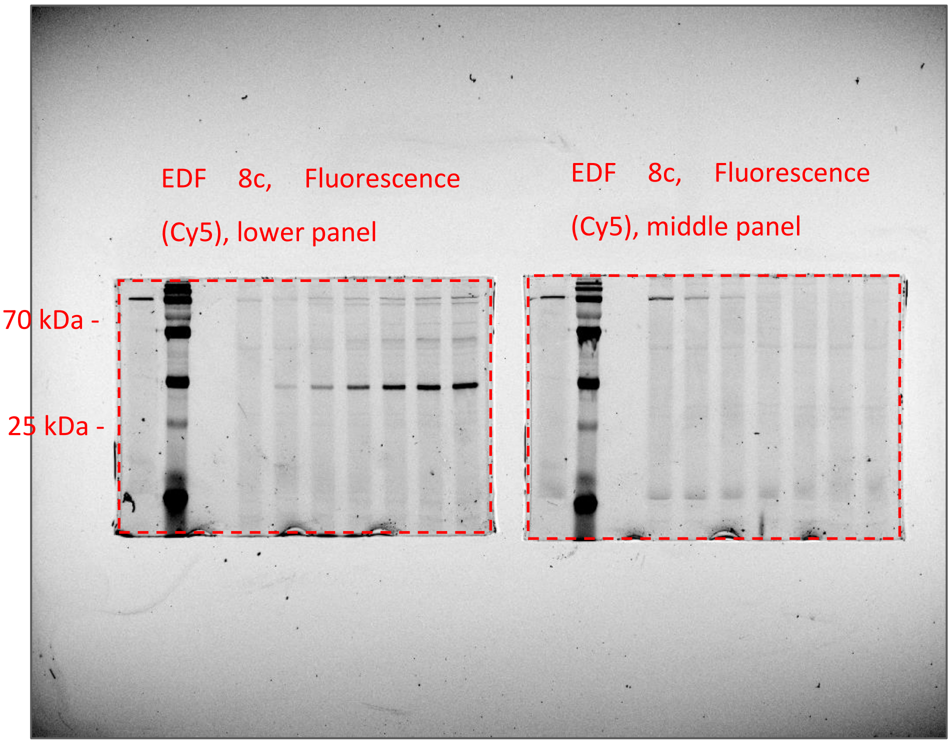

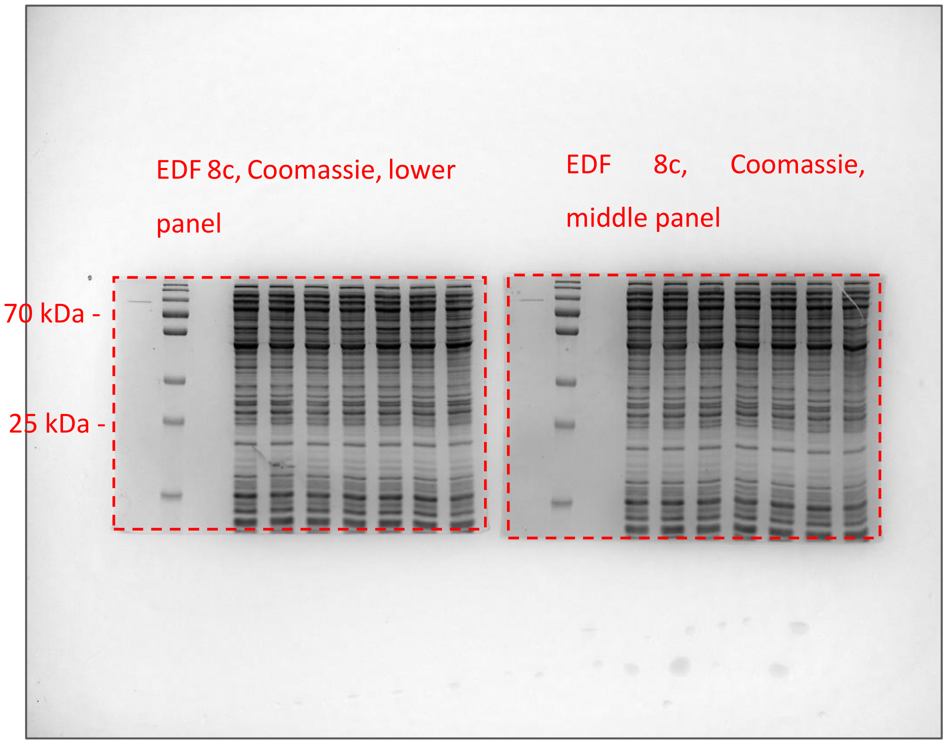

Used in Extended Data Fig. 8c

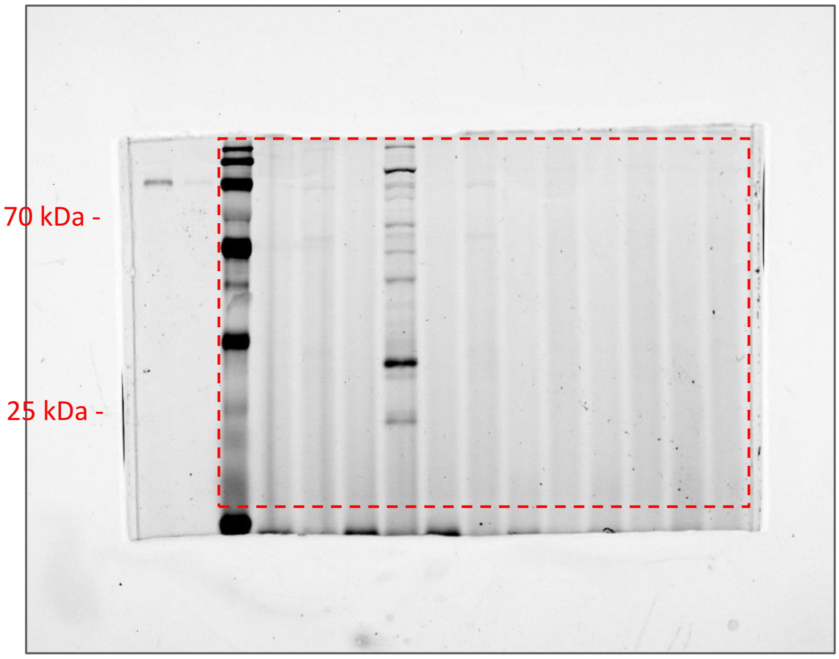

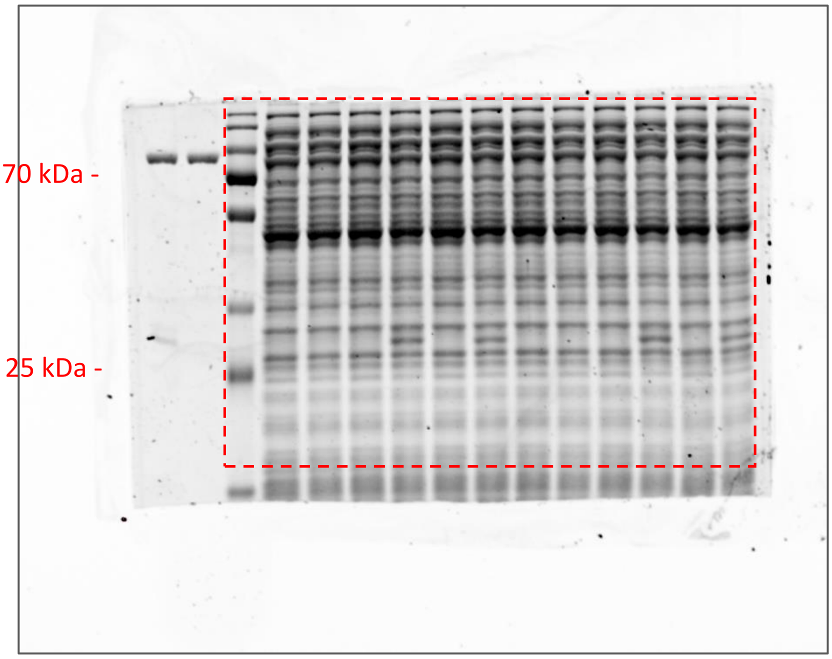

Used in Extended Data Fig. 9a

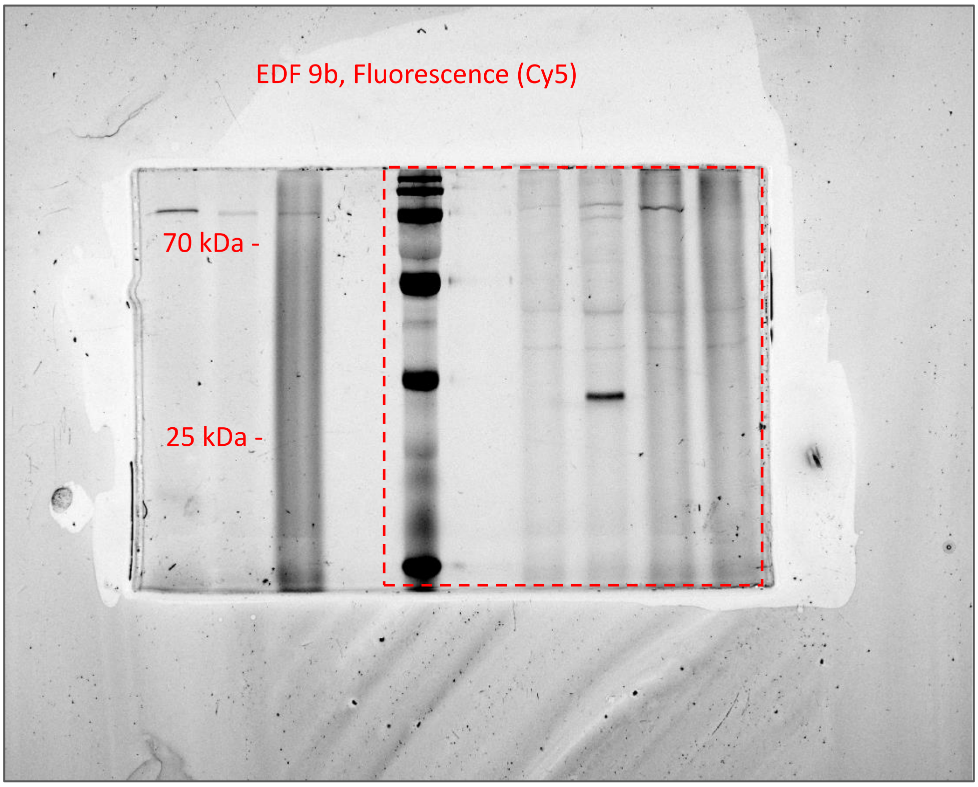

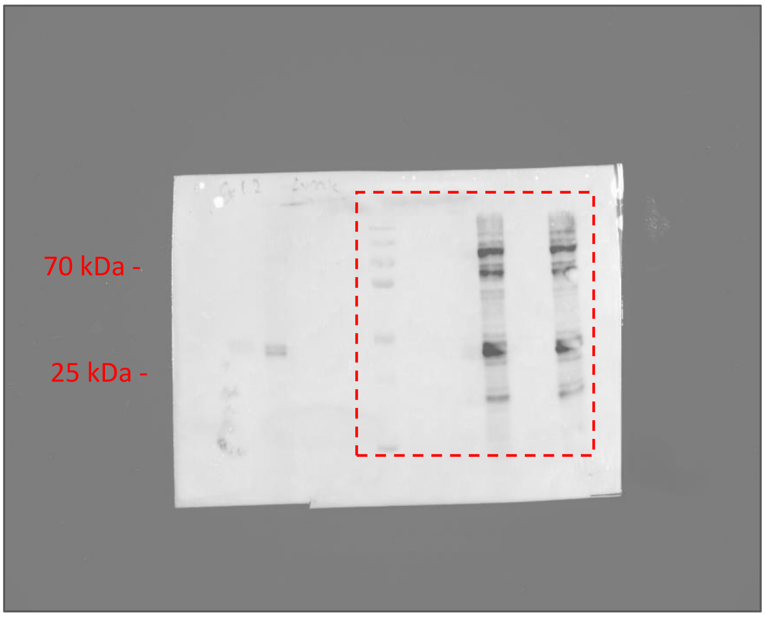

EDF 9b, pan-ADPr Western blot merged with Colorimetric scan

Used in Extended Data Fig. 9b

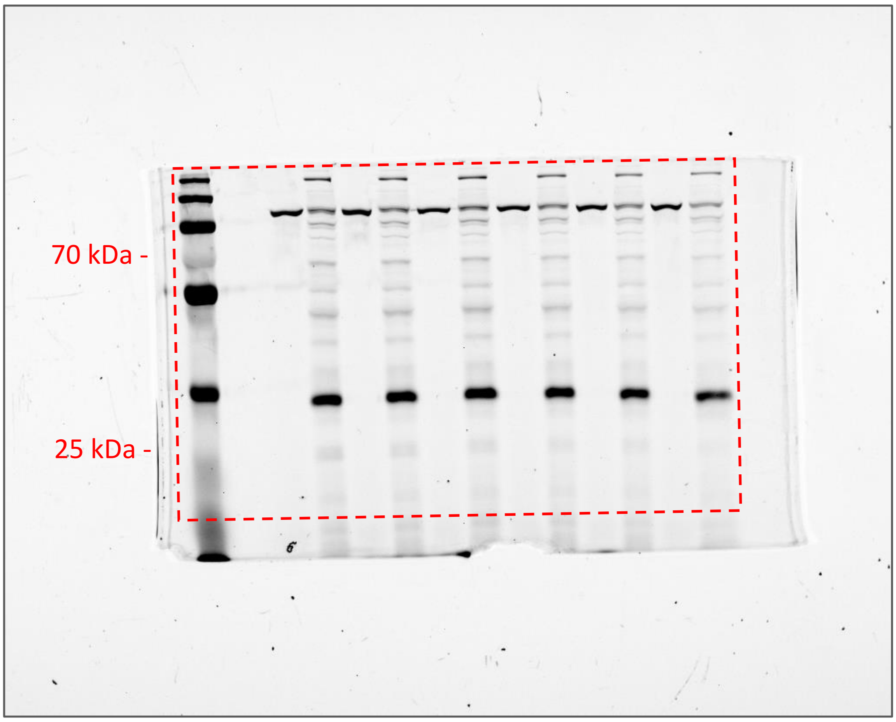

EDF 9c, Fluorescence (Cy5)

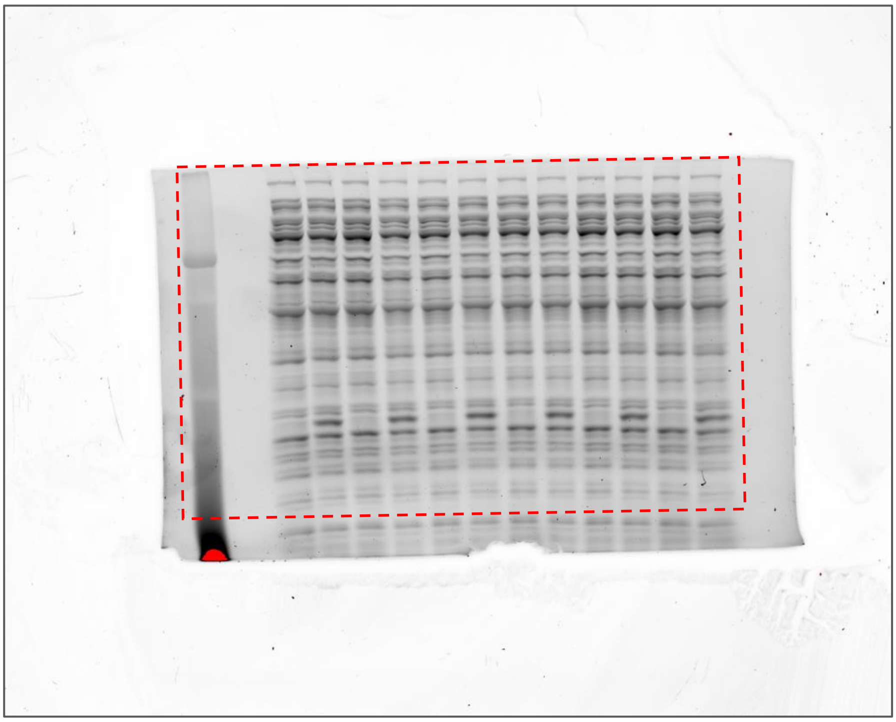

EDF 9c, TCE stain

Used in Extended Data Fig. 9c

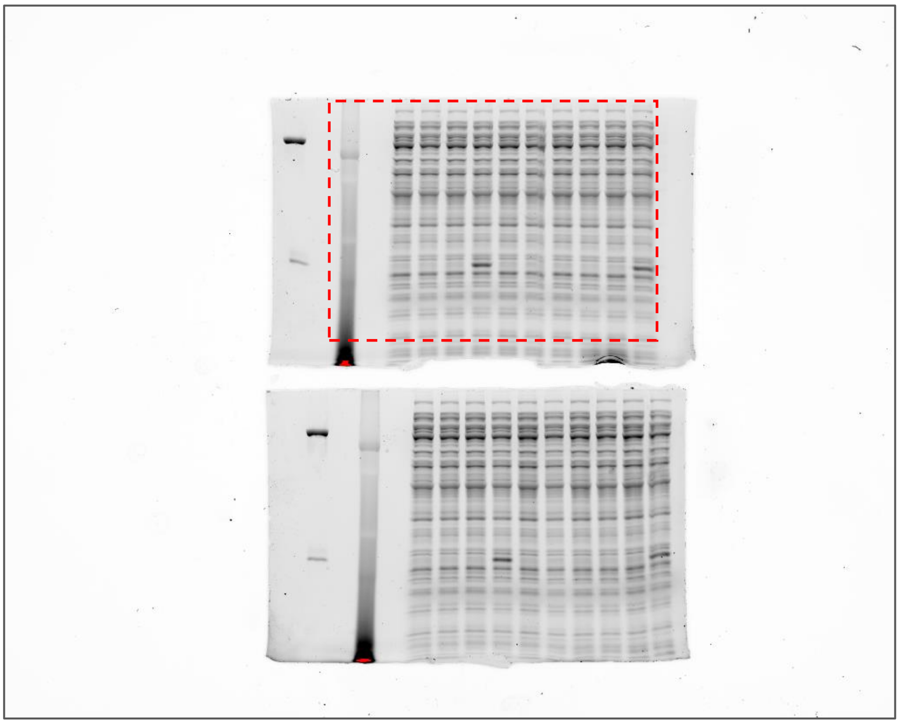

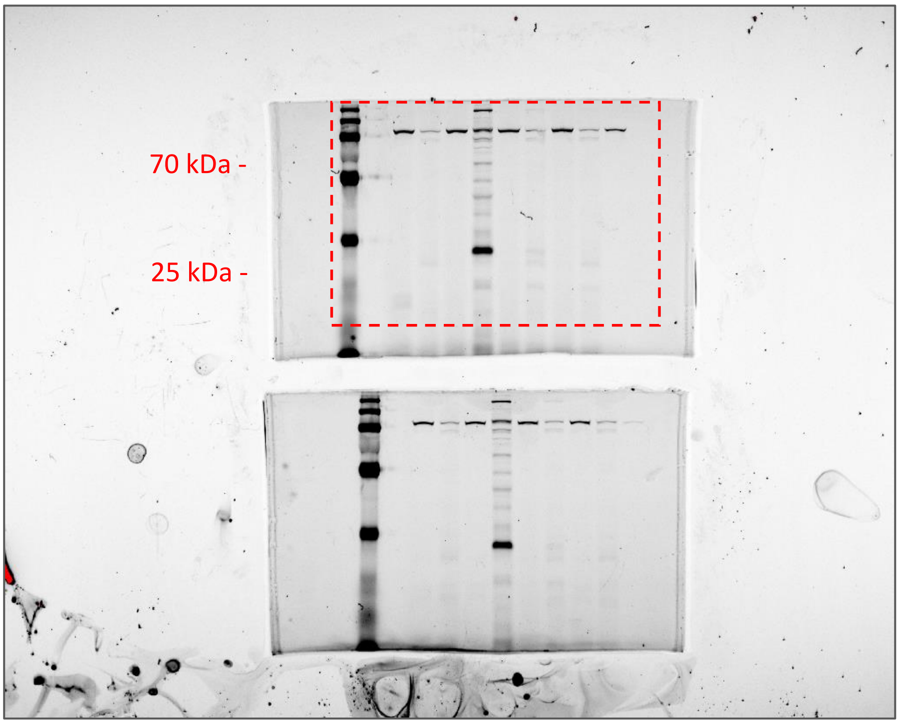

Used in Extended Data Fig. 9d

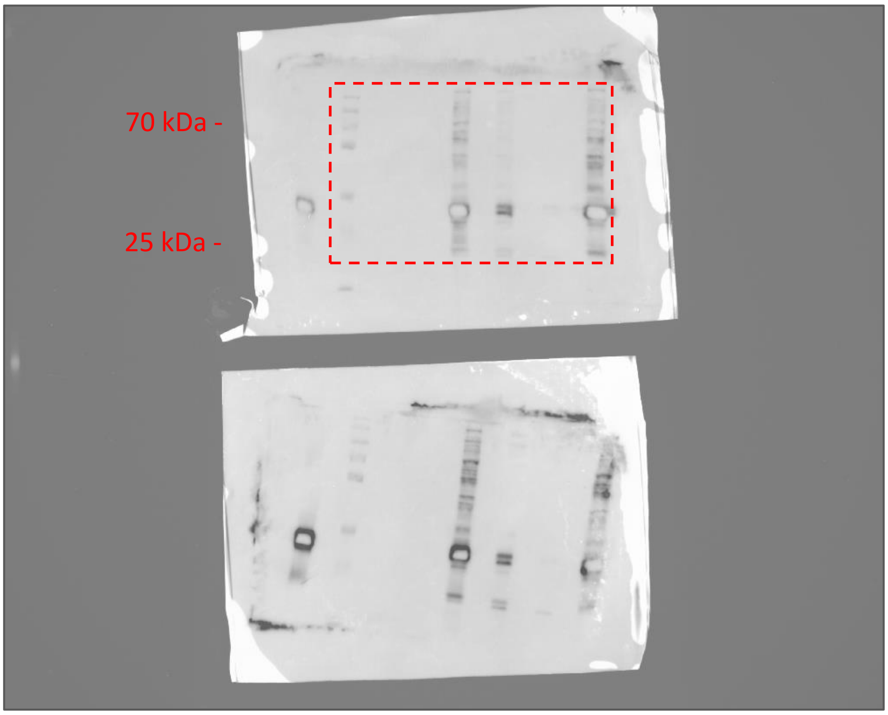

EDF 9d, pan-ADPr Western blot merged with Colorimetric scan

Used in Extended Data Fig. 9d

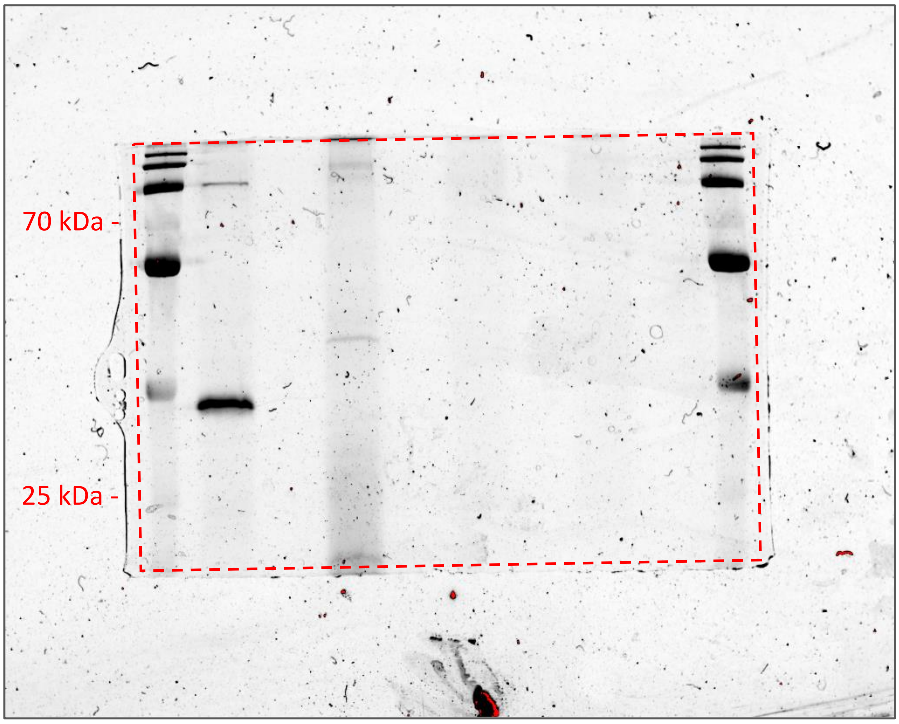

EDF 10a, Fluorescence (Cy5)

Used in Extended Data Fig.10a

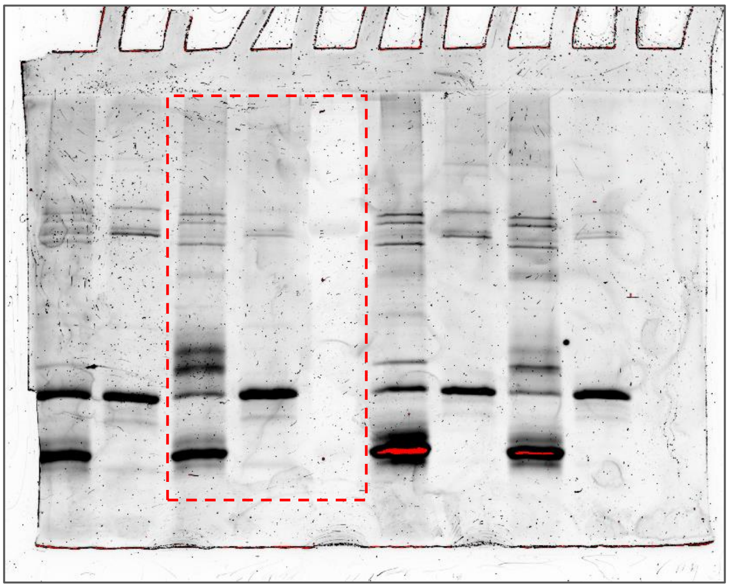

EDF 10c, Flamingo stain

35 kDa -

25 kDa -

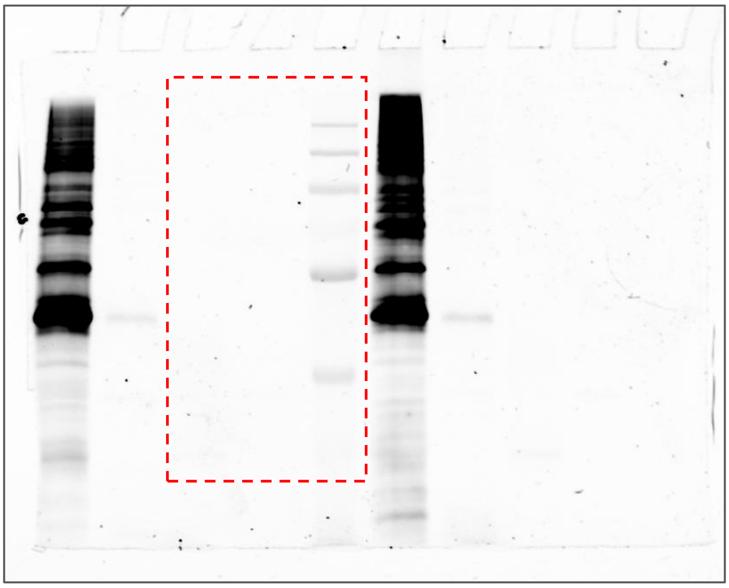

EDF 10c, Coomassie

Used in Extended Data Fig. 10c

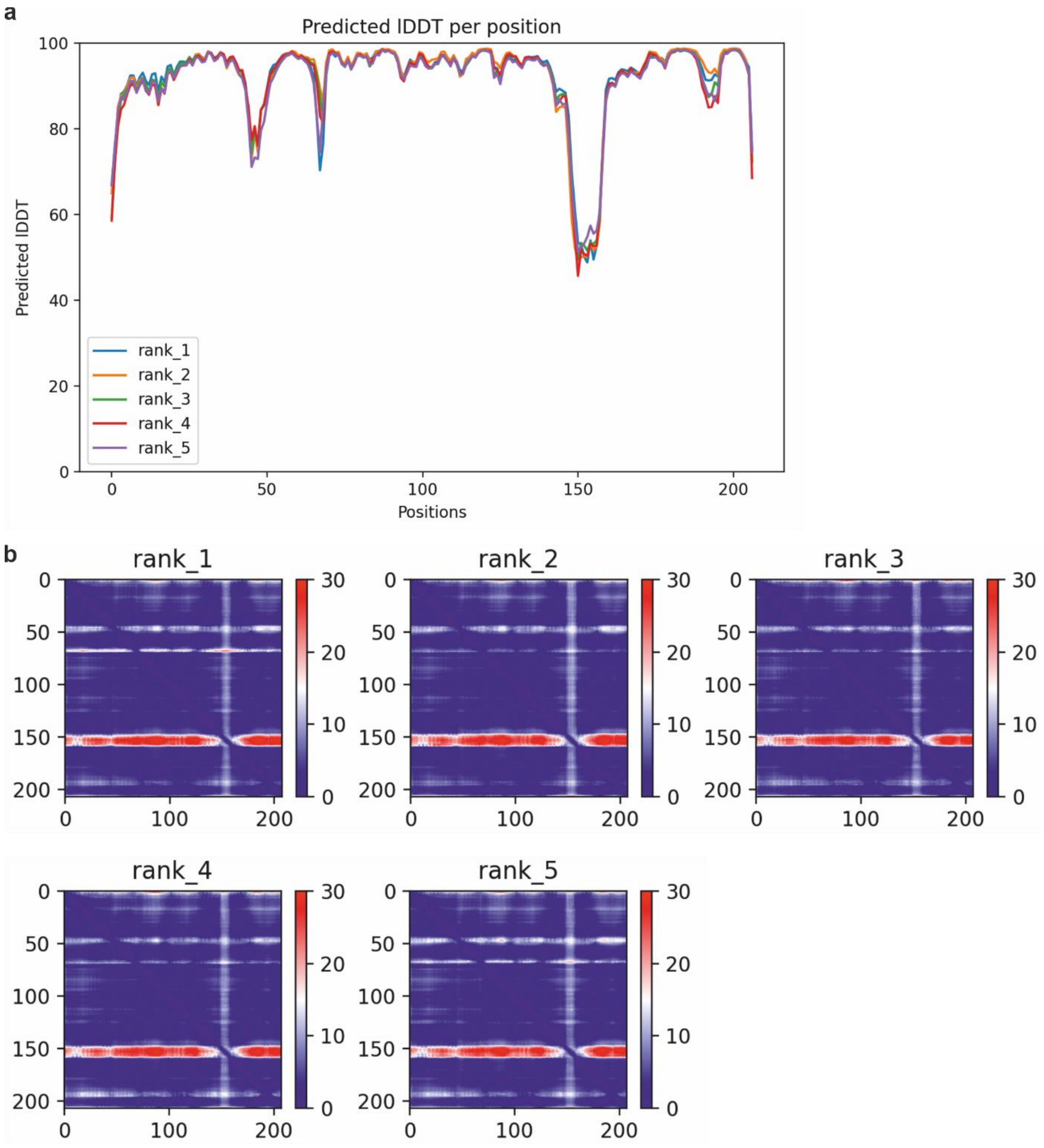

Supplementary Figure 2: Confidence metrics of the Alphafold prediction model of ModB structure shown in Extended Data Fig. 1f. Plots of predicted local-distance difference test (pLDDT) (a) and predicted aligned error (PAE) (b) are shown as Alphafold prediction metrics [63]. Confidence metrics for the model used here are represented by “rank_1”.

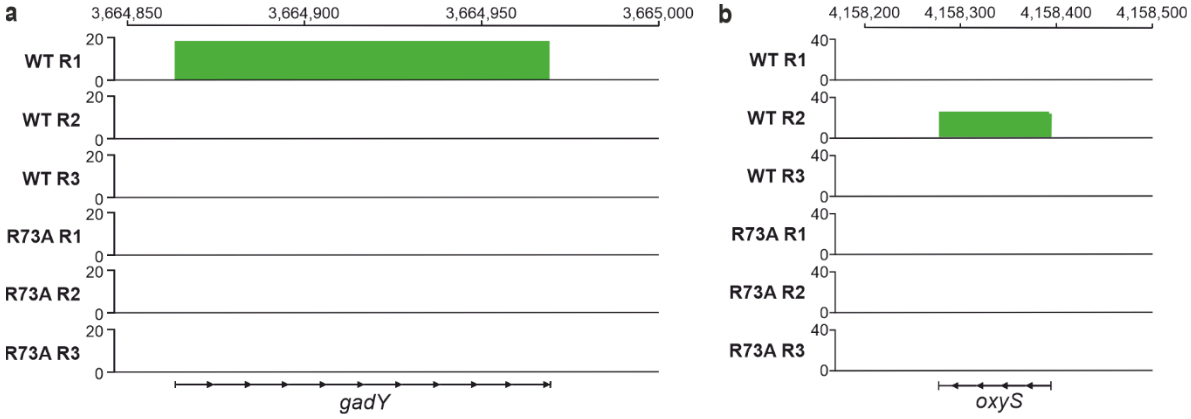

Supplementary Figure 3: IGV coverage plots for RNAylomeSeq data. Read coverage on identified RNAylated RNAs as analysed in IGV is exemplarily shown for *gadY* (**a**) and *oxyS* (**b**) depicting reads in T4 WT samples (green) vs. T4 ModB R73A, G74A samples (red) for each replicate. RNAylomeSeq merely identifies 5'-termini of mRNAs or, if 200nt or smaller, entire sRNA sequences. This is due to the application of single-end Illumina-Seq which automatically only captures the 5'-end of the respective read/transcript.

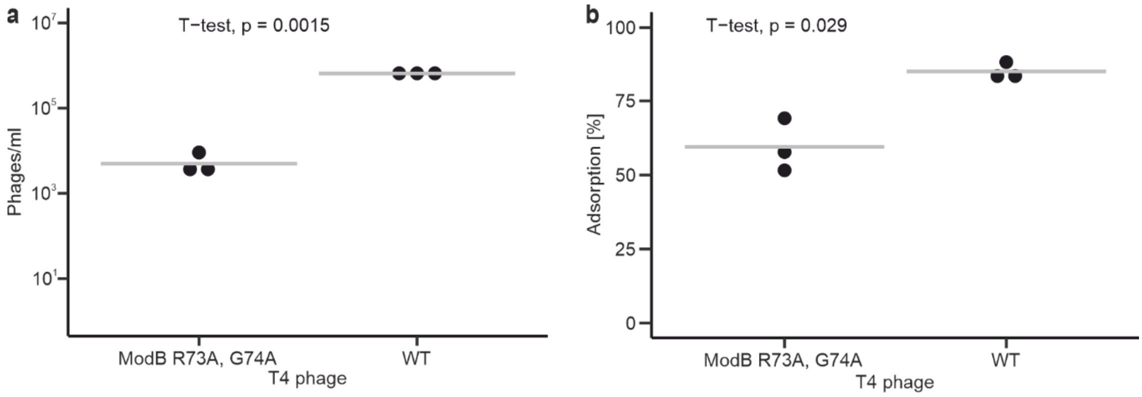

Supplementary Figure 4: Statistical tests for phenotype of T4 phage ModB R73A, G74A mutant shown in Fig. 5 c-e. Dotplots are shown for burst size 140 min post-infection (**a**) and phage adsorption to the host cell 8 min post-infection (**b**). Grey bars represent mean, black dots individual data points of n=3 biologically independent replicates each. Two-sided t-tests found both differences in burst size (140 min post infection; t-test, two-sided, p-value = 0.0015 at $p_{signif.} < 0.05$) and phage adsorption between T4 phage WT and T4 phage R73A, G74A (8 min post infection; t-test, two-sided, p-value = 0.029 at $p_{signif.} < 0.05$) to be statistically significant on a significance level of 0.05. T4 phage ModB R73A, G74A produces less progeny and adsorbs less efficiently to the host cell.

Supplementary Table 1: MaxQuant Output for LC-MS/MS analysis of endogenously His-tagged rS1 from T4 phage-infected *E. coli* B strain. Endogenously His-tagged rS1 was isolated from T4 phage-infected (inf) and -uninfected *E. coli* B strain and subjected to LC-MS/MS analysis in biological triplicates (n=3). Intensities from MaxQuant are only shown for rS1 (1A; modificationSpecificPeptides). ADP-ribosylation is detected only for a small subset of rS1 peptides from T4 phage-infected samples whilst absent in uninfected samples. Predominantly, R139/R142 and R485/R487 were identified as ADP-ribosylation sites in all three replicates. ADP-ribosylated peptides are listed by rS1 domain and with the respective arginine residues in 1B; ADPr peptides. Modifications occur at R485/R487 (domain 6) and R139/R142 (domain 2) in all three replicates. Comparing intensities of ADP-ribosylated and unmodified peptides (1C; ADPr vs. unmodified peptides) shows ratios varying from 1.4 % to 6.6 %. Based on this data, one may speculate that R139/R142 and R485/R487 might be major ADP-ribosylation sites on rS1 *in vivo*. MaxQuant parameters for the presented data are presented in 1D; Parameters MaxQuant.

Supplementary Table 2: MaxQuant Output for LC-MS/MS analysis of His-tagged rS1-WT, -R139A and -R139K mutants from T4 phage-infected *E. coli*. MaxQuant Output filtered for rS1 protein and sorted according to ADP-ribosylation (ADP-ribosylwoDP) is presented (2A; modificationSpecificPeptides). T4 phage-infected samples (T4 phage) and -uninfected control (LB control) per rS1 version (WT, R139A or R139K mutant) are presented. A total of three biological replicates (n=3) were analysed per rS1 version. Peptides which were found ADP-ribosylated are listed and are assigned to their respective location (rS1 domain) and the modified arginines in the rS1 protein (2B; ADPr peptides). Intensities of ADP-ribosylated peptides are compared to their unmodified counterpart each by dividing the respective intensities (2C; ADPr vs. unmodified peptides). The peptide AFLPGSLVDVR(K/A)PVRDTLHLEGK is found ADP-ribosylated for rS1 WT, rS1 R139A and R139K only in T4 phage-infected samples across all three replicates. It becomes obvious that for the WT peptide high intensities of the ADP-ribosylated peptide relative to the unmodified peptide are detected across all three T4 phage-infected replicates. For the mutant peptides R139A and R139K, these intensities are at least 3-fold lower. Based on this finding, one may speculate that R139 mutation might reduce ADP-ribosylation of the AFLPGSLVDVRPVRDTLHLEGK peptide in rS1. MaxQuant parameters for the presented data are presented in 2D; Parameters MaxQuant.

Supplementary Table 3: *In vitro* ADP-ribosylation and RNAylation sites in rS1 protein as identified by LC-MS/MS analysis. Peptide spectrum match (PSM) information for ADP-ribosylated and/or RNAylated rS1 peptides are given in summarised form (pivot) and as complete output from OpenMS tool RNPxl (PSMs). Results were filtered for 1 % FDR on PSM level and q-values (scores) are given. Spectrum IDs, precursor m/z values, charge states, best localisation of modification within the peptide sequence, localisation score and mass errors (in ppm) are provided in "PSMs" sheet.

Supplementary Table 4: Genes identified to contribute to the RNAylome by RNAylomeSeq. An excerpt from the counts table is presented. Hits are calculated based on the mean read counts for each gene among T4 WT and R73A, G74A (MUT) samples for each replicate individually. For a hit, the log2 Fold Change (LFC) between WT and MUT sample is to be greater than 1.5 and the log2 transformed mean expression greater than -0.5. Hits are indicated as "+" for individual replicates. Read distribution for hits is presented in column "IGV" and the existence of corresponding NAD-capped transcripts is indicated in column "NAD-capped RNA?". Raw read counts are shown in WT_R1 – MUT_R3. Some hits are present in all replicates, some in one or two replicates only. Importantly, for the majority of protein_coding and ncRNA genes, reads initiate with an adenosine or contain an adenosine no more than 2 nt away from the read start. tRNA and rRNA (which more likely represent the background) hits are more abundant in replicates 2 and 3. Especially in replicate 3, the fraction of RNAI reads varies comparing WT and MUT samples, which may explain this variation from the background.

Supplementary Table 5: MaxQuant Output for LC-MS/MS analysis of *E. coli* cell lysate with addition of ModB to various concentrations. Samples 1 and 3 represent lysates without ModB, whilst samples 2 and 4 contain 85

nM and 8.5 nM ModB, respectively. The data serve as raw data for values shown in Extended Data Table 3, where log2 transformed protein intensity values are shown for each sample.

Supplementary Table 6: *In vitro* ADP-ribosylation and RNAylation sites in rL2 protein as identified by LC-MS/MS analysis. Peptide spectrum match (PSM) information for ADP-ribosylated and/or RNAylated rL2 peptides are given in summarised form (pivot) and as complete output from OpenMS tool RNPxl (PSMs). Results were filtered for 1 % FDR on PSM level and q-values (scores) are given. Spectrum IDs, precursor m/z values, charge states, best localisation of modification within the peptide sequence, localisation score and mass errors (in ppm) are provided in "PSMs" sheet.

Supplementary Table 7: RNAs used in this study.

RNA	RNA sequence
**8mer**	ACAGUAUU
**RNAI**	ACAGUAUUUGGUAUCUGCGCUCUGCUGAAGCCAGUUACCUUCGGAAAAAGAGUUGGUAGCUCUUGAUCCGGCAAACAAACCACCGCUGGUAGCGGUGGUUUUUUUGUU
**100nt-RNA (Qβ)**	AUCUUGAUACUACCUUUAGUUCGUUUAAACACGUUCUUGAUAGUAUCUUUUUAUUAACCCAACGCGUAAAGCGUUGAAACUUUGGGUCAAUUUGAUCAUG
**10mer-Cy5**	ACAGUAUUUG
**2nt-5'overhang-Cy5**	ACAGACUUCGGUCU-Cy5
**3'overhang-Cy5**	AGACUUCGGUCUA-Cy5
**5'P-blunt-Cy5**	AGACUUCGGUCU-Cy5
**linear-Cy5**	AGACUUCGAC-Cy5
**40mer-Cy5**	ACAGUAUUUGGUAUCUGCGCUCUGCUGAAGCCAGUUACUU-Cy5

Supplementary Table 8: Genomic DNA sequence of ARTs, rS1 variants and ADP-ribose hydrolases. Start codon in italic; thrombin cleavage site in bold; mutations in red and bold; restriction sites underlined.

Gene [5', 3' restriction site]	DNA sequence
Alt [*NcoI*, *XhoI*]	CC*ATG*GGAGAACTTATTACAGAATTATTTGACGAAGATACTACTCTTCCAATTACAAACTTAT<br>ATCCAAAGAAGAAAATACCGCAAATTTTTTCAGTTCATGTTGATGATGCAATTGAACAACCAG<br>GCTTTCGTTTATGTACCTATACATCTGGAGGTGATACTAATCGTGATTTAAAGATGGGCGATA<br>AAATGATGCATATTGTTCCTTTTACATTAACTGCTAAAGGTTCAATTGCTAAATTAAAAGGTCT<br>TGGTCCAAGCCCAATTAATTATATCAATTCAGTTTTTACTGTTGCAATGCAAACAATGCGCCA<br>GTATAAAATTGATGCCTGTATGCTCCGTATTCTTAAGTCTAAAACTGCTGGCCAAGCTCGACA<br>AATTCAAGTTATTGCTGATAGACTTATCCGTAGTCGTTCAGGTGGTAGATACGTCCTTCTTAA<br>GGAACTCTGGGATTACGATAAAAAGTATGCATATATTCTTATACATCGCAAAAATGTATCACT<br>AGAAGACATTCCAGGAGTTCCGGAAATTAGTACCGAGCTCTTTACTAAAGTTGAATCGAAGG<br>TCGGTGATGTTTATATCAATAAAGATACTGGGGCTCAAGTAACTAAAAATGAGGCAATTGCA<br>GCATCTATTGCGCAAGAAAATGATAAACGTTCTGACCAAGCTGTAATCGTTAAAGTTAAAATT<br>TCCCGTAGAGCAATTGCGCAAAGTCAGTCATTGGAATCTTCTAGATTTGAAACACCAATGTTT<br>CAAAAATTTGAGGCTTCAGCGGCCGAATTAAATAAACCAGCGGACGCGCCTTTAATTTCTGAT<br>TCTAATGAATTAACGGTAATTTCTACTTCAGGATTTGCACTAGAGAATGCTCTTAGCAGTGTT<br>ACAGCTGGGATGGCATTCAGAGAAGCTTCTATAATTCCTGAAGATAAAGAATCCATTATTAAC<br>GCAGAAATAAAAAATAAAGCTTTAGAAAGATTACGAAAAGAATCTATTACTTCAATAAAAAC<br>CTTAGAAACTATTGCTTCTATCGTCGATGATACTTTAGAAAAATATAAGGGTGCTTGGTTTGA<br>AAGAAATATTAACAAACATTCGCATTTAAACCAAGATGCTGCAAATGAGTTAGTACAAAATTC<br>TTGGAATGCAATAAAAACAAAGATTATTCGAAGAGAATTACGTGGATATGCTCTTACCGCTG<br>GATGGTCATTACATCCTATAGTCGAAAATAAAGATTCATCTAAATACACACCAGCGCAAAAAC<br>GCGGAATTCGTGAATACGTAGGTTCAGGATATGTAGACATAAATAATGCTCTTTTGGGATTAT<br>ATAATCCAGATGAGCGTACAAGTATTTTGACAGCATCTGACATAGAAAAAGCTATTGATAATT<br>TAGATTCAGCCTTTAAAAATGGTGAACGATTACCAAAAGGTATTACTTTGTATCGTTCACAAC<br>GAATGTTACCTTCAATATACGAAGCAATGGTAAAAAATCGAGTTTTTTATTTTAGAAACTTTGT<br>GTCAACATCATTATATCCAAATATTTTTGGTACTTGGATGACTGATTCATCTATAGGTGTTTTA<br>CCAGACGAAAAGCGTTTAAGCGTTTCTATTGATAAAACTGATGAAGGACTTGTAAATTCTAGC<br>GATAATTTAGTTGGAATTGGATGGGTTATTACTGGGGCTGATAAGGTCAATGTTGTTTTACCC<br>GGTGGAAGTTTAGCGCCTTCAAATGAAATGGAAGTCATTTTGCCACGTGGATTAATGGTCAA<br>AGTTAATAAAATAACCGATGCATCTTACAATGATGGAACAGTTAAAACTAACAACAAGCTTAT<br>TCAAGCTGAAGTTATGACCACAGAAGAACTCACCGAATCGGTAATCTATGACGGAGACCATT<br>TAATGGAAACTGGTGAATTGGTTACAATGACAGGTGATATAGAAGATAGAGTTGACTTTGCA<br>TCATTTGTTTCATCAAATGTTAAACAGAAAGTAGAATCATCTCTTGGAATTATTGCGTCTTGCA<br>TAGATATTGCAAACATGCCTTACAAGTTCGTTCAAGGA**CTGGTGCCGCGCGGCAGC**CTCGAG
ModA [*NcoI*, *XhoI*]	CC*ATG*GGAAAATACTCAGTAATGCAACTAAAAGATTTTAAAATAAAATCAATGGATGCATCG<br>GTGCGTGCTTCTATTCGTGAAGAATTACTTTCTGAAGGGTTTAATTTATCTGAAATTGAACTTT<br>TAATTCATTGTATTACTAATAAACCAGATGACCATTCTTGGTTAAATGAAATAATCAAATCTCG<br>TTTGGTTCCAAACGATAAACCTCTTTGGAGAGGTGTTCCAGCTGAGACTAAACAAGTATTAAA<br>TCAAGGAATTGATATTATTACATTTGATAAAGTCGTATCAGCTTCATATGATAAAAATATAGC

	TCTACATTTTGCTTCTGGTTTAGAGTATAACACACAAGTTATTTTTGAATTCAAAGCTCCTATG<br>GTATTCAATTTCCAGGAGTATGCTATAAAAGCTCTACGCTGTAAAGAATACAATCCAAACTTT<br>AAGTTTCCGGATAGTCATCGTTATCGTAATATGGAATTAGTTTCAGATGAACAAGAAGTAATG<br>ATACCAGCTGGAAGTGTATTTAGAATTGCAGATAGATATGAGTATAAAAAGTGTTCAACATA<br>CACTATCTATACTCTTGATTTTGAAGGATTTAATCTA**CTGGTGCCGCGCGGCAGC**CTCGAG
**ModB [*NcoI*, *XhoI*]**	CC*ATG*GGAATTATTAATCTTGCAGATGTTGAACAGTTATCTATAAAAGCTGAAAGCGTTGATT<br>TTCAATATGATATGTATAAAAAGGTCTGTGAAAAATTTACTGACTTTGAGCAGTCTGTTCTTTG<br>GCAATGTATGGAAGCCAAAAAGAATGAAGCTCTTCATAAGCATTTAAATGAAATCATTAAAA<br>AGCATTTAACTAAATCGCCTTATCAATTATATCGTGGTATATCAAAATCGACAAAAGAACTCA<br>TTAAAGATTTACAAGTTGGAGAAGTGTTTTCAACGAACAGGGTAGATTCATTTACTACTAGTT<br>TGCATACAGCGTGTTCTTTTTCTTATGCTGAATATTTCACTGAAACAATACTTCGTTTAAAAAC<br>TGATAAAGCTTTTAATTATTCTGACCATATCAGCGATATTATACTTTCTTCTCCTAATACTGAGT<br>TTAAGTACACGTATGAAGATACTGATGGATTAGATTCAGAGCGTACTGATAACTTAATGATG<br>ATTGTGCGTGAACAAGAATGGATGATTCCAATTGGAAAGTATAAAATAACTTCTATTTCAAAA<br>GAAAAATTACACGATTCATTTGGAACATTTAAAGTTTATGATATTGAGGTAGTTGAA**CTGGTG**<br>**CCGCGCGGCAGC**CTCGAG
**ModB R73A, G74A [*NcoI*/*XhoI*]**	*CCATGG*GAATTATTAATCTTGCAGATGTTGAACAGTTATCTATAAAAGCTGAAAGCGTTGATT<br>TTCAATATGATATGTATAAAAAGGTCTGTGAAAAATTTACTGACTTTGAGCAGTCTGTTCTTTG<br>GCAATGTATGGAAGCCAAAAAGAATGAAGCTCTTCATAAGCATTTAAATGAAATCATTAAAA<br>AGCATTTAACTAAATCGCCTTATCAATTATAT**GCGGCA**ATATCAAAATCGACAAAAGAACTCA<br>TTAAAGATTTACAAGTTGGAGAAGTGTTTTCAACGAACAGGGTAGATTCATTTACTACTAGTT<br>TGCATACAGCGTGTTCTTTTTCTTATGCTGAATATTTCACTGAAACAATACTTCGTTTAAAAAC<br>TGATAAAGCTTTTAATTATTCTGACCATATCAGCGATATTATACTTTCTTCTCCTAATACTGAGT<br>TTAAGTACACGTATGAAGATACTGATGGATTAGATTCAGAGCGTACTGATAACTTAATGATG<br>ATTGTGCGTGAACAAGAATGGATGATTCCAATTGGAAAGTATAAAATAACTTCTATTTCAAAA<br>GAAAAATTACACGATTCATTTGGAACATTTAAAGTTTATGATATTGAGGTAGTTGAA**CTGGTG**<br>**CCGCGCGGCAGC**CTCGAGCACCACCACCACCACCACTGA
**pET28-rS1 [*NcoI*, *XhoI*]**	CC*ATG*GGAACTGAATCTTTGCGGCATGCTCAACTCTTTGAAGAGTCCTTAAAAGAAATCGAA<br>ACCCGCCCGGGTTCTATCGTTCGTGGCGTTGTTGTTGCTATCGACAAAGACGTAGTACTGGTT<br>GACGCTGGTCTGAAATCTGAGTCCGCCATCCCGGCTGAGCAGTTCAAAAACGCCCAGGGCGA<br>GCTGGAAATCCAGGTAGGTGACGAAGTTGACGTTGCTCTGGACGCAGTAGAAGACGGCTTC<br>GGTGAAACTCTGCTGTCCCGTGAGAAAGCTAAACGTCACGAAGCCTGGATCACGCTGGAAAA<br>AGCTTACGAAGATGCTGAAACTGTTACCGGTGTTATCAACGGCAAAGTTAAGGGCGGCTTCA<br>CTGTTGAGCTGAACGGTATTCGTGCGTTCCTGCCAGGTTCTCTGGTAGACGTTCGTCCGGTGC<br>GTGACACTCTGCACCTGGAAGGCAAAGAGCTTGAATTTAAAGTAATCAAGCTGGATCAGAAG<br>CGCAACAACGTTGTTGTTTCTCGTCGTGCCGTTATCGAATCCGAAAACAGCGCAGAGCGCGA<br>TCAGCTGCTGGAAAACCTGCAGGAAGGCATGGAAGTTAAAGGTATCGTTAAGAACCTCACTG<br>ACTACGGTGCATTCGTTGATCTGGGCGGCGTTGACGGCCTGCTGCACATCACTGACATGGCC<br>TGGAAACGCGTTAAGCATCCGAGCGAAATCGTCAACGTGGGCGACGAAATCACTGTTAAAGT<br>GCTGAAGTTCGACCGCGAACGTACCCGTGTATCCCTGGGCCTGAAACAGCTGGGCGAAGATC<br>CGTGGGTAGCTATCGCTAAACGTTATCCGGAAGGTACCAAACTGACTGGTCGCGTGACCAAC<br>CTGACCGACTACGGCTGCTTCGTTGAAATCGAAGAAGGCGTTGAAGGCCTGGTACACGTTTC<br>CGAAATGGACTGGACCAACAAAAACATCCACCCGTCCAAAGTTGTTAACGTTGGCGATGTAG<br>TGGAAGTTATGGTTCTGGATATCGACGAAGAACGTCGTCGTATCTCCCTGGGTCTGAAACAG

	TGCAAAGCTAACCCGTGGCAGCAGTTCGCGGAAACCCACAACAAGGGCGACCGTGTTGAAG<br>GTAAAATCAAGTCTATCACTGACTTCGGTATCTTCATCGGCTTGGACGGCGGCATCGACGGCC<br>TGGTTCACCTGTCTGACATCTCCTGGAACGTTGCAGGCGAAGAAGCAGTTCGTGAATACAAA<br>AAAGGCGACGAAATCGCTGCAGTTGTTCTGCAGGTTGACGCAGAACGTGAACGTATCTCCCT<br>GGGCGTTAAACAGCTCGCAGAAGATCCGTTCAACAACTGGGTTGCTCTGAACAAGAAAGGC<br>GCTATCGTAACCGGTAAAGTAACTGCAGTTGACGCTAAAGGCGCAACCGTAGAACTGGCTGA<br>CGGCGTTGAAGGTTACCTGCGTGCTTCTGAAGCATCCCGTGACCGCGTTGAAGACGCTACCC<br>TGGTTCTGAGCGTTGGCGACGAAGTTGAAGCTAAATTCACCGGCGTTGATCGTAAAAACCGC<br>GCAATCAGCCTGTCTGTTCGTGCGAAAGACGAAGCTGACGAGAAAGATGCAATCGCAACTGT<br>TAACAAACAGGAAGATGCAAACTTCTCCAACAACGCAATGGCTGAAGCTTTCAAAGCAGCTA<br>AAGGCGAG**CTGGTGCCGCGCGGCAGC**CTCGAG
**pET28-rS1 R139A [*NcoI*, *XhoI*]**	CCATGGGAACTGAATCTTTGCGGCATGCTCAACTCTTTGAAGAGTCCTTAAAAGAAATCGAA<br>ACCCGCCCGGGTTCTATCGTTCGTGGCGTTGTTGTTGCTATCGACAAAGACGTAGTACTGGTT<br>GACGCTGGTCTGAAATCTGAGTCCGCCATCCCGGCTGAGCAGTTCAAAAACGCCCAGGGCGA<br>GCTGGAAATCCAGGTAGGTGACGAAGTTGACGTTGCTCTGGACGCAGTAGAAGACGGCTTC<br>GGTGAAACTCTGCTGTCCCGTGAGAAAGCTAAACGTCACGAAGCCTGGATCACGCTGGAAAA<br>AGCTTACGAAGATGCTGAAACTGTTACCGGTGTTATCAACGGCAAAGTTAAGGGCGGCTTCA<br>CTGTTGAGCTGAACGGTATTCGTGCGTTCCTGCCAGGTTCTCTGGTAGACGTT**GCC**CCGGTGC<br>GTGACACTCTGCACCTGGAAGGCAAAGAGCTTGAATTTAAAGTAATCAAGCTGGATCAGAAG<br>CGCAACAACGTTGTTGTTTCTCGTCGTGCCGTTATCGAATCCGAAAACAGCGCAGAGCGCGA<br>TCAGCTGCTGGAAAACCTGCAGGAAGGCATGGAAGTTAAAGGTATCGTTAAGAACCTCACTG<br>ACTACGGTGCATTCGTTGATCTGGGCGGCGTTGACGGCCTGCTGCACATCACTGACATGGCC<br>TGGAAACGCGTTAAGCATCCGAGCGAAATCGTCAACGTGGGCGACGAAATCACTGTTAAAGT<br>GCTGAAGTTCGACCGCGAACGTACCCGTGTATCCCTGGGCCTGAAACAGCTGGGCGAAGATC<br>CGTGGGTAGCTATCGCTAAACGTTATCCGGAAGGTACCAAACTGACTGGTCGCGTGACCAAC<br>CTGACCGACTACGGCTGCTTCGTTGAAATCGAAGAAGGCGTTGAAGGCCTGGTACACGTTTC<br>CGAAATGGACTGGACCAACAAAAACATCCACCCGTCCAAAGTTGTTAACGTTGGCGATGTAG<br>TGGAAGTTATGGTTCTGGATATCGACGAAGAACGTCGTCGTATCTCCCTGGGTCTGAAACAG<br>TGCAAAGCTAACCCGTGGCAGCAGTTCGCGGAAACCCACAACAAGGGCGACCGTGTTGAAG<br>GTAAAATCAAGTCTATCACTGACTTCGGTATCTTCATCGGCTTGGACGGCGGCATCGACGGCC<br>TGGTTCACCTGTCTGACATCTCCTGGAACGTTGCAGGCGAAGAAGCAGTTCGTGAATACAAA<br>AAAGGCGACGAAATCGCTGCAGTTGTTCTGCAGGTTGACGCAGAACGTGAACGTATCTCCCT<br>GGGCGTTAAACAGCTCGCAGAAGATCCGTTCAACAACTGGGTTGCTCTGAACAAGAAAGGC<br>GCTATCGTAACCGGTAAAGTAACTGCAGTTGACGCTAAAGGCGCAACCGTAGAACTGGCTGA<br>CGGCGTTGAAGGTTACCTGCGTGCTTCTGAAGCATCCCGTGACCGCGTTGAAGACGCTACCC<br>TGGTTCTGAGCGTTGGCGACGAAGTTGAAGCTAAATTCACCGGCGTTGATCGTAAAAACCGC<br>GCAATCAGCCTGTCTGTTCGTGCGAAAGACGAAGCTGACGAGAAAGATGCAATCGCAACTGT<br>TAACAAACAGGAAGATGCAAACTTCTCCAACAACGCAATGGCTGAAGCTTTCAAAGCAGCTA<br>AAGGCGAG**CTGGTGCCGCGCGGCAGC**CTCGAG
**pET28-rS1 R139K [*NcoI*, *XhoI*]**	CCATGGGAACTGAATCTTTGCGGCATGCTCAACTCTTTGAAGAGTCCTTAAAAGAAATCGAA<br>ACCCGCCCGGGTTCTATCGTTCGTGGCGTTGTTGTTGCTATCGACAAAGACGTAGTACTGGTT<br>GACGCTGGTCTGAAATCTGAGTCCGCCATCCCGGCTGAGCAGTTCAAAAACGCCCAGGGCGA<br>GCTGGAAATCCAGGTAGGTGACGAAGTTGACGTTGCTCTGGACGCAGTAGAAGACGGCTTC<br>GGTGAAACTCTGCTGTCCCGTGAGAAAGCTAAACGTCACGAAGCCTGGATCACGCTGGAAAA

	AGCTTACGAAGATGCTGAAACTGTTACCGGTGTTATCAACGGCAAAGTTAAGGGCGGCTTCA<br>CTGTTGAGCTGAACGGTATTCGTGCGTTCCTGCCAGGTTCTCTGGTAGACGTT**AAA**CCGGTG<br>CGTGACACTCTGCACCTGGAAGGCAAAGAGCTTGAATTTAAAGTAATCAAGCTGGATCAGAA<br>GCGCAACAACGTTGTTGTTTCTCGTCGTGCCGTTATCGAATCCGAAAACAGCGCAGAGCGCG<br>ATCAGCTGCTGGAAAACCTGCAGGAAGGCATGGAAGTTAAAGGTATCGTTAAGAACCTCACT<br>GACTACGGTGCATTCGTTGATCTGGGCGGCGTTGACGGCCTGCTGCACATCACTGACATGGC<br>CTGGAAACGCGTTAAGCATCCGAGCGAAATCGTCAACGTGGGCGACGAAATCACTGTTAAA<br>GTGCTGAAGTTCGACCGCGAACGTACCCGTGTATCCCTGGGCCTGAAACAGCTGGGCGAAG<br>ATCCGTGGGTAGCTATCGCTAAACGTTATCCGGAAGGTACCAAACTGACTGGTCGCGTGACC<br>AACCTGACCGACTACGGCTGCTTCGTTGAAATCGAAGAAGGCGTTGAAGGCCTGGTACACGT<br>TTCCGAAATGGACTGGACCAACAAAAACATCCACCCGTCCAAAGTTGTTAACGTTGGCGATG<br>TAGTGGAAGTTATGGTTCTGGATATCGACGAAGAACGTCGTCGTATCTCCCTGGGTCTGAAA<br>CAGTGCAAAGCTAACCCGTGGCAGCAGTTCGCGGAAACCCACAACAAGGGCGACCGTGTTG<br>AAGGTAAAATCAAGTCTATCACTGACTTCGGTATCTTCATCGGCTTGGACGGCGGCATCGAC<br>GGCCTGGTTCACCTGTCTGACATCTCCTGGAACGTTGCAGGCGAAGAAGCAGTTCGTGAATA<br>CAAAAAAGGCGACGAAATCGCTGCAGTTGTTCTGCAGGTTGACGCAGAACGTGAACGTATCT<br>CCCTGGGCGTTAAACAGCTCGCAGAAGATCCGTTCAACAACTGGGTTGCTCTGAACAAGAAA<br>GGCGCTATCGTAACCGGTAAAGTAACTGCAGTTGACGCTAAAGGCGCAACCGTAGAACTGG<br>CTGACGGCGTTGAAGGTTACCTGCGTGCTTCTGAAGCATCCCGTGACCGCGTTGAAGACGCT<br>ACCCTGGTTCTGAGCGTTGGCGACGAAGTTGAAGCTAAATTCACCGGCGTTGATCGTAAAAA<br>CCGCGCAATCAGCCTGTCTGTTCGTGCGAAAGACGAAGCTGACGAGAAAGATGCAATCGCA<br>ACTGTTAACAAACAGGAAGATGCAAACTTCTCCAACAACGCAATGGCTGAAGCTTTCAAAGC<br>AGCTAAAGGCGAG**CTGGTGCCGCGCGGCAGC**CTCGAG
**pTAC-rS1**<br>**[*XhoI*, *SphI*]**	*ATG*AAGCTTCCTCGAGAGACTGAATCTTTTGCTCAACTCTTTGAAGAGTCCTTAAAAGAAATCGAAACC<br>CGCCCGGGTTCTATCGTTCGTGGCGTTGTTGTTGCTATCGACAAAGACGTAGTACTGGTTGACGCTGGT<br>CTGAAATCTGAGTCCGCCATCCCGGCTGAGCAGTTCAAAAACGCCCAGGGCGAGCTGGAAATCCAGGT<br>AGGTGACGAAGTTGACGTTGCTCTGGACGCAGTAGAAGACGGCTTCGGTGAAACTCTGCTGTCCCGTG<br>AGAAAGCTAAACGTCACGAAGCCTGGATCACGCTGGAAAAAGCTTACGAAGATGCTGAAACTGTTACC<br>GGTGTTATCAACGGCAAAGTTAAGGGCGGCTTCACTGTTGAGCTGAACGGTATTCGTGCGTTCCTGCC<br>AGGTTCTCTGGTAGACGTTCGTCCGGTGCGTGACACTCTGCACCTGGAAGGCAAAGAGCTTGAATTTA<br>AAGTAATCAAGCTGGATCAGAAGCGCAACAACGTTGTTGTTTCTCGTCGTGCCGTTATCGAATCCGAAA<br>ACAGCGCAGAGCGCGATCAGCTGCTGGAAAACCTGCAGGAAGGCATGGAAGTTAAAGGTATCGTTAA<br>GAACCTCACTGACTACGGTGCATTCGTTGATCTGGGCGGCGTTGACGGCCTGCTGCACATCACTGACAT<br>GGCCTGGAAACGCGTTAAGCATCCGAGCGAAATCGTCAACGTGGGCGACGAAATCACTGTTAAAGTGC<br>TGAAGTTCGACCGCGAACGTACCCGTGTATCCCTGGGCCTGAAACAGCTGGGCGAAGATCCGTGGGTA<br>GCTATCGCTAAACGTTATCCGGAAGGTACCAAACTGACTGGTCGCGTGACCAACCTGACCGACTACGG<br>CTGCTTCGTTGAAATCGAAGAAGGCGTTGAAGGCCTGGTACACGTTTCCGAAATGGACTGGACCAACA<br>AAAACATCCACCCGTCCAAAGTTGTTAACGTTGGCGATGTAGTGGAAGTTATGGTTCTGGATATCGACG<br>AAGAACGTCGTCGTATCTCCCTGGGTCTGAAACAGTGCAAAGCTAACCCGTGGCAGCAGTTCGCGGAA<br>ACCCACAACAAGGGCGACCGTGTTGAAGGTAAAATCAAGTCTATCACTGACTTCGGTATCTTCATCGGC<br>TTGGACGGCGGCATCGACGGCCTGGTTCACCTGTCTGACATCTCCTGGAACGTTGCAGGCGAAGAAGC<br>AGTTCGTGAATACAAAAAAGGCGACGAAATCGCTGCAGTTGTTCTGCAGGTTGACGCAGAACGTGAAC<br>GTATCTCCCTGGGCGTTAAACAGCTCGCAGAAGATCCGTTCAACAACTGGGTTGCTCTGAACAAGAAA<br>GGCGCTATCGTAACCGGTAAAGTAACTGCAGTTGACGCTAAAGGCGCAACCGTAGAACTGGCTGACG<br>GCGTTGAAGGTTACCTGCGTGCTTCTGAAGCATCCCGTGACCGCGTTGAAGACGCTACCCTGGTTCTGA<br>GCGTTGGCGACGAAGTTGAAGCTAAATTCACCGGCGTTGATCGTAAAAACCGCGCAATCAGCCTGTCT

	GTTCGTGCGAAAGACGAAGCTGACGAGAAAGATGCAATCGCAACTGTTAACAAACAGGAAGATGCAA<br>ACTTCTCCAACAACGCAATGGCTGAAGCTTTCAAAGCAGCTAAAGGCGAGTGCATGCACGTAGAG
**S1 D1 [*NcoI*, *XhoI*]**	C*CATG*GAGTCCTTAAAAGAAATCGAAACCCGCCCGGGTTCTATCGTTCGTGGCGTTGTTGTTG<br>CTATCGACAAAGACGTAGTACTGGTTGACGCTGGTCTGAAATCTGAGTCCGCCATCCCGGCT<br>GAGCAGTTCAAAAACGCCCAGGGCGAGCTGGAAATCCAGGTAGGTGACGAAGTTGACGTTG<br>CTCTGGACGCAGTAGAAGACGGCTTCGGTGAAACTCTGCTGTCCCGTGAGAAAGCTAAACGT<br>CACGAAGCC**CTGGTGCCGCGCGGCAGC**CTCGAG
**S1 D2 [*NcoI*, *XhoI*]**	C*CATG*GCCTGGATCACGCTGGAAAAAGCTTACGAAGATGCTGAAACTGTTACCGGTGTTATC<br>AACGGCAAAGTTAAGGGCGGCTTCACTGTTGAGCTGAACGGTATTCGTGCGTTCCTGCCAGG<br>TTCTCTGGTAGACGTTCGTCCGGTGCGTGACACTCTGCACCTGGAAGGCAAAGAGCTTGAAT<br>TTAAAGTAATCAAGCTGGATCAGAAGCGCAACAACGTTGTTGTTTCTCGTCGTGCCGTTATCG<br>AATCCGAAAACAGCGCAGAG**CTGGTGCCGCGCGGCAGC**CTCGAG
**S1 D2 R139A [*NcoI*, *XhoI*]**	C*CATG*GCCTGGATCACGCTGGAAAAAGCTTACGAAGATGCTGAAACTGTTACCGGTGTTATC<br>AACGGCAAAGTTAAGGGCGGCTTCACTGTTGAGCTGAACGGTATTCGTGCGTTCCTGCCAGG<br>TTCTCTGGTAGACGTT**GCC**CCGGTGCGTGACACTCTGCACCTGGAAGGCAAAGAGCTTGAAT<br>TTAAAGTAATCAAGCTGGATCAGAAGCGCAACAACGTTGTTGTTTCTCGTCGTGCCGTTATCG<br>AATCCGAAAACAGCGCAGAG**CTGGTGCCGCGCGGCAGC**CTCGAG
**S1 D2 R139K [*NcoI*, *XhoI*]**	C*CATG*GCCTGGATCACGCTGGAAAAAGCTTACGAAGATGCTGAAACTGTTACCGGTGTTATC<br>AACGGCAAAGTTAAGGGCGGCTTCACTGTTGAGCTGAACGGTATTCGTGCGTTCCTGCCAGG<br>TTCTCTGGTAGACGTT**AAA**CCGGTGCGTGACACTCTGCACCTGGAAGGCAAAGAGCTTGAAT<br>TTAAAGTAATCAAGCTGGATCAGAAGCGCAACAACGTTGTTGTTTCTCGTCGTGCCGTTATCG<br>AATCCGAAAACAGCGCAGAG**CTGGTGCCGCGCGGCAGC**CTCGAG
**S1 D3 [*NcoI*, *XhoI*]**	C*CATG*GCCCGCGATCAGCTGCTGGAAAACCTGCAGGAAGGCATGGAAGTTAAAGGTATCGT<br>TAAGAACCTCACTGACTACGGTGCATTCGTTGATCTGGGCGGCGTTGACGGCCTGCTGCACA<br>TCACTGACATGGCCTGGAAACGCGTTAAGCATCCGAGCGAAATCGTCAACGTGGGCGACGA<br>AATCACTGTTAAAGTGCTGAAGTTCGACCGCGAACGTACCCGTGTATCCCTGGGCCTGAAAC<br>AGCTGGGCGAAGATCCG**CTGGTGCCGCGCGGCAGC**CTCGAG
**S1 D4 [*NcoI*, *XhoI*]**	C*CATG*GCCTGGGTAGCTATCGCTAAACGTTATCCGGAAGGTACCAAACTGACTGGTCGCGTG<br>ACCAACCTGACCGACTACGGCTGCTTCGTTGAAATCGAAGAAGGCGTTGAAGGCCTGGTACA<br>CGTTTCCGAAATGGACTGGACCAACAAAAACATCCACCCGTCCAAAGTTGTTAACGTTGGCG<br>ATGTAGTGGAAGTTATGGTTCTGGATATCGACGAAGAACGTCGTCGTATCTCCCTGGGTCTG<br>AAACAGTGCAAAGCTAACCCG**CTGGTGCCGCGCGGCAGC**CTCGAG
**S1 D5 [*NcoI*, *XhoI*]**	C*CATG*GCCTGGCAGCAGTTCGCGGAAACCCACAACAAGGGCGACCGTGTTGAAGGTAAAAT<br>CAAGTCTATCACTGACTTCGGTATCTTCATCGGCTTGGACGGCGGCATCGACGGCCTGGTTCA<br>CCTGTCTGACATCTCCTGGAACGTTGCAGGCGAAGAAGCAGTTCGTGAATACAAAAAAGGCG<br>ACGAAATCGCTGCAGTTGTTCTGCAGGTTGACGCAGAACGTGAACGTATCTCCCTGGGCGTT<br>AAACAGCTCGCAGAAGATCCG**CTGGTGCCGCGCGGCAGC**CTCGAG
**S1 D6 [*NcoI*, *XhoI*]**	C*CATG*GCCTTCAACAACTGGGTTGCTCTGAACAAGAAAGGCGCTATCGTAACCGGTAAAGTA<br>ACTGCAGTTGACGCTAAAGGCGCAACCGTAGAACTGGCTGACGGCGTTGAAGGTTACCTGC<br>GTGCTTCTGAAGCATCCCGTGACCGCGTTGAAGACGCTACCCTGGTTCTGAGCGTTGGCGAC

	GAAGTTGAAGCTAAATTCACCGGCGTTGATCGTAAAAACCGCGCAATCAGCCTGTCTGTTCG TGCGAAAGACGAAGCTGACGAGAAA**CTGGTGCCGCGCGGCAGC**CTCGAG
**S1 domain of PNPase [*NcoI*, *XhoI*]**	C*CATGG*CAGAAATCGAAGTGGGCCGCGTCTACACTGGTAAAGTGACCCGTATCGTTGACTTT GGCGCATTTGTTGCCATCGGCGGCGGTAAAGAAGGTCTGGTCCACATCTCTCAAATCGCTGA CAAACGCGTTGAGAAAGTGACCGATTACCTGCAGATGGGTCAGGAAGTACCGGTGAAAGTT CTGGAAGTTGATCGCCAGGGCCGTATCCGTCTGAGCATTAAAGAAGCGACTGAGCAGTCTCA ACCTGCTGCA**CTGGTGCCGCGCGGCAGC**CTCGAG
**pET28 ARH1 [*NcoI*, *XhoI*]**	C*CATGG*AAAAATACGTCGCCGCGATGGTTTTGTCAGCTGCTGGCGATGCTTTGGGATATTAT AATGGAAAGTGGGAATTTCTTCAGGACGGGGAGAAAATTCATCGTCAACTGGCTCAATTAGG GGGGCTGGATGCTCTGGACGTTGGCCGTTGGCGTGTGTCTGATGATACTGTCATGCACTTGG CAACAGCCGAGGCTTTGGTCGAGGCCGGAAAGGCTCCAAAACTGACTCAGCTTTATTATTTG TTAGCCAAGCACTATCAGGATTGCATGGAAGATATGGACGGTCGCGCACCCGGGGGTGCGT CTGTACACAACGCGATGCAGCTTAAACCTGGGAAACCGAATGGCTGGCGTATCCCATTTAAC TCGCATGAAGGAGGGTGTGGCGCGGCGATGCGCGCGATGTGTATCGGTTTGCGTTTTCCGC ATCACTCTCAATTAGACACACTGATCCAAGTATCGATCGAGTCAGGACGTATGACCCATCATC ACCCGACAGGGTACCTTGGCGCACTTGCGTCCGCCTTATTCACGGCCTATGCGGTAAATAGCC GCCCTCCATTGCAGTGGGGTAAGGGACTTATGGAGCTTTTGCCAGAGGCTAAAAAATACATT GTCCAATCCGGGTACTTTGTGGAAGAAAATTTACAGCATTGGTCTTATTTTCAAACGAAGTGG GAAAACTATCTTAAACTGCGTGGAATCTTGGACGGCGAGAGTGCTCCAACATTCCCTGAATCT TTTGGCGTTAAAGAGCGCGACCAGTTCTACACTTCGTTGTCATATAGTGGCTGGGGCGGTTC ATCTGGGCATGATGCCCCCATGATCGCGTATGACGCGGTGCTGGCGGCGGGAGACTCCTGG AAAGAGCTTGCGCACCGCGCCTTCTTTCACGGAGGTGACTCGGATTCGACCGCAGCCATTGC TGGATGTTGGTGGGGCGTCATGTACGGATTTAAGGGCGTCAGCCCCAGCAACTACGAAAAA TTAGAGTATCGCAATCGCCTTGAGGAAACAGCTCGCGCACTTTACTCGCTGGGTAGTAAAGA AGACACTGTTATCTCGCTG**CTGGTGCCGCGCGGCAGC**CTCGAG
**pET ARH1 D55A, D56A [*NcoI*, *XhoI*]**	C*CATGG*AAAAATACGTCGCCGCGATGGTTTTGTCAGCTGCTGGCGATGCTTTGGGATATTAT AATGGAAAGTGGGAATTTCTTCAGGACGGGGAGAAAATTCATCGTCAACTGGCTCAATTAGG GGGGCTGGATGCTCTGGACGTTGGCCGTTGGCGTGTGTCT**GCGGCG**ACTGTCATGCACTTGG CAACAGCCGAGGCTTTGGTCGAGGCCGGAAAGGCTCCAAAACTGACTCAGCTTTATTATTTG TTAGCCAAGCACTATCAGGATTGCATGGAAGATATGGACGGTCGCGCACCCGGGGGTGCGT CTGTACACAACGCGATGCAGCTTAAACCTGGGAAACCGAATGGCTGGCGTATCCCATTTAAC TCGCATGAAGGAGGGTGTGGCGCGGCGATGCGCGCGATGTGTATCGGTTTGCGTTTTCCGC ATCACTCTCAATTAGACACACTGATCCAAGTATCGATCGAGTCAGGACGTATGACCCATCATC ACCCGACAGGGTACCTTGGCGCACTTGCGTCCGCCTTATTCACGGCCTATGCGGTAAATAGCC GCCCTCCATTGCAGTGGGGTAAGGGACTTATGGAGCTTTTGCCAGAGGCTAAAAAATACATT GTCCAATCCGGGTACTTTGTGGAAGAAAATTTACAGCATTGGTCTTATTTTCAAACGAAGTGG GAAAACTATCTTAAACTGCGTGGAATCTTGGACGGCGAGAGTGCTCCAACATTCCCTGAATCT TTTGGCGTTAAAGAGCGCGACCAGTTCTACACTTCGTTGTCATATAGTGGCTGGGGCGGTTC ATCTGGGCATGATGCCCCCATGATCGCGTATGACGCGGTGCTGGCGGCGGGAGACTCCTGG AAAGAGCTTGCGCACCGCGCCTTCTTTCACGGAGGTGACTCGGATTCGACCGCAGCCATTGC TGGATGTTGGTGGGGCGTCATGTACGGATTTAAGGGCGTCAGCCCCAGCAACTACGAAAAA TTAGAGTATCGCAATCGCCTTGAGGAAACAGCTCGCGCACTTTACTCGCTGGGTAGTAAAGA AGACACTGTTATCTCGCTG**CTGGTGCCGCGCGGCAGC**CTCGAG

**pET rL2**<br>**[NcoI, XhoI]**	CCATGGGCGCAGTTGTTAAATGTAAACCGACATCTCCGGGTCGTCGCCACGTAGTTAAAGTG<br>GTTAACCCTGAGCTGCACAAGGGCAAACCTTTTGCTCCGTTGCTGGAAAAAAACAGCAAATC<br>CGGTGGTCGTAACAACAATGGCCGTATCACCACTCGTCATATCGGTGGTGGCCACAAGCAGG<br>CTTACCGTATTGTTGACTTCAAACGCAACAAAGACGGTATCCCGGCAGTTGTTGAACGTCTTG<br>AGTACGATCCGAACCGTTCCGCGAACATCGCGCTGGTTCTGTACAAAGACGGTGAACGCCGT<br>TACATCCTGGCCCCTAAAGGCCTGAAAGCTGGCGACCAGATTCAGTCTGGCGTTGATGCTGC<br>AATCAAACCAGGTAACACCCTGCCGATGCGCAACATCCCGGTTGGTTCTACTGTTCATAACGT<br>AGAAATGAAACCAGGTAAAGGCGGTCAGCTGGCACGTTCCGCTGGTACTTACGTTCAGATCG<br>TTGCTCGTGATGGTGCTTATGTCACCCTGCGTCTGCGTTCTGGTGAAATGCGTAAAGTAGAAG<br>CAGACTGCCGTGCAACTCTGGGCGAAGTTGGCAATGCTGAGCATATGCTGCGCGTTCTGGGT<br>AAAGCAGGTGCTGCACGCTGGCGTGGTGTTCGTCCGACCGTTCGCGGTACCGCGATGAACCC<br>GGTAGACCACCCACATGGTGGTGGTGAAGGTCGTAACTTTGGTAAGCACCCGGTAACTCCGT<br>GGGGCGTTCAGACCAAAGGTAAGAAGACCCGCAGCAACAAGCGTACTGATAAATTCATCGT<br>ACGTCGCCGTAGCAAACTCGAG

Supplementary Table 9: Primers used in this study. Corresponding restriction site in bold, underlined; mutation in bold and red

Primer	Sequence (5' to 3')
Fwd Qβ T7	TAATACGACTCACTATTATCTTGATACTACCTTTAG
Rev Qβ	CATGATCAAATTGACCCAAAGTTTCAACGCTTTACGCG
Fwd RNAI T7	TAATACGACTCACTATAACAGTATTTGGTATC
Rev RNAI	ACAAAAAAACCACCGCTACCAGCGGTGGTTTGTTTGCC
Fwd Alt NcoI	ATCGAC**CCATGG**GAGAACTTATTACAGAATTATTTGACG
Rev Alt XhoI	ATTCGA**CTCGAG**GCTGCCGCGCGGCACCAGTCCTTGAACGAACTTGTAAGGCATG
Fwd ModA NcoI	ATCGA**CCATGG**GAAAATACTCAGTAATGCAACTAAAAG
Rev ModA XhoI	ATCGTA**CTCGAG**GCTGCCGCGCGGCACCAGTAGATTAAATCCTTCAAAATCAAG
Fwd ModB NcoI	ATCGAC**CCATGG**GAATTATTAATCTTGCAGATGTTG
Rev ModB XhoI	ACTTAG**CTCGAG**GCTGCCGCGCGGCACCAGTTCAACTACCTCAATATCATAAAC
Fwd rS1 NcoI	ATCGAC**CCATGG**GAACTGAATCTTTTGCTCAACTCTTTGAAGAGTCC
Rev rS1 XhoI	ATTCGA**CTCGAG**GCTGCCGCGCGGCACCAGCTCGCCTTTAGCTGCTTTG
Fwd rS1-pTAC XhoI	ATGAAGCTTC**CTCGAG**AGACTGAATCTTTTGCTCAACTCTTTGAAGAGTCC
Rev rS1-pTAC SphI	CTCTACGT**GCATGC**ACTCGCCTTTAGCTGCTTTGAAAGCTTCAGCC
Fwd NcoI rS1 D1	ATCGAC**CCATGG**AGTCCTTAAAAGAAATCGAAACCCGCCCGGG
Rev XhoI rS1 D1	TGGTG**CTCGAG**GCTGCCGCGCGGCACCAGGGCTTCGTGACGTTTAGCTTTCTCACGGG
Fwd NcoI rS1 D2	ATCGAC**CCATGG**CCTGGATCACGCTGGAAAAAGCTTACGAAGATGCTGAAAC
Rev XhoI rS1 D2	GGTG**CTCGAG**GCTGCCGCGCGGCACCAGCTCTGCGCTGTTTTCGGATTCGATAACGGCAC
Fwd NcoI rS1 D3	ATCGAC**CCATGG**CCCGCGATCAGCTGCTGGAAAACCTGCAGGAAGG
Rev XhoI rS1 D3	TGGTG**CTCGAG**GCTGCCGCGCGGCACCAGCGGATCTTCGCCCAGCTGTTTCAGGCCCAGG
Fwd NcoI rS1 D4	ATCGAC**CCATGG**CCTGGGTAGCTATCGCTAAACGTTATCCGGAAGG

Rev XhoI rS1 D4	TGGTG**<u>CTCGAG</u>**GCTGCCGCGCGGCACCAGCGGGTTAGCTTTGCACTGTTTCAG ACCCAGGGAG
Fwd NcoI rS1 D5	ATCGAC**<u>CCATGG</u>**CCTGGCAGCAGTTCGCGGAAACCCACAACAAGGGCGACCG TGTTG
Rev XhoI S1 D5	TGGTG**<u>CTCGAG</u>**GCTGCCGCGCGGCACCAGCGGATCTTCTGCGAGCTGTTTAAC GCCCAGGGAGATACG
Fwd NcoI rS1 D6	ATCGAC**<u>CCATGG</u>**CCTTCAACAACTGGGTTGCTCTGAACAAGAAAGGCGCTATC G
Rev XhoI rS1 D6	TGGTG**<u>CTCGAG</u>**GCTGCCGCGCGGCACCAGTTTCTCGTCAGCTTCGTCTTTCGCA CGAACAGACAGG
Fwd NcoI PNPase rS1 binding	ATCGAC**<u>CCATGG</u>**CAGAAATCGAAGTGGGCCGCGTCTACACTGGTAAAGTGACC CG
Rev XhoI PNPase rS1 binding	TGGTG**<u>CTCGAG</u>**GCTGCCGCGCGGCACCAGTGCAGCAGGTTGAGACTGCTCAG TCGCTTC
Fwd ARH1 NcoI	TGCAG**<u>CCATGG</u>**AAAAATACGTCGCCGCGATG
Rev ARH1 XhoI	GTGGTG**<u>CTCGAG</u>**GCTGCCGCGCGGCACCAG
Fwd rS1 R139A	CTGGTAGACGTT**GCC**CCGGTGCGTGACACTC
Fwd rS1 R139K	CTGGTAGACGTT**AAA**CCGGTGCGTGACACTC
Rev rS1 R139	AGAACCTGGCAGGAACGCACGAATACCG
Fwd ARH1 D55,56A	GGCCGTTGGCGTGTGTCT**GCGGCG**ACTGTCATGCACTTGGC
Rev ARH1 D55,56A	AACGTCCAGAGCATCCAGCCCCCCTAA
Fwd ModB R73A	CCTTATCAATTATAT**GCG**GGTATATCAAAATCG
Rev ModB R73A	CGATTTAGTTAAATGCTTTTTAATGATTTC
Fwd ModB G74A	GACAAAAGAACTCATTAAAGATTTAC
Rev ModB G74A	GATTTTGATAT**TGCCGC**ATATAATTGATAAGGCG
Fwd ModB DS_SPCas	AATTATATCGGTTTTAGAGCTATGCTGTTTTGAATGGTCC
Rev ModB DS_SPCas	GATAAGGCGAGCTAGCACTGTACCTAGGACTGAGC
Fwd ModB amplification T4 genome	CCAAGAATGGTCATCTGGTTTATTAG

Rev ModB amplification T4 genome	CCGCCTTGGGCTCCCTGG
Fwd sequencing ModB T4 genome	CAGTTATCTATAAAAGCTGAAAG
Rev sequencing ModB T4 genome	CTTTCCAATTGGAATCATCCATTC
rpsA homologous downstream fwd	TTCTCTGACTCTTCGGGATTTTTATTC
rpsA homologous downstream rev	AGGCAAATTAAGCGGCTGCTG
Terminator region fwd	TTCTCTGACTCTTCGGGATTTTTATTC
Terminator region rev	AGGACGAAACCTGCAATCTGTC
FRT pKD4 fwd	TCGGAATAAAAATCCCGAAGAGTCAGAGAAGTCCATATGAATATCCTCCTTAG TTC
FRT pKD4 rev	GTTTACTTGACAGATTGCAGGTTTCGTCCTGTGTAGGCTGGAGCTGCTTC
5_70 left rev rpsA	AGGACGAAACCTGCAATCTGTC
5_fwd_rS1 amplification	GGCGTTGATCGTAAAAACCGC
Fwd NcoI rL2	**CCATGG**GCGCAGTTGTTAAATGTAAACCG
Rev XhoI rL2	**CTCGAG**TTTGCTACGGCGACGTACGATG
adenylated RNA-3'-adapter	/5rApp/CNNNNNNAGATCGGAAGAGCACACGTCTG/3SpC3/
RT primer	CAGACGTGTGCTCTTCCGAT
cDNA anchor fwd	ACACGACGCTCTTCCGATCTGGG
cDNA anchor rev	/5Phos/CAGATCGGAAGAGCGTCGTGTCCC/3SpC3/
qPCR acpP fwd	CGTGGTAAGACCTGCCGG
qPCR acpP rev	CTCAACGGTGTCAAGAGAATCCAAAAC

qPCR gadY fwd	GAGCACAAAGTTTCCCGTGC
qPCR gadY rev	AAACCCGGCATAGGGGACC
qPCR mcaS fwd	AAAATAGAGTCTGTCGACATCCGC
qPCR mcaS rev	CACCGGCGCAGAGGAGAC
qPCR oxyS fwd	AAAAGCGGATCCTGGAGATCC
qPCR oxyS rev	GAAACGGAGCGGCACCTC
qPCR rnaC fwd	CGTTGCGGCAACCTTGTC
qPCR rnaC rev	AAAAATATTGAGTAGCGTCAACTAC

Supplementary Table 10: Strains and plasmids used in this study.

Name	Description	Reference or resource
E. coli **strain B**	*E. coli* strain applied for bacteriophage T4 infection	DMSZ, Escherichia coli (Migula 1895) Castellani and Chalmers 1919 (DSM 613, ATCC 11303)
E. coli **strain B pTAC rS1**	*E. coli* strain B expressing His-tagged rS1 under the control of *E. coli* RNA polymerase promoter	This study
E. coli **BL21 (DE3) pET16 RNase E (1-529)**	*E. coli* strain expressing His-tagged catalytic domain of RNase E (1-529)	Plasmid was a kind gift from Prof. Dr. Ben Luisi [67]
E. coli **BL21 (DE3) pET 28 NudC V157A, E174A, E177A, E178A**	*E. coli* strain expressing His-tagged inactive Mutant of NudC	[68]
E. coli **BL21 (DE3) pET 28 rS1**	*E. coli* strain expressing His-tagged rS1	This study
E. coli **BL21 (DE3) pET 28 rS1 R139K**	*E. coli* strain expressing His-tagged rS1 R139K variant	This study
E. coli **BL21 (DE3) pET 28 rS1 R139A**	*E. coli* strain expressing His-tagged rS1 R139A variant	This study
E. coli **BL21 (DE3) pET 28 rS1 D1**	*E. coli* strain expressing His-tagged rS1 D1	This study
E. coli **BL21 (DE3) pET 28 rS1 D2**	*E. coli* strain expressing His-tagged rS1 D2	This study
E. coli **BL21 (DE3) pET 28 rS1 D2 R139K**	*E. coli* strain expressing His-tagged rS1 D2 R139K	This study
E. coli **BL21 (DE3) pET 28 rS1 D2 R139A**	*E. coli* strain expressing His-tagged rS1 D2 R139A	This study
E. coli **BL21 (DE3) pET 28 rS1 D3**	*E. coli* strain expressing His-tagged rS1 D3	This study
E. coli **BL21 (DE3) pET 28 rS1 D4**	*E. coli* strain expressing His-tagged rS1 D4	This study
E. coli **BL21 (DE3) pET 28 rS1 D5**	*E. coli* strain expressing His-tagged rS1 D5	This study

E. coli **BL21 (DE3) pET 28 rS1 D6**	*E. coli* strain expressing His-tagged rS1 D6	This study
E. coli **BL21 (DE3) pET 28 Alt**	*E. coli* strain expressing His-tagged Alt	This study
E. coli **BL21 (DE3) pET 28 ModA**	*E. coli* strain expressing His-tagged ModA	This study
E. coli **BL21 (DE3) pET 28 ModB**	*E. coli* strain expressing His-tagged ModB	This study
E. coli **BL21 (DE3) pET 28 ModB R73A, G74A**	*E. coli* strain expressing His-tagged ModB with point mutations R73A and G74A	This study
E. coli **BL21 (DE3) pET 28 NudC**	*E. coli* strain expressing His-tagged NudC	[68]
E. coli **BL21 (DE3) pET 28 PNPase S1 domain**	*E. coli* strain expressing His-tagged PNPase S1 domain	This study
E. coli **BL21 (DE3) pET 28 ARH1**	*E. coli* strain expressing His-tagged ARH1	This study
E. coli **BL21 (DE3) pET 28 ARH1 D55A, D56A**	*E. coli* strain expressing His-tagged ARH1 D55A, D56A	This study
E. coli **DHα DS_SPCas_ModB**	*E. coli* strain expressing CRISPR-Cas9 system for cleavage of *modB*	This study
E. coli **DHα DS_SPCas_ModB pET28 ModB R73A, G74A**	*E. coli* strain for editing of *modB* within T4 phage genome	This study
E. coli **BL21 (DE3) pET28 rL2**	*E. coli* strain expressing His-tagged rL2	This study
E. coli **B strain with endogenously His-tagged rS1**	*E. coli* strain with endogenous expression of rS1 with a His-tag fusion at the C-terminus	This study
**T4 WT**	Wild-type bacteriophage T4	Escherichia phage T4, DSM 4505, DSMZ, Braunschweig, Germany)
**T4 ModB R73A, G74A**	T4 phage mutant carrying inactive ModB version ModB R73A, G74A	This study

References

63 Jumper, J. *et al.* Highly accurate protein structure prediction with AlphaFold. *Nature* **596**, 583-589 (2021). https://doi.org:10.1038/s41586-021-03819-2

67 Callaghan, A. J. *et al.* Structure of Escherichia coli RNase E catalytic domain and implications for RNA turnover. *Nature* **437**, 1187-1191 (2005). https://doi.org:10.1038/nature04084

68 Cahova, H., Winz, M. L., Höfer, K., Nübel, G. & Jäschke, A. NAD captureSeq indicates NAD as a bacterial cap for a subset of regulatory RNAs. *Nature* **519**, 374-377 (2015). https://doi.org:10.1038/nature14020
