# Supplementary material for: A viral ADP-ribosyltransferase attaches RNA chains to host proteins
Source: Nature. 2023 Aug 16;620(7976):1054–62. doi: 10.1038/s41586-023-06429-2 (PMC10468400; doi:10.1038/s41586-023-06429-2)
Supplement: Supplementary file 2 — Reporting Summary [file 41586_2023_6429_MOESM2_ESM.pdf]

nature portfolio

Corresponding author(s): Dr. Katharina Höfer, Prof. Dr. Andres Jäschke

Last updated by author(s): Jun 2, 2023

Reporting Summary

Nature Portfolio wishes to improve the reproducibility of the work that we publish. This form provides structure for consistency and transparency in reporting. For further information on Nature Portfolio policies, see our Editorial Policies and the Editorial Policy Checklist.

Statistics

For all statistical analyses, confirm that the following items are present in the figure legend, table legend, main text, or Methods section.

n/a	Confirmed	
☐	☒	The exact sample size (*n*) for each experimental group/condition, given as a discrete number and unit of measurement
☐	☒	A statement on whether measurements were taken from distinct samples or whether the same sample was measured repeatedly
☐	☒	The statistical test(s) used AND whether they are one- or two-sided<br>*Only common tests should be described solely by name; describe more complex techniques in the Methods section.*
☒	☐	A description of all covariates tested
☒	☐	A description of any assumptions or corrections, such as tests of normality and adjustment for multiple comparisons
☐	☒	A full description of the statistical parameters including central tendency (e.g. means) or other basic estimates (e.g. regression coefficient) AND variation (e.g. standard deviation) or associated estimates of uncertainty (e.g. confidence intervals)
☐	☒	For null hypothesis testing, the test statistic (e.g. *F*, *t*, *r*) with confidence intervals, effect sizes, degrees of freedom and *P* value noted<br>*Give P values as exact values whenever suitable.*
☒	☐	For Bayesian analysis, information on the choice of priors and Markov chain Monte Carlo settings
☒	☐	For hierarchical and complex designs, identification of the appropriate level for tests and full reporting of outcomes
☒	☐	Estimates of effect sizes (e.g. Cohen's *d*, Pearson's *r*), indicating how they were calculated

Our web collection on statistics for biologists contains articles on many of the points above.

Software and code

Policy information about availability of computer code

Data collection	Manufacturer's Software for: MiniSeq (Illumina), ChemiDoc (Bio-Rad), Typhoon scanner (GE Healthcare), Orbitrap Exploris 480 Mass Spectrometer (Thermo Fisher Scientific), Ultimate 3000 nanoLC-system (Thermo Fisher Scientific), Dionex Ultimate 3000 RSLCnano system (Thermo Fisher Scientific), Tecan plate reader (Spark)
Data analysis	OpenMS pipeline RNPxl and OpenMS TOPPASViewer (OpenMS Version NuXL-2022-04-27), Skyline (v 21.2.0.369), MaxQuant (v 1.6.17.0 and v 2.0.3.0), Scaffold 5.2.2 for LC-MS/MS data analysis<br>bcl2fastq (v 2.20.0, Illumina), FastQC (v 0.11.9), cutadapt (v 1.18), hisat2 (v 2.2.1), samtools (v1.7), featureCounts (v 2.0.1 from Subread package), R (v 4.1.2), Integrative Genomics Viewer (IGV, v 2.4.9) for RNAylomeSeq data analysis; the custom R Script is available (https://doi.org/10.5281/zenodo.7977386)<br>AlphaFold2 (v 1.3.0 via ColabFold: https://colab.research.google.com/github/sokrypton/ColabFold/blob/main/AlphaFold2.ipynb)<br>ImageLab (v 6.1) and ImageQuant (v 5.2) for gel analysis and signal quantification<br>Microsoft Excel 2016 (v 16.0.5395.1000) for basic calculations and normalisations including in-gel digest spectrum count data from Scaffold<br>Origin Pro (v 2020b) and R (v 4.1.2 and 4.2.2) for plotting data and statistical tests

For manuscripts utilizing custom algorithms or software that are central to the research but not yet described in published literature, software must be made available to editors and reviewers. We strongly encourage code deposition in a community repository (e.g. GitHub). See the Nature Portfolio guidelines for submitting code & software for further information.

Data

Policy information about availability of data

All manuscripts must include a data availability statement. This statement should provide the following information, where applicable:

- Accession codes, unique identifiers, or web links for publicly available datasets
- A description of any restrictions on data availability
- For clinical datasets or third party data, please ensure that the statement adheres to our policy

The datasets generated during and/or analysed during the current study are available from the corresponding author on reasonable request. NGS data is accessible via GEO record GSE214431. LC-MS/MS raw data for measurements of rS1 ADP-ribosylation in vivo, in-gel digest and estimation of ModB abundance have been deposited in PRIDE under the accession PXD041714. LC-MS/MS raw data for measurements of in vitro ADP-ribosylated and RNAylated rS1 and rL2 have been deposited in PRIDE under the accession PXD038910. Reference genomes for E. coli (U00096.3, https://www.ncbi.nlm.nih.gov/nuccore/U00096.3) and T4 phage (NC_000866.4, https://www.ncbi.nlm.nih.gov/nuccore/NC_000866.4) were retrieved from NCBI. Protein structures (2MFI, 2MFL, 2KHI, 5XQ5, 2KHJ, 7K00, 6H4N) were downloaded from PDB using the indicated accession code (https://www.rcsb.org/). E. coli K12 pan proteome (UP000000625) and selected protein sequences were retrieved from Uniprot (https://www.uniprot.org/). Supplementary information is available including raw gel and blot images. Source data are provided with this paper.

Human research participants

Policy information about studies involving human research participants and Sex and Gender in Research.

Reporting on sex and gender	not applicable
Population characteristics	not applicable
Recruitment	not applicable
Ethics oversight	not applicable

Note that full information on the approval of the study protocol must also be provided in the manuscript.

Field-specific reporting

Please select the one below that is the best fit for your research. If you are not sure, read the appropriate sections before making your selection.

☒ Life sciences ☐ Behavioural & social sciences ☐ Ecological, evolutionary & environmental sciences

For a reference copy of the document with all sections, see nature.com/documents/nr-reporting-summary-flat.pdf

Life sciences study design

All studies must disclose on these points even when the disclosure is negative.

Sample size	No sample size calculation was performed. Assays were conducted in at least technical duplicates for in vitro prepared RNAs or biological triplicates (for quantification of modification) or duplicates (for qualitative assessment of effects). Sample size was determined based on similar studies in the field: Sharma S. et al. (2022) Xrn1 is a deNADding enzyme modulating mitochondrial NAD-capped RNA. Nat Commun.
Data exclusions	No data were excluded from analysis.
Replication	All statistical data shown in this manuscript is derived from at least three biologically independent replicates. Biochemical analyses shown were replicated at least twice (for qualitative effect) or three times (for quantitative effects). Mass spectrometry and RNA-Seq experiments were conducted in at least biological duplicates except for the assessment of ModB abundance and determination of RNAylation sites. All attempts of technical or biological replication were successful.
Randomization	No randomisation was necessary. Samples for biochemistry and mass spectrometry were measured sequentially, RNA-Seq was performed with all replicates at once. Images were acquired with state-of-the-art devices in an automated fashion. Humans, animals or individuals of different genders were no subjects of this study.
Blinding	No blinding was applied in this study. Blinding could not be performed, as samples were analysed or compared in a pairwise fashion or in groups using suitable controls for each experiment. In all assays of this study, the treatment cannot be disguised from the scientist.

Reporting for specific materials, systems and methods

We require information from authors about some types of materials, experimental systems and methods used in many studies. Here, indicate whether each material, system or method listed is relevant to your study. If you are not sure if a list item applies to your research, read the appropriate section before selecting a response.

Materials & experimental systems

n/a	Involved in the study	
☐	☒	Antibodies
☒	☐	Eukaryotic cell lines
☒	☐	Palaeontology and archaeology
☐	☒	Animals and other organisms
☒	☐	Clinical data
☒	☐	Dual use research of concern

Methods

n/a	Involved in the study	
☒	☐	ChIP-seq
☒	☐	Flow cytometry
☒	☐	MRI-based neuroimaging

Antibodies

Antibodies used	1. Anti-pan-ADP-ribose binding reagent (Merck, MABE1016)<br>2. Goat-anti-rabbit HRP-conjugated secondary antibody (Advansta, 541088)
Validation	please see manufactures specifications:<br>1. https://www.merckmillipore.com/DE/de/product/Anti-pan-ADP-ribose-binding-reagent,MM_NF-MABE1016<br>2. https://products.advansta.com/protein-HRP-conjugates/

Animals and other research organisms

Policy information about studies involving animals; ARRIVE guidelines recommended for reporting animal research, and Sex and Gender in Research

Laboratory animals	Escherichia coli (DSM 613, ATCC 11303), Bacteriophage T4 (DSM 4505)
Wild animals	Does not apply
Reporting on sex	Does not apply
Field-collected samples	Does not apply
Ethics oversight	Does not apply

Note that full information on the approval of the study protocol must also be provided in the manuscript.
